# New Chemistry of Chiral 1,3-Dioxolan-4-Ones

**DOI:** 10.3390/molecules28093845

**Published:** 2023-05-01

**Authors:** R. Alan Aitken, Lynn A. Power, Alexandra M. Z. Slawin

**Affiliations:** EaStCHEM School of Chemistry, University of St. Andrews, North Haugh, St. Andrews, Fife KY16 9ST, UK

**Keywords:** dioxolanone, Michael addition, Diels–Alder reaction, flash vacuum pyrolysis, X-ray structure

## Abstract

(2*S*,5*S*)-5-Phenyl-2-*t*-butyl-1,3-dioxolan-4-one, readily derived from mandelic acid, undergoes the Michael addition to butenolide and 4-methoxy-β-nitrostyrene with the absolute configuration of the products confirmed by X-ray diffraction in each case. In the former case, thermal fragmentation gives the phenyl ketone, thus illustrating use of the dioxolanone as a chiral benzoyl anion equivalent. The Diels–Alder cycloaddition chemistry of (2*S*)-5-methylene-2-*t*-butyl-1,3-dioxolan-4-one, derived from lactic acid, has been further examined with the X-ray structures of four adducts determined. In one case, thermal fragmentation of the adduct gives a chiral epoxy ketone resulting from the dioxolanone acting as a chiral ketene equivalent, while in others the products give insight into the mechanism of the dioxolanone fragmentation process.

## 1. Introduction

**Scheme 1:**
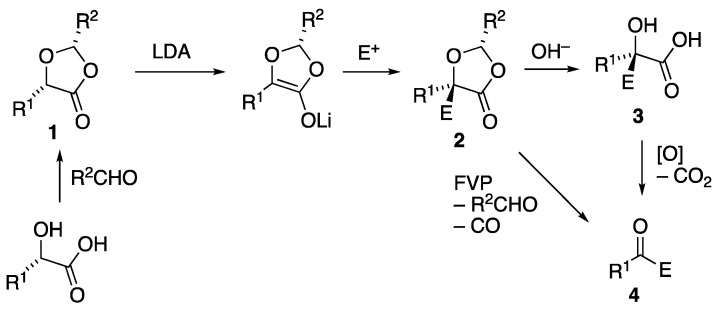
General approach for asymmetric alkylation of chiral 1,3-dioxolan-4-ones and their behaviour as acyl anion equivalents.

Starting from the seminal work of Seebach almost 40 years ago [1], chiral 1,3-dioxolan-4-ones **1** have proved very useful in asymmetric synthesis. As shown (Scheme 1), they are readily formed from α-hydroxy acids, such as lactic (R^1^ = Me) and mandelic acid (R^1^ = Ph), that are available in enantiomerically pure form and are readily purified to give pure diastereomers in which a second stereogenic centre has been introduced under control from the first. This is fortunate, since, although deprotonation results in loss of the stereochemical information at C–5, this is “stored” by the C–2 centre, and subsequent reaction with an electrophile takes place with high selectivity to give products **2**.

The initial study used not only alkyl halides as electrophiles but also aldehydes and ketones [1], and this was quickly followed by extension to nitroalkenes [2]. The new stereogenic centre created within the electrophile in these cases was also formed with high selectivity, meaning that chiral products could be obtained even after sacrificing the stereochemistry at both R^1^ and R^2^. In this way, the dioxolanones **1** could be used as chiral acyl anion equivalents [3] leading to products **4** with a stereogenic centre within the electrophilic group E. Conversion of the initial adducts **2** into **4** could be achieved either by hydrolysis to the α-hydroxy acid **3** followed by oxidative decarboxylation [4] or, in our own studies, by flash vacuum pyrolysis (FVP) with loss of the aldehyde R^2^CHO and CO [5]. Conjugate addition of a chiral dioxolanone anion to cyclopentenone later featured in the industrial scale synthesis of a muscarinic receptor antagonist [6,7,8], and around the same time Pedro and coworkers reported detailed studies in the conjugate addition of dioxolanone anions both to enones [9,10] and to nitrostyrenes [11].

**Scheme 2:**
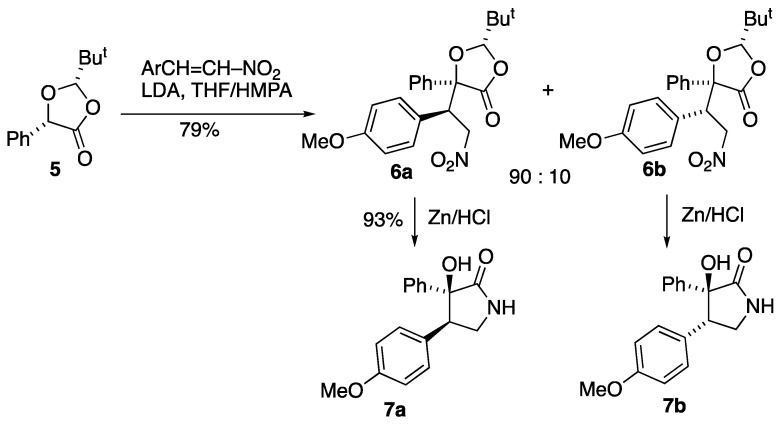
Michael addition of dioxolanone **5** to a nitrostyrene and subsequent reductive cyclisation [11].

As a specific example of the latter reaction, interaction of the anion derived from **5** with β-nitro-4-methoxystyrene gave the two diastereomeric products **6a** and **6b** in a ratio of 90:10 in an overall yield of 79%, but only in the presence of the toxic additive HMPA [11]. The stereochemistry was assigned by NOE studies on the lactam **7a** derived by reduction of the major product (Scheme 2). More recently, organocatalytic asymmetric Michael addition of a 2,2-bis(trifluoromethyl)dioxolanone to β-nitrostyrenes with excellent diastereo- and enantioselectivity using a cinchona/thiourea catalyst has been described [12].

**Scheme 3:**
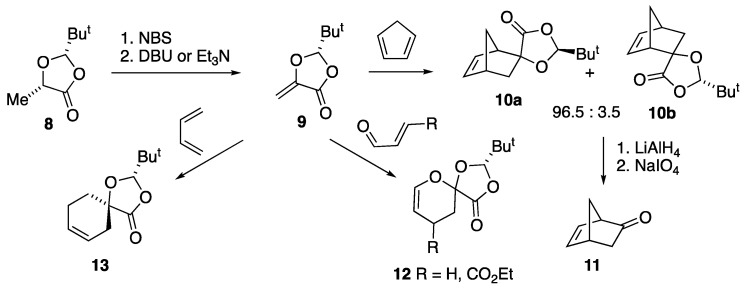
Previous work on synthesis and Diels–Alder reactivity of methylenedioxolanone **9**.

Shortly after the initial report on dioxolanone alkylation, Seebach extended the chemistry by bromination and dehydrobromination of the lactic acid-derived compound **8** to give the chiral 5-methylenedioxolanone **9** (Scheme 3) [13]. The reactivity of this as a Diels–Alder dienophile with cyclopentadiene to afford adduct **10** with high stereoselectivity was reported simultaneously by the groups of Mattay [14] and Roush [15], with the former study including confirmation of the absolute stereochemistry of the major adduct **10a** by X-ray diffraction, as well as its subsequent degradation to give chiral norbornenone **11** in 90% e.e. Roush later used a similar method to obtain the opposite enantiomer of **11** in 99% e.e. [16]. In these reactions, compound **9** is behaving as a chiral ketene equivalent and a similar approach incorporating a hydrogenation step was also patented as a route to optically active norbornan-2-one [17]. The range of dienes has been extended to include α,β-unsaturated aldehydes giving **12** [18] and butadiene, which gives **13** [19].

In this paper, we describe our studies on the Michael addition of the anion derived from dioxolanone **5** to the previously unexamined acceptor butenolide, as well as to β-nitro-4-methoxystyrene with confirmation of the adduct’s structure by X-ray diffraction in each case and unmasking of the former product by FVP to give the chiral benzoyl anion adduct. Further studies on the Diels–Alder chemistry of methylenedioxolanone **9** including functionalisation of the double bond in the cyclopentadiene adduct, and formation and structural characterisation of adducts with more sterically hindered dienes, as well as an examination of their pyrolytic behaviour, leads to greater mechanistic understanding.

## 2. Results and Discussion

### 2.1. Michael Addition of Dioxolanone ***5*** to Butenolide and β-Nitro-4-Methoxystyrene

**Scheme 4:**
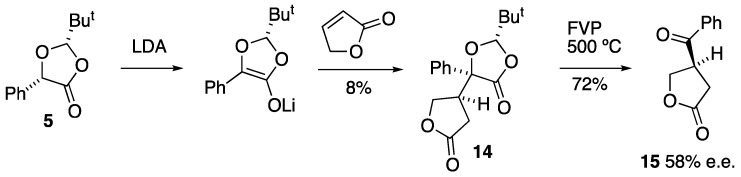
Formation and pyrolytic reactivity of adduct **14**.

**Figure 1:**
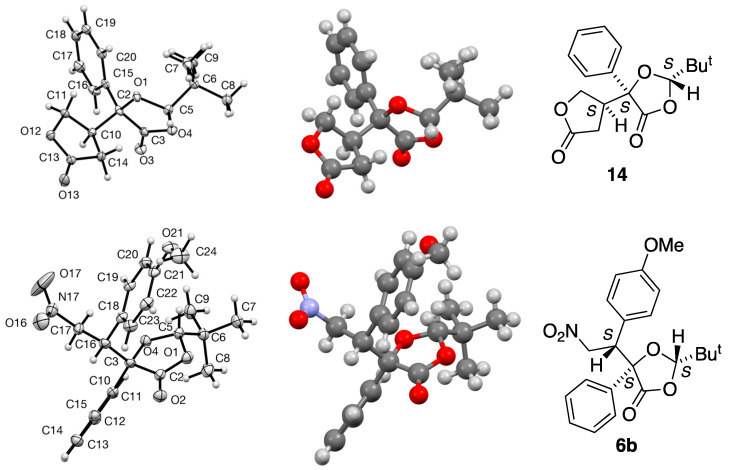
X-ray structures of **14** and **6b** with thermal ellipsoids (50%) and crystallographic numbering system used, conventional and schematic representations.

Treatment of the anion derived from dioxolanone **5** with butenolide [20] followed by chromatographic purification gave a low yield of the expected adduct **14** as a single stereoisomer (Scheme 4). This showed well-defined AB patterns of doublets at δ 4.02 and 4.17 ppm for CH_2_O and at δ 2.55 and 2.69 ppm for CH_2_C=O, both coupled to the CH between them which appeared as a multiplet at δ 3.26 ppm. The corresponding ^13^C NMR signals appeared, respectively, at δ 67.8, 29.9 and 45.3 ppm. The two lactone C=O groups gave a single IR absorption at 1789 cm^−1^ and a correct HRMS measurement was obtained. The relative and absolute configuration was confirmed by X-ray diffraction to be (*S,S,S*) (Figure 1). When this was subjected to FVP at 500 °C, there was almost complete reaction with loss of pivalaldehyde and CO to give, after preparative TLC, the lactone (4*S*)-4-benzoyltetrahydrofuran-2-one **15** in good yield. This showed good agreement with NMR chemical shift values from the literature and based on its optical rotation [21], the e.e. was 58%.

**Scheme 5:**
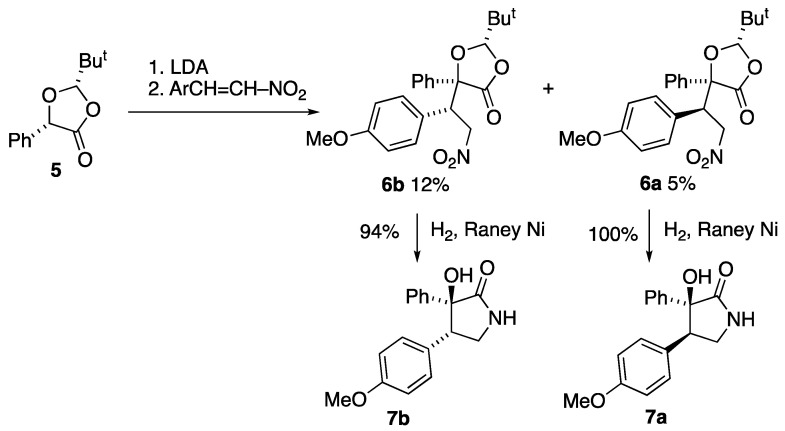
Formation and reductive cyclisation of Michael adducts **6a** and **6b**.

We now examined the Michael addition of the anion derived from **5** to β-nitro-4-methoxystyrene and, wishing to avoid the use of toxic and carcinogenic additive HMPA [11], we adopted a slightly different procedure. Rather than adding LDA to a preformed mixture of dioxolanone, nitrostyrene and HMPA as previously described [11], we added the dioxolanone to LDA at −78 °C, allowed it to warm to −20 °C and then re-cooled it to −78 °C before adding the nitrostyrene. In this way, a more equal mixture of diastereomeric products was obtained with a slight bias in favour of the opposite isomer as compared to the previous study. Although the overall yield was low, the diastereomers were readily separated by column chromatography and significant amounts of each were obtained for further study. For further structural confirmation, we were able to reduce each isomer to the respective hydroxylactam **7** in almost quantitative yield using hydrogenation with a Raney nickel catalyst (Scheme 5).

Both the spectroscopic data (see Supplementary Materials) and optical rotation of our minor product **6a** and its derived hydroxylactam **7a** were in full agreement with the literature values [11] and we were also able to characterise our major product **6b** and its derived hydroxylactam **7b** for the first time. The newly formed CH–CH_2_–NO_2_ function was confirmed by the appearance of three separate doublets of doublets in the ^1^H NMR spectra, centred at δ 5.07, 4.48 and 4.10 ppm for **6a** and at δ 4.87, 4.20 and 4.19 ppm for **6b**. The associated ^13^C NMR signals were observed at δ 52.3 (CH) and 75.0 (CH_2_) ppm for **6a** and at δ 51.8 (CH) and 74.7 (CH_2_) ppm for **6b**. For **6b,** IR absorptions were observed at 1786 (C=O), 1555 and 1380 (NO_2_) cm^−1^ and a correct HRMS measurement was obtained. The ^13^C NMR signals for the two adjacent stereocentres were observed at δ 53.5 (CH) and 78.7 (COH) ppm for **7a** and at 53.6 (CH) and 81.3 (COH) ppm for **7b**. Since Pedro’s stereochemical assignment relied only on NOE effects in the ^1^H NMR spectrum of **7a**, we thought it wise to confirm this and were able to obtain an X-ray structure of **6b** (Figure 1). Although this had a rather high R-factor owing to the presence of disordered solvent, it was sufficient to confirm the structure, which was indeed in agreement with the earlier stereochemical assignments.

### 2.2. Diels–Alder Reactions of 5-Methylenedioxolanone ***9***

**Scheme 6:**
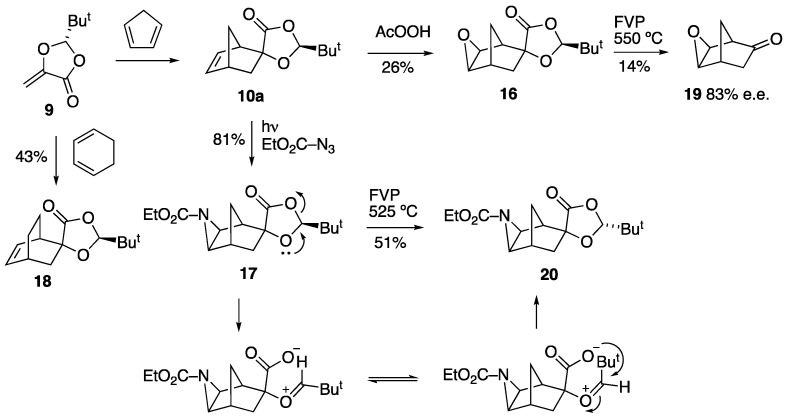
Cycloaddition of **9** with simple 1,3-dienes and subsequent chemistry.

We began by preparing the cyclopentadiene adduct **10a** by the reported method [14] and examining its fragmentation using FVP at 500 °C. However, as might have been expected, this simply resulted in a retro-Diels–Alder reaction to give cyclopentadiene, compound **9** and a little pivalaldehyde from its fragmentation. In previous studies [22], we found that an effective way to prevent cycloreversion in such cases is to functionalise the double bond as the epoxide or aziridine. Compound **10a** was therefore converted into the epoxide **16** in low yield using peroxyacetic acid and into the *N*-ethoxycarbonylaziridine **17** in good yield by photolysis in neat ethyl azidoformate [23] (Scheme 6). In these the alkene signals of **10a** had been replaced in the ^1^H NMR spectra by signals at δ 3.38 and 3.35 ppm for **16** and 2.98 and 2.88 ppm for **17** with associated ^13^C NMR signals at δ 48.2, 51.2 ppm (**16**) and 35.0, 38.7 ppm (**17**). The IR spectrum of **17** showed two separate C=O absorptions at 1789 and 1720 cm^−1^. The Diels–Alder adduct of **9** with 1,3-cyclohexadiene was also prepared using forcing conditions but the adduct **18** was formed in low yield and could not be satisfactorily purified, although spectroscopic data for the major diastereomer present were obtained.

**Figure 2:**
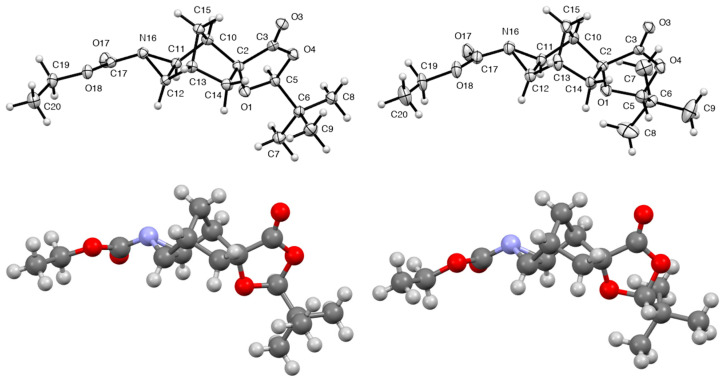
X-ray structures of **17** (**left**) and **20** (**right**) showing thermal ellipsoids (50%) with crystallographic numbering system used and conventional representations.

**Scheme 7:**
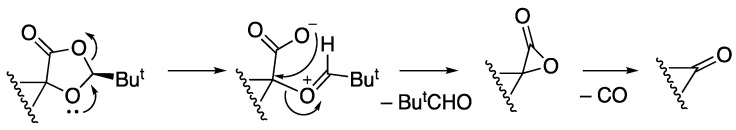
Mechanism of thermal fragmentation of dioxolanones.

Upon FVP, the epoxide **16** reacted completely at 550 °C to give the expected epoxynorbornanone **19** in low yield but high e.e. It is worth noting that the only previous route to this compound in non-racemic form involved enzymatic resolution [24]. The corresponding FVP behaviour of the aziridine **17** came as a surprise. At 525 °C it was found to convert to an extent of 50% into a new isomeric compound with very similar spectroscopic properties. The two were, however, readily separable by preparative TLC, and X-ray structure determination revealed that the new product **20** differed from **17** only in the absolute configuration of the CHBu^t^ centre (Figure 2). The most likely way in which this occurs is thermal equilibration of the dioxolanone **17** with a ring-opened zwitterionic oxonium carboxylate in which there can be rotation about the C–O^+^ bond before ring closure to give the epimeric isomer **20**. We believe that this serendipitous observation has in fact given a valuable insight into the likely mechanism of thermal fragmentation of dioxolanones in general: this normally proceeds by initial ring-opening to an oxonium carboxylate (Scheme 7) which is then followed by attack of O^−^ and the C–O^+^ carbon with expulsion of the aldehyde and formation of an α-lactone which subsequently loses CO. A further insight into this process is given by the results below.

We now decided to examine the reactivity of 5-methylenedioxolanone **9** with more sterically hindered 1,3-dienes and started with tetraphenylcyclopentadienone. This was a very reluctant reaction and after 24 h in boiling toluene much of the diene and dienophile were unchanged and the partly reduced diene, tetraphenylcyclopentenone [25], was formed by a process that is not well understood. Nonetheless, chromatographic separation allowed us to obtain a low yield of the expected adduct **21** as a single stereoisomer whose stereochemistry is assigned by analogy with that of **22a** (see below). Distinctive features confirming the structure were an AB pattern at δ 3.10 and 3.21 ppm (*J* 12.8 Hz) in the ^1^H NMR spectrum for CH_2_ with the corresponding ^13^C NMR signal at δ 42.4 ppm and two separate carbonyl absorptions at 1795 and 1782 cm^−1^ in the IR spectrum. The less hindered and more reactive diene 1,3-diphenylisobenzofuran reacted more readily and, after 36 h, chromatographic separation of the product mixture led to isolation of a major stereoisomer **22a** (25%) in pure form and a minor stereoisomer **22b** (10%) contaminated by a little **22a**. The spectra of these two stereoisomers showed significant differences with the two CH_2_ protons much more similar in **22a** (AB pattern δ 2.77, 2.86 ppm) than in **22b** (AB pattern δ 2.37, 3.14 ppm) and the position of the CHtBu signal was also markedly different (δ 4.26 ppm for **22a** vs. 5.39 ppm for **22b**). In addition, a significant amount of the byproduct 1,2-dibenzoylbenzene formed by air oxidation of diphenylisobenzofuran was obtained and characterised by comparison of its melting point [26] and NMR data [27] with values from the literature.

**Figure 3:**
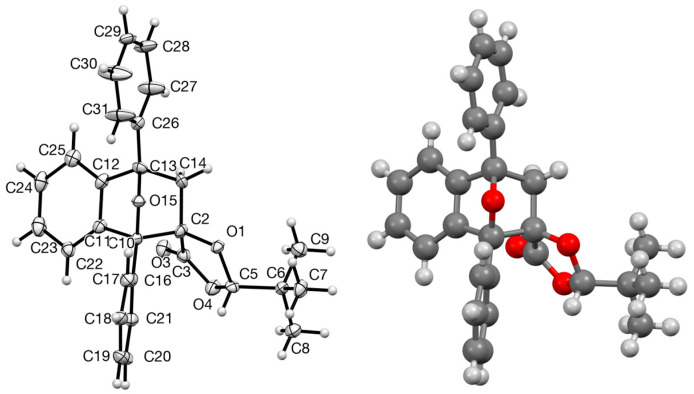
X-ray structure of **22a** showing thermal ellipsoids (50%) with crystallographic numbering system used and conventional representation.

The structure of **22a** was determined by X-ray diffraction (Figure 3) and is that resulting from addition of the diene to the face of **9** away from *tert*-butyl and with the diene overlying the lactone rather than the ether side of the ring. We assume **22b** has the structure shown resulting from addition of the diene again to the face away from *tert*-butyl but this time with the diene overlying the ether side of the ring in **9**. For both **21** and **22a** the only process observed upon FVP was a clean retro-Diels–Alder reaction to give the starting components.

**Scheme 8:**
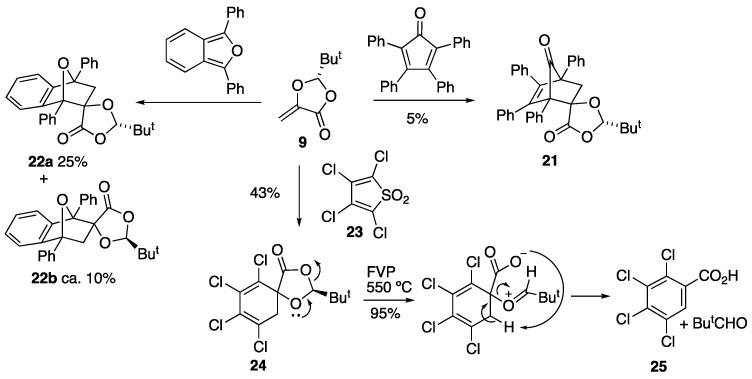
Cycloaddition of **9** with sterically hindered dienes.

We then moved on to examine the reactivity of **9** with tetrachlorothiophene 1,1-dioxide **23**, a diene known to undergo cycloaddition to a wide range of dienophiles with loss of SO_2_ [28]. The expected adduct **24** was formed in moderate yield and showed a distinctive AB pattern at δ 2.93 and 3.58 ppm (*J* 18.8 Hz) for CH_2_ in the ^1^H NMR spectrum with associated ^13^C NMR signal at δ 42.3 ppm. Upon pyrolysis, this was expected to undergo thermal fragmentation with loss of pivalaldehyde and CO to give 2,3,4,5-tetrachlorophenol. However, in reality this took a different course: upon FVP at 550 °C, only pivalaldehyde was lost to give a high yield of 2,3,4,5-tetrachlorobenzoic acid with distinctive NMR signals for the single aromatic ring CH at δ 8.09 (^1^H) and 129.2 ppm (^13^C), as well as signals for C=O at δ 164.8 ppm (^13^C NMR) and 1797 cm^−1^ (IR). We envisage this proceeding as shown (Scheme 8) with initial formation of the usual oxonium carboxylate but, rather than attacking back at the ring to form a (non-aromatic) α-lactone, this instead abstracts a proton from the adjacent position to reform the aromatic system and give the products shown.

### 2.3. Diels–Alder Reactions of the Achiral Methylenedioxolanone ***26***

**Scheme 9:**
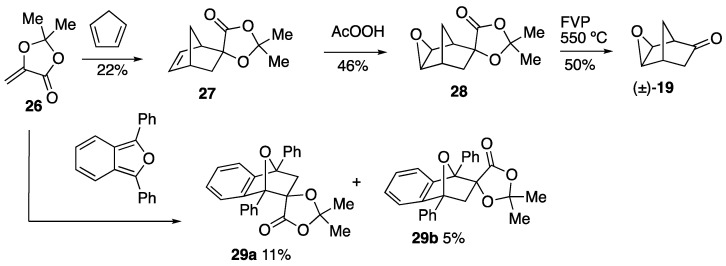
Cycloaddition chemistry of methylenedioxolanone **26**.

With a view to obtain racemic samples of some of the pyrolysis products for reference, we also examined Diels–Alder cycloaddition of 2,2-dimethyl-5-methylene-1,3-dioxolan-4-one **26**. Although this compound has been prepared in several ways [29,30], we were able to obtain it best by condensation of lactic acid and acetone followed by NBS bromination and treatment with triethylamine [18]. The Diels–Alder adduct with cyclopentadiene **27** [31] was readily formed, although in low yield, and was difficult to purify owing to partial cycloreversion to the starting components upon distillation or column chromatography. Despite this, satisfactory spectroscopic data were obtained and peroxyacetic acid oxidation afforded the epoxide **28**. In this the alkene signals of **27** (δ 6.17, 6.48 ppm in ^1^H NMR and 133.0, 140.3 ppm in ^13^C NMR) had been replaced by new signals for CH–O at δ 3.30, 3.44 ppm in ^1^H NMR and 47.9, 50.9 ppm in ^13^C NMR. When this was subjected to FVP at 550 °C, the expected fragmentation took place with loss of acetone and carbon monoxide to give the racemic epoxyketone **19** spectroscopically identical to the chiral material obtained earlier from **16** (Scheme 9).

**Figure 4:**
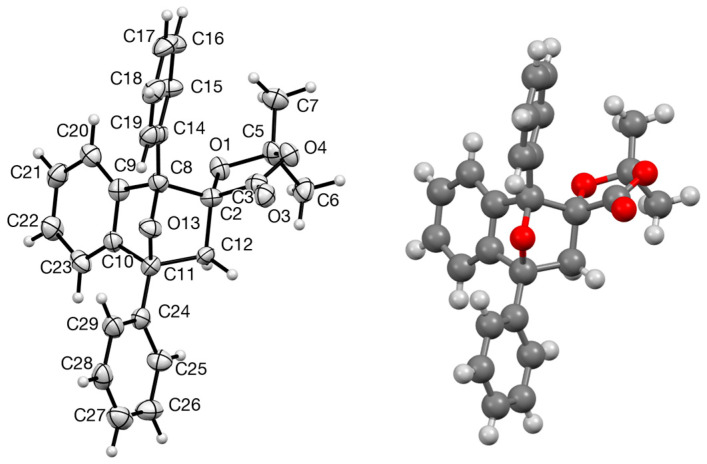
X-ray structure of **29b** showing thermal ellipsoids (50%) with crystallographic numbering system used and conventional representation.

With both faces of the ring hindered by a methyl group, **26** proved to be a poorer dienophile than **9** and reaction with diphenylisobenzofuran gave only a low yield of cycloadducts after 48 h in boiling toluene. These were separated with some difficulty by column chromatography, followed by repeated recrystallisation to give a major adduct **29a** and a minor adduct **29b**. These were readily distinguishable by ^1^H NMR with a much smaller separation of the diastereotopic CH_2_ protons in **29a** (δ 2.79, 2.91 ppm) as compared to **29b** (δ 2.41, 3.13 ppm). The associated ^13^C NMR signals were more similar appearing at δ 51.0 ppm for **29a** and 49.3 ppm for **29b**. The structure of the latter was confirmed by X-ray diffraction (Figure 4), and it can be seen that, as for **22**, cycloaddition with the diene overlying the lactone as opposed to the ether side of the dienophile ring is favoured.

## 3. Experimental

### 3.1. General Experimental Details

NMR spectra were recorded on solutions in CDCl_3_ unless otherwise stated using Bruker instruments and chemical shifts are given in ppm to high frequency from Me_4_Si with coupling constants J in Hz. On ^1^H NMR spectra signals at 0.0, 2.50 and 7.26 ppm are due to internal Me_4_Si and residual CD_3_SOCD_2_H and CHCl_3_, respectively. The ^13^C NMR spectra are referenced to the solvent signals at 77.0 (CDCl_3_) or 39.5 ppm (CD_3_SOCD_3_). IR spectra were recorded on a Perkin Elmer 1420 instrument. Elemental analysis was conducted using a Carlo Erba CHNS analyser. Mass spectra were obtained using a Micromass instrument and the ionisation method used is noted in each case. Column chromatography was carried out using silica gel of 40–63 μm particle size and preparative TLC was carried out using 1.0 mm layers of Merck alumina 60G containing 0.5% Woelm fluorescent green indicator on glass plates. Melting points were recorded on a Gallenkamp 50W melting point apparatus or a Reichert hot-stage microscope. Optical rotation measurements were made using an Optical Activity 1000 polarimeter and are given in units of 10^−1^ deg cm^2^ g^−1^.

Flash vacuum pyrolysis (FVP) was carried out in a conventional flow system by subliming the starting material through a horizontal quartz tube (30 × 2.5 cm) externally heated by a tube furnace to 500–550 °C and maintained at a pressure of 2–5 × 10^−2^ Torr by a rotary vacuum pump. Products were collected in a liquid N_2_ cooled U-shaped trap and purified as noted.

General organic and inorganic reagents and solvents were obtained from standard suppliers and used as received. Dry THF was prepared by storage over sodium wire. Starting materials (2*S*,5*S*)-2-t-Butyl-5-phenyl-1,3-dioxolan-4-one **5** [1], butenolide [20], (2*R*)-2-t-butyl-5-methylene-1,3-dioxolan-4-one **9** [13], (2*S*,5*R*,1′*R*,4′*R*)-spiro[2-t-butyl-1,3- dioxolan-4-one-5,2′-bicyclo[2.2.1]hept-5-ene] **10a** [14], tetrachlorothiophene 1,1-dioxide **23** [28] and 5-methylene-2,2-dimethyl-1,3-dioxolan-4-one **26** [18] were prepared by the reported methods.

### 3.2. Preparation and Reactions of Dioxolanone Michael Addition Products

#### 3.2.1. Preparation of (2*S*,5*S*,4′*S*)-2-*t*-Butyl-5-(2-oxo-tetrahydrofuran-4-yl)-5-phenyl-1,3-dioxolan-4-one **14**

To a solution of LDA (4.4 mmol) in dry THF (24 cm^3^) at −78 °C, (2*S*,5*S*)-2-*t*-butyl-5-phenyl-1,3-dioxolan-4-one **5** (1.0 g, 4 mmol) dissolved in dry THF (8 cm^3^) was added slowly by syringe. The reaction mixture was stirred at −78 °C for 30 min and then warmed to −30 °C for 30 min. The reaction was then re-cooled to −78 °C and butenolide (0.41 g, 4.4 mmol) was added. The reaction mixture was kept at −78 °C for 2 h, then warmed to room temperature and allowed to stir overnight. Aqueous NH_4_Cl (25 cm^3^) was added and the mixture was extracted with ether (2 × 15 cm^3^). The combined organic layers were washed with water, dried and the solvent removed to yield a yellow liquid. The product was then purified by flash column chromatography (silica gel, Et_2_O/hexane, 1:2) to give the product as a colourless solid (79.1 mg, 8%), mp 175–178 °C; [α]_D_ –24.8 (c = 0.592, CH_2_Cl_2_); ν_max_/cm^−1^ 1789, 1457, 1369, 1200, 1027 and 694; ^1^H NMR (300 MHz) δ_H_ 0.92 (9 H, s, Bu^t^), 2.55 (1 H, half AB pattern of d, *J* 17.4, 7.7, CH_2_-C=O), 2.69 (1 H, half AB pattern of d, *J* 17.4, 8.5, CH_2_-C=O), 3.21–3.32 (1 H, m, CH-CH_2_O), 4.02 (1 H, half AB pattern of d, *J* 9.7, 7.8, CH_2_-O), 4.17 (1 H, half AB pattern of d, *J* 9.7, 7.0, CH_2_-O), 5.46 (1 H, s, CH-Bu^t^), 7.36–7.46 (3 H, m, Ph) and 7.64–7.70 (2 H, m, Ph); ^13^C NMR (75 MHz) δ_C_ 23.4 (Bu^t^), 29.9 (CH_2_), 35.8 (C-Me_3_), 45.3 (CH), 67.8 (CH_2_), 80.7 (C), 111.4 (CH-Bu^t^), 124.8 (2CH), 128.7 (2CH), 128.9 (CH), 135.5 (C), 171.7 (C=O) and 175.0 (C=O); HRMS (ES^+^): found 327.1200. C_17_H_20_NaO_5_ (M + Na) requires 327.1208.

#### 3.2.2. Pyrolysis of (2*S*,5*S*,4′*S*)-2-*t*-Butyl-5-(2-oxo-tetrahydrofuran-4-yl)-5-phenyl-1,3-dioxolan-4-one **14**

FVP of the reactant **14** (24.0 mg, 78.8 µmol) was performed at 500 °C and 3.8 × 10^−2^ Torr. Preparative TLC (silica, diethyl ether) was used to separate the product from some unchanged starting material, giving the product (4*S*)-4-benzoyltetrahydrofuran-2-one **15** as a yellow oil (10.8 mg, 72%). [α]_D_ −11.3 (c = 0.56, CHCl_3_), [lit. [21], –19.6 (c = 1.08, CHCl_3_)], e.e.= 58%; ^1^H NMR (300 MHz) δ_H_ 2.81 (1 H, half AB pattern of d, *J* 17.8, 9.3, CH_2_-C=O), 3.03 (1 H, half AB pattern of d, *J* 17.8, 7.4, CH_2_-C=O), 4.34–4.44 (1 H, m, CH), 4.48 (1 H, half AB pattern of d, *J* 9.0, 6.7, CH_2_O), 4.63 (1 H, half AB pattern of d, *J* 9.0, 8.5, CH_2_O), 7.50–7.57 (2 H, m, Ph), 7.62–7.68 (1 H, m, Ph) and 7.91–7.96 (2 H, m, Ph); ^13^C NMR (75 MHz) δ_C_ 30.9 (CH_2_-C=O), 42.1 (CH), 68.9 (CH_2_O), 128.5 (2CH), 129.1 (2CH), 134.2 (CH), 134.8 (C), 175.4 (C=O) and 196.2 (C=O).

#### 3.2.3. Preparation of (2*S*,5*S*)-2-*t*-Butyl-5-(1′-(4-methoxyphenyl)-2′-nitroethyl)-5-phenyl-1,3-dioxolan-4-one **6a**/**6b** [11]

To a solution of LDA (22 mmol) in dry THF (100 cm^3^) at −78 °C, a solution of (2*S*,5*S*)-2-*t*-butyl-5-phenyl-1,3-dioxolan-4-one **5** (4.40 g, 20 mmol) in dry THF (30 cm^3^) was added slowly by syringe. The solution was stirred at −78 °C for 30 min, then warmed up to −20 °C for 30 min and then re-cooled to −78 °C. A solution of 1-methoxy-4-[(*E*)-2-nitrovinyl]benzene (3.94 g, 22 mmol) in dry THF (20 cm^3^) was then added slowly by syringe and the mixture was stirred at −78 °C for 2 h and then warmed up to room temperature and stirred overnight. Aqueous NH_4_Cl (100 cm^3^) was added and the mixture was extracted with ether (2 × 60 cm^3^). The combined organic layers were washed with water, dried and the solvent removed to yield a viscous brown liquid. ^1^H NMR spectroscopic analysis showed that the diastereomers were present in a 3:4 ratio along with a number of impurities. Separation was achieved by flash column chromatography (silica gel, Et_2_O/hexane, 1:5). This gave first the minor product **6a** (0.41 g, 5%) and on removal of solvent this was recrystallised to give a pure product (0.24 g, 3%) as colourless prism-like crystals. This was followed by the major product **6b** as colourless crystals (1.01 g, 12%).

Minor isomer, (2*S*,5*S*,1′*R*)-2-*t*-butyl-5-(1′-(4-methoxyphenyl)-2′-nitroethyl)-5-phenyl-1,3-dioxolan-4-one **6a**: mp 98–102 °C, (lit. [11], 96–98 °C); [α]_D_ –61.07, (c = 1.5, CHCl_3_) [lit. [11], −54.2, (c = 1.5, CHCl_3_)]; (Found: C, 66.4; H, 6.4; N, 3.5. C_22_H_25_NO_6_ requires C, 66.2; H, 6.3; N, 3.5%); ν_max_/cm^−1^ 1778, 1612, 1559, 1513, 1424, 1404 and 1350; ^1^H NMR (300 MHz) δ_H_ 0.71 (9 H, s, Bu^t^), 3.81 (3 H, s, OMe), 4.10 (1 H, dd, *J* 11.1, 4.7, CH_2_), 4.43 (1H, s, HC-Bu^t^), 4.48 (1 H, dd, *J* 13.5, 4.7, CH_2_), 5.07 (1 H, dd, *J* 13.5, 11.1, CH-C_6_H_4_-OMe), 6.86 (2 H, d, *J* 8.7, Ar H-3, H-5), 7.20 (2 H, d, *J* 8.7, Ar H-2, H-6), 7.36–7.42 (3 H, m, Ph) and 7.59–7.64 (2 H, m, Ph); ^13^C NMR (75 MHz) δ_C_ 23.1 (Bu^t^), 35.0 (C-Me_3_), 52.3 (CH-CH_2_-NO_2_), 55.2 (MeO), 75.0 (CH_2_), 83.6 (C), 110.6 (C-Bu^t^), 114.2 (2CH), 125.1 (C), 125.5 (2CH), 128.7 (2CH), 129.0 (2CH), 130.2 (CH), 136.1 (C), 159.8 (C), and 171.3 (C=O); *m*/*z* 422.11 (M^+^ + Na^+^, 100%).

Major isomer, (2*S*,5*S*,1′*S*)-2-*t*-butyl-5-(1′-(4-methoxyphenyl)-2′-nitroethyl)-5-phenyl-1,3-dioxolan-4-one **6b**: mp 139–140 °C; [α]_D_ +37.6 (c = 1, CH_2_Cl_2_) (Found: C, 66.5; H, 6.2; N, 3.6. C_22_H_25_NO_6_ requires C, 66.2; H, 6.3; N, 3.5%); ν_max_/cm^−1^ 1786, 1611, 1585, 1555, 1514, 1459, 1450, 1436, 1380, 1350, 1301, 1252, 1200 and 1184; ^1^H NMR (300 MHz) δ_H_ 0.84 (9 H, s, Bu^t^), 3.82 (3 H, s, OMe), 4.19 (1 H, dd, *J* 14.2, 4.4, CH_2_), 4.20 (1H, dd, *J* 12.8, 4.4, CH_2_), 4.65 (1 H, s, HC-Bu^t^), 4.87 (1 H, dd, *J* 14.2, 12.7, CH-C_6_H_4_-OMe), 6.91 (2 H, d, *J* 8.8, Ar H-3, H-5), 7.35 (2 H, d, *J* 8.8, Ar H-2, H-6), 7.38–7.50 (3 H, m, Ph) and 7.78–7.83 (2 H, m, Ph); ^13^C NMR (75 MHz) δ_C_ 23.5 (Bu^t^), 35.4 (C-Me_3_), 51.8 (CH-CH_2_-NO_2_), 55.3 (MeO), 74.7 (CH_2_), 84.1 (C), 110.9 (C-Bu^t^), 114.5 (2CH), 124.5 (C), 125.0 (2CH), 128.9 (2CH), 129.0 (2CH), 130.4 (CH), 135.9 (C), 160.1 (C) and 171.4 (C=O); HRMS (ES^+^): found 422.1584. C_22_H_25_NaNO_6_ (M + Na) requires 422.1580.

#### 3.2.4. Reduction of **6b** Using Raney Ni under Hydrogen to Give the γ-Lactam (3*S*,4*S*)-3-Hydroxy-4-(4-methoxyphenyl)-3-phenylpyrrolidin-2-one **7b**

Raney Ni (6.00 g) and **6b** (0.51 g, 1.27 mmol) were stirred in methanol (20 cm^3^) in a hydrogenation flask under hydrogen gas until the required amount of gas (85.3 cm^3^) was consumed. The mixture was filtered through a layer of Celite to remove the nickel. The filtrate was concentrated to yield the product **7b** (0.34 g, 94%) as a white powder, mp 226–227 °C; [α]_D_ −161.2 (c = 0.5, MeOH); (Found: C, 71.7; H, 6.0; N, 5.0. C_17_H_17_NO_3_ requires C, 72.1; H, 6.1; N, 4.9%); ν_max_/cm^−1^ 3301, 1772, 1612, 1516, 1464 and 1377; ^1^H NMR (300 MHz) δ_H_ 3.53 (1 H, m, CH_2_), 3.60 (1 H, dd, *J* 10.3, 7.8, CH_2_), 3.72 (3 H, s, OMe), 3.89 (1 H, dd, *J* 10.3, 7.8, CH), 6.49 (1 H, br s, NH or OH), 6.61–6.62 (2 H, m, Ar), 6.69–6.74 (2 H, m, Ar), 6.99–7.04 (2 H, m, Ph) and 7.14–7.20 (3 H, m, Ph); ^13^C NMR (75 MHz) δ_C_ 42.8 (CH_2_), 53.6 (CH), 55.2 (OMe), 81.3 (C-OH), 113.3 (2CH), 126.1 (2CH), 127.5 (C), 127.9 (CH), 128.0 (2CH), 129.6 (2CH), 137.9 (C), 158.7 (C) and 178.3 (C=O); *m*/*z* (ES) 306.14 (M + Na^+^, 100%).

#### 3.2.5. Reduction of **6a** Using Raney Ni under Hydrogen to Give the γ-Lactam, (3*S*,4*R*)-3-Hydroxy-4-(4-methoxyphenyl)-3-phenylpyrrolidin-2-one **7a**

Raney Ni (6.00 g) and **6a** (0.45 g, 1.13 mmol) were stirred in methanol (20 cm^3^) as for the formation of **7b**. The filtrate was concentrated to yield the product **7a** (0.32 g, 100%) as a white powder, mp 220–222 °C (lit. [11], 220–222 °C); [α]_D_ −38.4 (c = 0.5, MeOH) [lit. [11], –37.9 (c = 0.5 MeOH)]; (Found: C, 72.2; H, 5.7; N, 5.1. C_17_H_17_NO_3_ requires C, 72.1; H, 6.1; N, 4.9%); ν_max_/cm^−1^ 3327, 1700, 1517, 1378, 1248, 1178 and 1028; ^1^H NMR (300 MHz) δ_H_ (DMSO-d_6_) 3.45 (2 H, m, CH_2_), 3.60 (1 H, t, *J* 6.76, CH), 3.69 (3 H, s, OMe), 5.98 (1 H, s, NH), 6.75 (2 H, d, *J* 8.7, Ar), 6.97 (2 H, d, *J* 8.7, Ar), 7.17–7.31 (5 H, m, Ph) and 8.21 (1 H, s, OH); ^13^C NMR (75 MHz) δ_C_ (DMSO-d_6_) 44.0 (CH_2_), 53.5 (CH), 54.9 (Ome), 78.7 (C-OH), 112.9 (2CH), 126.4 (2CH), 126.7 (CH), 127.3 (2CH), 127.9 (C), 130.5 (2CH), 142.4 (C), 158.1 (C) and 176.1 (C=O); *m*/*z* (ES) 306.09 (M + Na^+^, 100%).

### 3.3. Preparation and Reactions of Diels–Alder Adducts of (2R)-2-t-Butyl-5-methylene-1,3-dioxolan-4-one ***9***

#### 3.3.1. Preparation of (2*S*,5*R*,1′*R*,2′*R*,4′*R*,5′*R*)-Spiro[2-*t*-butyl-1,3-dioxolan-4-one-5,6′-3′-oxatricyclo[3.2.1.0^2,4^]octane] **16**

The alkene **10a** (0.78 g, 3.5 mmol) was stirred in CH_2_Cl_2_ (25 cm^3^) for 5 min. Sodium carbonate (5 g) was then added followed by addition of peracetic acid (40%, 0.532 g, 7.0 mmol) in acetic acid. The reaction proceeded immediately with vigorous bubbling and the mixture was allowed to stir for 3 days. The mixture was filtered to remove the sodium acetate and the solvent was then removed to yield the product as colourless oil (0.67 g).^1^H NMR spectroscopic analysis indicated that the product had been formed in a 2:1 ratio with the starting material. The crude product was separated by flash column chromatography (silica gel, Et_2_O:hexane, 1:3) giving the product (0.22 g, 26%) as a colourless liquid. ν_max_/cm^−1^ 1787, 1639, 1485, 1407, 1348, 1289, 1245, 1208 and 1175; ^1^H NMR (300 MHz) δ_H_ 0.95 (9 H, s, Bu^t^), 1.39–1.42 (1 H, dd, *J* 3.7, 1.8, H_2_C-8′), 1.45–1.47 (1 H, m, H_2_C-8′), 1.53 (1 H, dd, *J* 13.4, 3.8, H_2_C-7′), 2.21 (1 H, dd, *J* 13.4, 4.1, H_2_C-7′), 2.60 (1 H, dd, *J* 3.7, 1.6, HC-1′), 2.83 (1H, q, *J* 1.3, HC-5′), 3.35 (1 H, half AB pattern of d, *J* 3.7, 1.0, HC-4′), 3.38 (1 H, half AB pattern of d, *J* 3.6, 1.1, HC-2′) and 5.17 (1 H, s, HC-Bu^t^); ^13^C NMR (75 MHz) δ_C_ 23.2 (Bu^t^), 24.0 (CH_2_), 34.4 (C-Me_3_), 36.2 (CH), 37.1 (CH_2_), 42.9 (CH), 48.2 (CH), 51.2 (CH), 85.1 (C), 108.0 (Bu^t^-C) and 175.1 (C=O); *m*/*z* (ES) 261.20 (M + Na^+^, 3%), M^+^ 100%, 281.15; M^+^ 23%, 195.07.

#### 3.3.2. Preparation of (2*S*,5*R*,1′*R*,2′*R*,4′*R*,5′*R*)-Spiro[2-*t*-butyl-1,3-dioxolan-4-one-5,6′-3′-ethoxycarbonyl-3′-azatricyclo[3.2.1.0^2,4^]octane] **17**

A mixture of **10a** (0.55 g, 2.4 mmol) and ethyl azidoformate (0.828 g, 7.2 mmol) was placed in a quartz tube and positioned 15 cm from a 400 W medium pressure mercury lamp. After 10 min irradiation the solution changed colour from colourless to pale yellow and a stream of N_2_ gas was released. The reaction was left on for approximately 8 h until the starting material was judged to be consumed (TLC). The crude product was purified by flash column chromatography (silica gel, dry loaded, Et_2_O:hexane, 1:1), to furnish the product (0.6 g, 81%) as pale yellow crystals mp 90–91 °C [α]_D_ +61.76 (c = 0.102, CH_2_Cl_2_); (Found C, 62.0; H, 7.3; N, 4.2. C_16_H_23_NO_5_ requires C, 62.1; H, 7.5; N, 4.5%); ν_max_/cm^−1^ 1789, 1720, 1406, 1378, 1298, 1278, 1265, 1228, 1187 and 1173; δ_H_ 0.95 (9 H, s, Bu^t^), 1.28 (3 H, t, *J* 7.0, CH_3_), 1.45–1.60 (3 H, m, H_2_C-7′, H_2_C-8′), 2.17 (1 H, dd, *J* 12.9, 4.0, H_2_C-7′), 2.62–2.67 (1 H, m, HC-1′), 2.86–2.87 (1 H, m, HC-5′), 2.88 (1 H, m, HC-2′), 2.96–3.00 (1 H, m, HC-4′), 4.15 (2 H, q, *J* 7.0, CH_2_CH_3_) and 5.16 (1 H, s, CH-Bu^t^); δ_C_ 14.3 (CH_3_ of OEt), 23.2 (Bu^t^), 25.7 (C-8′), 34.4 (C-Me_3_), 35.0 (HC-2′), 35.4 (C-1′), 37.5 (C-7′), 38.7 (HC-4′), 42.1 (C-5′), 62.5 (CH_2_ in OEt), 84.6 (C-6′/C-5), 108.0 (CH-Bu^t^), 162.1 (N-C=O) and 174.9 (C=O); HRMS (ES^+^): found 332.1474. C_16_H_23_NaNO_5_ (M + Na) requires 332.1474.

#### 3.3.3. Preparation of Spiro[2-*t*-Butyl-1,3-dioxolan-4-one-5,2′-bicyclo[2.2.2]oct-5′-ene] **18**

A solution of **9** (1.51 g, 9.7 mmol) and 1,3-cyclohexadiene (0.77 g, 9.6 mmol) in toluene (15 cm^3^) was heated in a 50 cm^3^ autoclave at 150 °C for 84 h. Once the reaction mixture had cooled the contents of the flask were washed into an evaporating flask using CH_2_Cl_2_ and the solvent was then removed to yield a brown liquid (2.26 g). The ^1^H NMR spectrum showed that the product had formed but further purification was required. This was attempted by flash column chromatography (silica gel, Et_2_O/hexane, 1:4) to give the product (0.98 g, 43%) as a yellow oil with a strong sweet smell; ν_max_/cm^−1^ 1789, 1407, 1367, 1342, 1289, 1252, 1178 and 1113; δ_H_ 0.92 (9 H, s, Bu^t^), 1.11–1.21 (1 H, m), 1.24–1.31 (1H, m), 1.54–1.58 (1 H, m), 1.58–1.61 (1 H, m), 1.86–1.96 (1 H, m), 2.07 (1 H, dd, *J* 13.8, 2.2), 2.68–2.76 (1 H, m), 2.90–2.96 (1 H, m), 6.18–6.24 (1 H, m) and 6.4–6.46 (1 H, m); additional peaks present at 1.0, 1.23, 2.85–2.91 (m) and 5.18 indicated presence of other diastereomers; δ_C_ 19.0 (CH_2_), 23.3 (Bu^t^), 23.5 (CH_2_), 29.8 (CH), 33.6 (CH), 34.6 (C-Me_3_), 39.2 (CH_2_), 82.1 (C), 107.4 (C-Bu^t^), 130.7 (=CH), 135.2 (=CH), C=O not apparent. *m*/*z* (CI) 237.15, (M + H^+^, 100%); HRMS (CI): found 237.1494. C_14_H_21_NO_3_ (M + H) requires 237.1491.

#### 3.3.4. Pyrolysis of (2*S*,5*S*,1′*R*,5′*R*)-Spiro[2-*t*-butyl-1,3-dioxolan-4-one-5,6′-3′-oxatricyclo[3.2.1.0^2,4^]octane] 16 to give 3-oxatricyclo[3.2.1.0^2,4^]octan-6-one **19**

FVP of **16** (0.207g, 0.86 mmol) was performed at 550 °C and 3.8 × 10^−2^ Torr. Purification of the material in the cold trap using preparative TLC (SiO_2_, Et_2_O/hexane, 1:3) to give the product **19** (0.015 g, 14%) as a white solid at R_f_ ~0.25–0.4; [α]_D_ +310 (c = 0.22, CHCl_3_), [lit. [24], +321 (c = 2.1, CHCl_3_) for 86% e.e.], e.e. 83%; δ_H_ 1.21–1.27 (1 H, br m, H_2_C-8), 1.67–1.75 (1 H, m, H_2_C-8), 1.92 (1 H, dd, *J* 17.7, 4.7, H_2_C-7), 2.11 (1 H, dd, *J* 17.7, 3.9, H_2_C-7), 2.80–2.83 (1 H, m, HC-1), 2.85–2.88 (1 H, m, HC-5), 3.27–3.30 (1 H, m, HC-2) and 3.42–3.46 (1 H, m, HC-4); δ_C_ 25.0 (CH_2_), 35.1 (CH), 39.6 (CH_2_), 46.9 (CH), 50.8 (CH–O), 51.8 (CH–O) and 211.8 (C=O).

#### 3.3.5. Pyrolysis of (2*S*,5*R*,1′*R*,2′*R*,4′*R*,5′*R*)-Spiro[2-*t*-Butyl-1,3-dioxolan-4-one-5,6′-3′-ethoxycarbonyl-3′-azatricyclo[3.2.1.0^2,4^]octane **17** giving **20**

FVP of **17** (0.1959 g, 0.6 mmol) was performed at 525 °C and 3.5 × 10^−2^ Torr. ^1^H NMR spectroscopic analysis indicated a 1:1 ratio of unchanged starting material and a new product. Purification was achieved using preparative TLC (SiO_2_, Et_2_O/hexane, 1:1). The product (Rf ~0.55–0.65) proved to be (2*R*,5*R*,1′*R*,2′*R*,4′*R*,5′*R*)-spiro[2-*t*-butyl-1,3-dioxolan-4-one-5,6′-3′-ethoxycarbonyl-3′-azatricyclo[3.2.1.0^2,4^]octane] **20** (0.1 g, 51%) obtained as a colourless solid, mp 122–123 °C; [α]_D_ –18.86 (c = 0.082, CH_2_Cl_2_); ν_max_/cm^−1^ 1789, 1720; δ_H_ 0.97 (9 H, s, Bu^t^), 1.29 (3 H, t, *J* 7.1, CH_3_), 1.46–1.63 (2 H, m), 1.63–1.72 (1 H, m), 2.00 (1 H, dd, *J* 12.6, 4.2, H_2_C-7′), 2.61–2.68 (1 H, m), 2.76 (1 H, m), 2.82–2.89 (1 H, m), 3.10 (1 H, m), 4.16 (2 H, q, *J* 7.1, CH_2_-CH_3_) and 5.10 (1 H, s, CH-Bu^t^); δ_C_ 14.3 (CH_3_ ofEt), 23.3 (Bu^t^), 25.7 (CH_2_), 34.6 (C-Me_3_), 34.8 (CH), 35.8 (CH), 37.7 (CH_2_), 38.2 (CH), 44.7 (CH), 62.5 (CH_2_ of Et), 84.7 (C-5/C-6′), 108.7 (CH-Bu^t^), 162.1 (N-C=O) and 174.9 (C=O); *m*/*z* (CI) 310.17 (M + H, 10%), 224.09 (100) and 196 (65); HRMS (CI): found 310.1653. C_16_H_24_NO_5_ (M + H) requires 310.1654.

#### 3.3.6. Preparation of Spiro[2-*t*-Butyl-1,3-dioxolan-4-one-5,2′-1′,4′,5′,6′-tetraphenyl-7′-oxobicyclo[2.2.1]hept-5′-ene] **21**

A solution of **9** (3.50 g, 22.4 mmol) and tetraphenylcyclopentadienone (8.61 g, 22.4 mmol) in dry toluene (25 cm^3^) was heated under reflux for 24 h. Insoluble material (0.69 g) was removed by filtration and evaporation; the filtrate gave a purple solid. ^1^H NMR spectroscopic analysis showed a large amount of the starting diene remained. Attempted purification by flash column chromatography (dry loaded; SiO_2_, Et_2_O/hexane, 1:2) gave a fraction from which pale purple crystals (0.53 g) precipitated, mp 156–158 °C, which were identified as the partially reduced starting material 2,3,4,5-tetraphenylcyclopent-2-enone (lit. [25], 160–163 °C) which was the major product of the reaction; ν_max_/cm^−1^ 1692, 1550 and 1463; δ_H_ 3.75 (1 H, d, *J* 2.5, 4-H), 4.56 (1 H, d, *J* 2.5, 5-H) and 6.89–7.34 (20 H, m, Ph); δ_C_ 57.6 (C-4) 63.0 (C-5), 127.0 (Ph), 127.1 (Ph), 127.5 (Ph), 127.7 (Ph), 128.1 (Ph), 128.2 (Ph), 128.4 (Ph), 128.9 (Ph), 129.0 (Ph), 129.4 (Ph), 129.8 (Ph), 131.7 (C1-Ph), 134.4 (C1-Ph), 139.3 (C1-Ph), 140.0 (C1-Ph), 141.4 (C-CO), 168.9 (C-3) and 205.9 (C=O); *m*/*z* (ES) 409.13 (M+Na^+^, 100%); HRMS (ES): found 409.1586. C_29_H_22_NaO (M + Na) requires 409.1568.

The filtrate from these fractions was concentrated to yield a purple solid (0.45 g), which was recrystallised from toluene to yield the desired product (0.06 g, 5%) as purple crystals mp 178–180 °C; [α]_D_ –292.5 (c =0.04, CH_2_Cl_2_); ν_max_/cm^−1^ 1795, 1782, 1377, 1242 and 1139; δ_H_ 0.74 (9 H, s, Bu^t^), 3.10 and 3.21 (2 H, AB pattern, *J* 12.8, CH_2_), 3.61 (1 H, s, CH-Bu^t^), 6.66–6.72 (2 H, m, Ph), 6.86–6.97 (6 H, m, Ph), 7.06–7.16 (3 H, m, Ph), 7.16–7.31 (7 H, m, Ph) and 7.52–7.57 (2 H, m, Ph); δ_C_ 23.0 (Bu^t^), 34.5 (CMe_3_), 42.4 (C-3′), 62.0 (C-4′), 87.0 (C-2′/C-5), 109.7 (C-Bu^t^), 127.1 (Ph), 127.2 (Ph), 127.3 (Ph), 127.5 (Ph), 127.6 (Ph), 127.8 (Ph), 127.9 (Ph), 128.0 (Ph), 128.2 (Ph), 128.5 (Ph), 129.1 (Ph), 129.3 (Ph), 130.1 (Ph), 130.9 (Ph), 132.1 (Ph), 133.4 (Ph), 145.2 (C-ipso), 174.0 (C=O) and 197.5 (C-7′) [C-2′/C-5, C-4′ C-ipso, C=O and C-7′ were assigned by HMBC]; *m*/*z* (ES) 563.22 (M+ Na^+^, 10%) and 407.13 (M^+^–CO and dioxolanone, 100%); HRMS (ES): found 563.2197. C_37_H_32_NaO_4_ (M + Na) requires 563.2198.

#### 3.3.7. Preparation of Spiro[2-*t*-Butyl-1,3-dioxolan-4-one-5,9′-1′,8′-diphenyl-11′-oxatricyclo[6.2.1.0^2,7^]undeca-2′,4′,6′-triene] **22a** and **22b**

A solution of **9** (3.11 g, 19.9 mmol) and 1,3-diphenylisobenzofuran (4.85 g, 17.9 mmol) in toluene (30 cm^3^) was heated under reflux for 36 h, then cooled and the solvent was removed to yield a bright yellow solid (7.86 g). ^1^H NMR spectroscopic analysis indicated that the product had formed; however, there was still a large amount of diene remaining. The material was subjected to flash column chromatography (dry loaded; SiO_2_, Et_2_O/hexane, 1:2), followed by preparative TLC (Et_2_O/hexane, 1:3) to give the major product, (2*S*,5*R*,1′*R*,8′*S*)-spiro[2-*t*-butyl-1,3-dioxolan-4-one-5,9′-1′,8′-diphenyl-11′-oxatricyclo[6.2.1.0^2,7^]undeca-2′,4′,6′-triene] **22a** (1.58 g, 25%) as pale yellow crystals, mp 155–156 °C; [α]_D_ –16.4 (c = 1, CH_2_Cl_2_); (Found: C, 79.45; H, 6.45. C_28_H_26_O_4_ requires C 78.9; H, 6.2%); ν_max_/cm^−1^ 1795, 1345, 1311, 1285, 1243 and 1143; δ_H_ 0.76 (9 H, s, Bu^t^), 2.77 and 2.86 (2 H, AB pattern, *J* 12.3, H_2_C-10′), 4.26 (1 H, s, CH-Bu^t^), 6.90–6.96 (1 H, m, Ph), 7.14–7.29 (4 H, m, Ph), 7.42–7.55 (5 H, m, Ph), 7.60–7.67 (2 H, m, Ph) and 7.74–7.80 (2 H, m, Ph); δ_C_ 23.0 (Me_3_), 34.8 (C-Me_3_), 49.4 (CH_2_), 88.0 (C-1′ or C-8′), 88.2 (C-1′ or C-8′), 93.8 (C-9′/C-5), 109.4 (CH-Bu^t^), 118.7 (CH), 122.2 (CH), 125.5 (2CH), 126.3 (2CH), 126.7 (CH), 128.0 (CH), 128.3 (2CH), 128.5 (2CH), 128.6 (2CH), 134.7 (C), 137.2 (C), 141.3 (C), 149.3 (C) and 171.9 (C=O).

The minor diastereomer, (2*S*,5*R*,1′*R*,8′*R*)-spiro[2-*t*-butyl-1,3-dioxolan-4-one-5,9′-1′,8′-diphenyl-11′-oxatricyclo[6.2.1.0^2,7^]undeca-2′,4′,6′-triene] **22b** (probable stereochemistry shown), was obtained in a mixed fraction with the major diastereomer. It could not be isolated in pure form but the following data was obtained: δ_H_ 0.84 (9 H, s, Bu^t^), 2.37 (1 H, d, *J* 11.9, HC-10′), 3.14 (1 H, d, *J* 11.9, HC-10′), 5.39 (1 H, s, CH-Bu^t^), 7.00 (1 H, m, Ph), 7.20–7.26 (4 H, m, Ph), 7.40–7.55 (5 H, m, Ph), 7.59–7.65 (2 H, m, Ph) and 7.72–7.80 (2 H, m, Ph); δ_C_ 23.2 (Me_3_), 35.8 (C-Me_3_), 49.1 (CH_2_), 87.0 (Ph-C-O (x2)), 91.0 (C-C=O), 109.6 (C-Bu^t^), 119.1 (CH), 122.3 (CH), 125.2 (2CH), 126.4 (2CH), 126.8 (CH), 128.3 (CH), 128.4 (2CH), 128.5 (2CH), 128.6 (2CH), 134.6 (C), 137.4 (C), 141.3 (C), 149.1 (C) and 172.5 (C=O).

Apart from some recovered diene starting material (0.84 g), a large amount of the oxidised starting diene, 1,2-dibenzoylbenzene (2.39 g, 55%) was isolated, mp 142–146 °C (lit. [26], 146–148 °C); δ_H_ 7.26 (2 H, s), 7.38 (4 H, m), 7.52 (2 H, m), 7.62 (2 H, s) and 7.70 (4 H, m); δ_C_ 128.3 (4CH), 129.6 (2CH), 129.8 (4CH), 130.3 (2CH), 133.0 (2CH), 137.1 (2C), 140.0 (2C) and 196.6 (2C=O) (good agreement with lit. [27]); *m*/*z* (ES) 309.04 (M + Na^+^, 100%).

#### 3.3.8. Preparation of Spiro[2-*t*-Butyl-1,3-dioxolan-4-one-5,5′-1′,2′,3′,4′-tetrachlorocyclohexa-1′,3′-diene] **24**

A solution of **9** (1.53 g, 0.9 mmol) and tetrachlorothiophene 1,1-dioxide **23** (2.49 g, 9 mmol) in CH_2_Cl_2_ (25 cm^3^) was stirred for 11 days. The solvent was then removed and the residual yellow solid recrystallised from CH_2_Cl_2_/hexane. The pure product (1.33 g, 43%) was isolated as pale yellow crystals, mp 86–87 °C; [α]_D_ +8.6 (c = 0.5, CH_2_Cl_2_); (Found: C, 41.9; H, 3.4, C_12_H_24_Cl_4_O_3_ requires C 41.7; H, 3.5%); ν_max_/cm^−1^ 1797, 1611, 1481, 1466, 1260, 1205, 1113, 1110 and 1058; δ_H_ 0.98 (9 H, s, Bu^t^), 2.93 (1 H, d, *J* 18.8, CH_2_), 3.58 (1 H, d, *J* 18.8, CH_2_) and 5.48 (1 H, s, CH-Bu^t^); δ_C_ 23.2 (Bu^t^), 34.6 (C-Me_3_), 42.3 (CH_2_), 110.8 (CH-Bu^t^), 127.4 (2 × C-Cl), 131.2 (2 × C-Cl) and 177.6 (C=O); *m*/*z* (CI+) 345.95 (M^+^, 12.5%) and 260.89 (M^+^–CO–C_4_H_10_, 100).

#### 3.3.9. Pyrolysis of Spiro[2-*t*-Butyl-1,3-dioxolan-4-one-5,5′-1′,2′,3′,4′-tetrachlorocyclohexa-1′,3′-diene] **24**

FVP of the reactant **24** (99.7 mg, 0.28 mmol) was performed at 550 °C and 2.3 × 10^−2^ Torr. The cold trap contained pivalaldehyde and no trace of the expected product tetrachlorophenol was seen. The solid brown/orange product obtained at the furnace exit was 2,3,4,5-tetrachlorobenzoic acid **25** in a quantitative yield (determined by dissolution in acetone and solvent removal). The melting point was unobtainable due to decomposition; ν_max_/cm^−1^ 1797; δ_H_ (CD_3_SOCD_3_) 8.09 (s, CH); δ_C_ (CD_3_SOCD_3_) 129.2 (CH), 130.0 (C), 131.7 (C), 132.6 (C), 133.2 (C), 134.0 (C) and 164.8 (C=O).

### 3.4. Preparation and Reactions of Diels–Alder Adducts of 5-Methylene-2,2-dimethyl-1,3-dioxolan-4-one **26**

#### 3.4.1. Preparation of Spiro[2,2-Dimethyl-1,3-dioxolan-4-one-5,2′-bicyclo[2.2.1]hept-5′-ene] **27**

A solution of **26** (2.47 g, 19 mmol) and freshly distilled cyclopentadiene (1.85 g, 29 mmol) in toluene (75 cm^3^) was heated under reflux for 18 h. The solvent was removed and the crude product was purified by flash column chromatography (SiO_2_, dry loaded, Et_2_O/hexane, 1:4). The product was obtained as a yellow liquid (0.81 g, 22%) which contained some impurities of both starting materials which were probably produced by a retro-Diels–Alder reaction on the column. ν_max_/cm^−1^ 1844, 1785, 1671, 1281, 1174, 1134, 1034, 1080 and 1007; δ_H_ 1.37 (1 H, dd, *J* 12.3, 4.0, H_2_C-7′), 1.46–1.52 (1 H, m, H_2_C-7′), 1.51 (3 H, q, *J* 0.6, CH_3_), 1.59 (3 H, q, *J* 0.6, CH_3_), 2.04–2.08 (1 H, m, H_2_C-3′), 2.32 (1 H, dd, *J* 12.3, 3.6, H_2_C-3′), 2.95–3.00 (1 H, m, HC-1′ or HC-4′), 3.05–3.09 (1 H, m, HC-1′ or HC-4′), 6.17 (1 H, dd, *J* 5.6, 3.0, =CH) and 6.48 (1 H, dd, *J* 5.6, 3.1, =CH); δ_C_ 27.5 (Me) 27.7 (Me), 42.0 (CH), 42.3 (CH_2_), 47.3 (CH_2_), 52.4 (CH), 84.9 (C-5/C-2′), 108.9 (C-2), 133.0 (=CH), 140.3 (=CH) and 176.6 (C=O). *m*/*z* (ES) 217.03 (M + Na^+^, 100%).

#### 3.4.2. Preparation of Spiro[2,2-Dimethyl-1,3-dioxolan-4-one-5,6′-3′-oxatricyclo[3.2.1.0^2,4^]octane] **28**

The alkene **27** (0.80 g, 4.0 mmol) was stirred in CH_2_Cl_2_ (20 cm^3^) for 5 min. Sodium carbonate (5 g) was then added, followed by the addition of peracetic acid (40% in acetic acid, 0.60 g, 8.0 mmol). The reaction proceeded immediately with vigorous bubbling. The mixture was allowed to stir for 6 days and then filtered to remove the sodium salts. The filtrate was evaporated to yield the product as a colourless oil (0.86 g). ^1^H NMR spectroscopic analysis indicated that the product had been formed; however, some starting material and other impurities remained. The crude product was purified by Kugelrohr distillation to furnish the product as a brown oil (0.40 g) in which a small amount of impurities remained including a minor diastereomer (indicated by ^13^C NMR); δ_H_ 1.33–1.40 (1 H, m, H_2_C-8′), 1.48–1.55 (1 H, m, H_2_C-8′ or H_2_C-7′), 1.54 (3 H, s, Me), 1.61 (3 H, s, Me), 1.57–1.60 (1 H, m, H_2_C-8′ or H_2_C-7′), 2.19 (1 H, dd, J 13.2, 4.1, H_2_C-7′), 2.56–2.60 (1 H, m, HC-1′ or HC-5′), 2.72–2.74 (1 H, m, HC-1′ or HC-5′), 3.30 and 3.44 (2 H, AB pattern of d, J 13.6, 3.6, HC-2′ and HC-4′); δ_C_ 23.9 (C-8′ or C-7′), 27.3 (Me), 27.5 (Me), 36.3 (C-1′ or C-5′), 39.3 (C-8′ or C-7′), 46.4 (C-1′ or C-5′), 47.9 (C-2′ or C-4′), 50.9 (C-2′ or C-4′), 84.3 (C-5/C-6′), 109.4 (C-2) and 174.9 (C=O); *m*/*z* (ES) 233.06 (M + Na^+^, 100%); HRMS (CI): found 211.0968. C_11_H_15_O_4_ (M + H) requires 211.0970.

#### 3.4.3. Preparation of Spiro[2,2-Dimethyl-1,3-dioxolan-4-one-5,9′-1′,8′-diphenyl-11′-oxatricyclo[6.2.1.0^2,7^]undeca-2′,4′,6′-triene] **29a** and **29b**

A solution of **26** (1.02 g, 7.9 mmol) and 1,3-diphenylisobenzofuran (2.14 g, 7.9 mmol) in toluene (25 cm^3^) was heated under reflux for 48 h. The reaction mixture was cooled and the solvent removed to yield an orange liquid. The crude mixture was purified by flash column chromatography (SiO_2_, dry loaded, Et_2_O/hexane, 1:2) to give the major product **29a** as pale green crystals (0.13 g, 4%) mp 189–190 °C. A further quantity of this product (0.20 g, 7%) was isolated in further recrystallisations. Later fractions contained the minor product **29b** which was recrystallised to give off-white crystals (0.15 g, 5%) mp 180–181 °C. An amount of mixed product (0.73 g) remained that could potentially be recrystallised to yield further product.

Major product; (5*R*,1′*R*,8′*S*)-*spiro[2,2-dimethyl-1,3-dioxolan-4-one-5,9′-1′,8′-diphenyl-11′-oxatricyclo[6.2.1.0^2,7^]undeca-2′,4′,6′-triene]* **29a** (Found: C, 77.9; H, 5.4. C_26_H_22_O_4_ requires C 78.4; H, 5.6%); ν_max_/cm^−1^ 1783, 1460, 1314, 1294, 1252, 1127, 1008 and 984; δ_H_ 0.98 (3 H, s, Me), 1.46 (3 H, s, Me), 2.79 and 2.91 (2 H, AB pattern, *J* 11.6, H_2_C-10′), 6.92–6.99 (1 H, m), 7.21–7.27 (2 H, m), 7.38–7.55 (7 H, m) and 7.62–7.69 (4 H, m); δ_C_ 25.7 (Me), 28.3 (Me), 51.0 (C-10′), 87.2 (C-1′ or C-8′), 88.7 (C-1′ or C-8′), 94.1 (C-5/C-9′), 109.9 (C-2), 118.3 (CH), 122.9 (CH), 125.6 (2CH), 126.2 (2CH), 126.6 (CH), 128.0 (CH), 128.1 (CH), 128.2 (CH), 128.5 (4CH), 135.0 (C), 137.2 (C) 141.2 (C), 149.2 (C) and 171.8 (C=O); *m*/*z* (ES) 421.20 (M + Na^+^, 100%).

Minor product; (5*R*,1′*S*,8′*R*)*-spiro[2,2-dimethyl-1,3-dioxolan-4-one-5,9′-1′,8′-diphenyl-11′-oxatricyclo[6.2.1.0^2,7^]undeca-2′,4′,6′-triene]* **29b**: ν_max_/cm^−1^ 1787, 1661, 1377, 1276 and 938; δ_H_ 1.59 (3 H, s, CH_3_), 1.69 (3 H, s, CH_3_), 2.41 (1 H, d, *J* 11.7, H_2_C-10′), 3.13 (1 H, d, *J* 11.7, H_2_C-10′) and 7.00–7.86 (14 H, m, Ph); δ_C_ 26.5 (CH_3_), 27.6 (CH_3_), 49.3 (C-10′), 86.5 (C-1′ or C-8′), 88.5 (C-1′ or C-8′), 89.8 (C-5/C-9′), 109.5 (C-2), 119.0 (CH), 121.7(CH), 125.3 (2CH), 126.2 (2CH), 127.0 (CH), 127.7 (CH), 128.3 (4CH), 128.5 (2CH), 134.8 (C), 137.1 (C), 143.0 (C), 149.0 (C) and 172.4 (C=O); *m*/*z* (CI) 421.20 (M + Na^+^, 100%).

#### 3.4.4. FVP of 28 to Give 3-Oxatricyclo[3.2.1.0^2,4^]octan-6-one, **19**

FVP of the reactant **28** (0.1412 g, 0.67 mmol) was performed at 550 °C and 2.4 × 10^−2^ Torr. Purification using preparative TLC (SiO_2_, Et_2_O/hexane, 1:3) gave a brown oil which proved to be a 1:1 mixture of the starting material and **19**; δ_H_ 1.21–1.27 (1 H, br m, H_2_C-8), 1.63–1.74 (1 H, m, H_2_C-8), 1.92 (1 H, dd, *J* 17.7, 4.7, H_2_C-7), 2.11 (1 H, dd, *J* 17.7, 3.9, H_2_C-7), 2.79–2.84 (1 H, m, HC-1), 2.85–2.88 (1 H, m, HC-5), 3.27–3.30 (1 H, m, HC-2) and 3.42–3.46 (1 H, m, HC-4).

### 3.5. X-ray Structure Determination of Adducts

Data were collected on a Bruker SMART diffractometer using graphite monochromated Mo Kα radiation *λ* = 0.71073 Å. The data were deposited at the Cambridge Crystallographic Data Centre and can be obtained free of charge via http://www.ccdc.cam.ac.uk/getstructures (accessed on 28 March 2023). The structure was solved by direct methods and refined by full-matrix least-squares against F^2^ (SHELXL, Version 2018/3 [32]).

#### 3.5.1. (2*S*,5*S*,1′*S*)-2-*t*-butyl-5-(1′-(4-methoxyphenyl)-2′-nitroethyl)-5-phenyl-1,3-dioxolan-4-one **6b**

Crystal data for C_23.75_H_25_NO_6_, *M* = 420.46, colourless prism, crystal dimensions 0.20 × 0.15 × 0.05 mm, orthorhombic, space group P2_1_2_1_2 (No. 18), *a* = 18.4427(17), *b* = 19.4772(17), *c* = 6.5275(6) Å, *V* = 2344.8(4) Å^3^, *Z* = 4, *D*_c_ = 1.191 g cm^−3^, *T* = 93(2) K, *R*1 = 0.0695, *Rw*2 = 0.1475 for 3851 reflections with *I* > 2σ(*I*) and 299 variables. CCDC 2240754.

#### 3.5.2. (2*S*,5*S*,4′*S*)-2-*t*-butyl-5-(2-oxo-tetrahydrofuran-4-yl)-5-phenyl-1,3-dioxolan-4-one **14**

Crystal data for C_17_H_20_O_5_, *M* = 304.33, colourless prism, crystal dimensions 0.12 × 0.06 × 0.02 mm, monoclinic, space group P2_1_ (No. 4), *a* = 10.375(2), *b* = 6.2534(14), *c* = 11.802(3) Å, *β* = 91.135(9)°, *V* = 765.6(3) Å^3^, *Z* = 2, *D*_c_ = 1.320 g cm^−3^, *T* = 93(2) K, *R*1 = 0.0328, *Rw*2 = 0.0800 for 2396 reflections with *I* > 2σ(*I*) and 200 variables. CCDC 2240564.

#### 3.5.3. (2*S*,5*R*,1′*R*,2′*R*,4′*R*,5′*R*)-Spiro[2-*t*-butyl-1,3-dioxolan-4-one-5,6′-3′-ethoxycarbonyl-3′-azatricyclo[3.2.1.0^2,4^]octane **17**

Crystal data for C_16_H_23_NO_5_, *M* = 309.35, colourless platelet, crystal dimensions 0.15 × 0.10 × 0.02 mm, orthorhombic, space group P2_1_2_1_2_1_ (No. 19), *a* = 8.0456(18), *b* = 11.070(2), *c* = 17.625(4) Å, *V* = 1569.7(6) Å^3^, *Z* = 4, *D*_c_ = 1.309 g cm^−3^, *T* = 93(2) K, *R*1 = 0.0362, *Rw*2 = 0.0912 for 1534 reflections with *I* > 2σ(*I*) and 201 variables. CCDC 2240565.

#### 3.5.4. (2*R*,5*R*,1′*R*,2′*R*,4′*R*,5′*R*)-Spiro[2-*t*-butyl-1,3-dioxolan-4-one-5,6′-3′-ethoxycarbonyl-3′-azatricyclo[3.2.1.0^2,4^]octane **20**

Crystal data for C_16_H_23_NO_5_, *M* = 309.35, colourless prism, crystal dimensions 0.30 × 0.30 × 0.15 mm, orthorhombic, space group P2_1_2_1_2_1_ (No. 19), *a* = 6.2494(3), *b* = 14.2020(7), *c* = 18.2439(8) Å, *V* = 1619.22(13) Å^3^, *Z* = 4, *D*_c_ = 1.269 g cm^−3^, *T* = 125(2) K, *R*1 = 0.0530, *Rw*2 = 0.1270 for 1629 reflections with *I* > 2σ (*I*) and 200 variables. CCDC 2240563.

#### 3.5.5. (2*S*,5*R*,1′*R*,8′*S*)-Spiro[2-*t*-butyl-1,3-dioxolan-4-one-5,9′-1′,8′-diphenyl-11′-oxatricyclo[6.2.1.0^2,7^]undeca-2′,4′,6′-triene] **22a**

Crystal data for C_30_H_31_O_4.50_ (C_28_H_26_O_4_ • 0.5 Et_2_O), M = 463.55, colourless prism, crystal dimensions 0.15 × 0.15 × 0.15 mm, monoclinic, space group *C*2 (No. 5), *a* = 16.893(5), *b* = 8.986(4), *c* = 16.665(6) Å, *β* = 93.015(9)°, *V* = 2526.2(16) Å^3^, *Z* = 4, *D*_c_ = 1.219 g cm^−3^, *T* = 93(2) K, *R*1 = 0.0709, *Rw*2 = 0.1840, for 2141 reflections with *I* > 2σ (*I*) and 332 variables. CCDC 2240562.

#### 3.5.6. (5*R*,1′*S*,8′*R*)-Spiro[2,2-dimethyl-1,3-dioxolan-4-one-5,9′-1′,8′-diphenyl-11′-oxatricyclo[6.2.1.0^2,7^]undeca-2′,4′,6′-triene] **29b**

Crystal data for C_26_H_22_O_4_, *M* = 398.44, colourless prism, crystal dimensions 0.25 × 0.20 × 0.03 mm, monoclinic, space group P2_1_/c (No. 14), *a* = 10.850(5), *b* = 10.546(4), *c* = 17.931(8) Å, *β* = 102.202(11)°, *V* = 2005.5(15) Å^3^, *Z* = 4, *D*_c_ = 1.320 g cm^−3^, *T* = 93(2) K, *R*1 = 0.1799, *Rw*2 = 0.5039 for 2788 reflections with *I* > 2σ (*I*) and 273 variables. CCDC 2240561.

## 4. Conclusions

The conjugate addition chemistry of the anion from dioxolanone **5** has been extended with confirmation of the absolute stereochemistry of two adducts by X-ray diffraction. Removal of the dioxolanone using flash vacuum pyrolysis gives the 4-benzoylbutyrolactone in good yield and moderate e.e., thus illustrating the use of **5** as a chiral benzoyl anion equivalent. In a sequence of cycloaddition, epoxidation and pyrolytic dioxolanone removal, the methylenedioxolanone **9** acts as a chiral ketene equivalent giving the product **19** in low yield but high e.e. The pyrolytic reactivity of adducts **17** and **24** has given valuable mechanistic information on the thermal fragmentation of 1,3-dioxolan-4-ones. Cycloaddition of methylenedioxolanone **9** with sterically hindered 1,3-dienes generally proceeds in low yield but with good stereoselectivity and the structures of such adducts have been confirmed by X-ray diffraction.

## Data Availability

Not applicable.

## Supplementary Materials

The following are available online at https://www.mdpi.com/article/10.3390/molecules28093845/s1, Figures S1–S49: NMR spectra of new compounds.

## Sample Availability

Samples of the compounds are not available from the authors.
