# Supplementary material for: New Chemistry of Chiral 1,3-Dioxolan-4-Ones
Source: Molecules. 2023 May 1;28(9):3845. doi: 10.3390/molecules28093845 (PMC10180285; doi:10.3390/molecules28093845)

New Chemistry of Chiral 1,3-Dioxolan-4-ones

R. Alan Aitken*, Lynn A. Power and Alexandra M. Z. Slawin
EaStCHEM School of Chemistry, University of St Andrews, North Haugh, St Andrews, Fife, KY16 9ST, UK.

Supplementary Material	Figure
^{1}H NMR, DEPTQ ^{13}C NMR, IR and HRMS spectra of **14**	S1, S2, S3, S4
^{1}H NMR and DEPTQ ^{13}C NMR spectra of **6a**	S5, S6
^{1}H NMR, DEPTQ ^{13}C NMR, IR and HRMS spectra of **6b**	S7, S8, S9, S10
^{1}H NMR, DEPTQ ^{13}C NMR and IR spectra of **7a**	S11, S12, S13
^{1}H NMR, DEPTQ ^{13}C NMR and IR spectra of **7b**	S14, S15, S16
^{1}H NMR and DEPTQ ^{13}C NMR spectra of **16**	S17, S18
^{1}H NMR, DEPTQ ^{13}C NMR and IR spectra of **17**	S19, S20, S21
IR and HRMS spectra of **18**	S22, S23
^{1}H NMR spectrum of **19**	S24
^{1}H NMR, DEPTQ ^{13}C NMR and HRMS spectra of **20**	S25, S26, S27
^{1}H NMR, DEPTQ ^{13}C NMR, IR and HRMS spectra of **21**	S28, S29, S30, S31
^{1}H NMR, DEPTQ ^{13}C NMR and IR spectra of **22a**	S32, S33, S34
^{1}H NMR, DEPTQ ^{13}C NMR and IR spectra of **24**	S35, S36, S37
^{1}H NMR and DEPTQ ^{13}C NMR spectra of **25**	S38, S39
^{1}H NMR and DEPTQ ^{13}C NMR spectra of **27**	S40, S41
^{1}H NMR, DEPTQ ^{13}C NMR and HRMS spectra of **28**	S42, S43, S44
^{1}H NMR, ^{13}C NMR and IR spectra of **29a**	S45, S46, S47
^{1}H NMR and IR spectra of **29b**	S48, S49

Figure S1. 300 MHz DEPTQ ^{1}H NMR spectrum of **14**

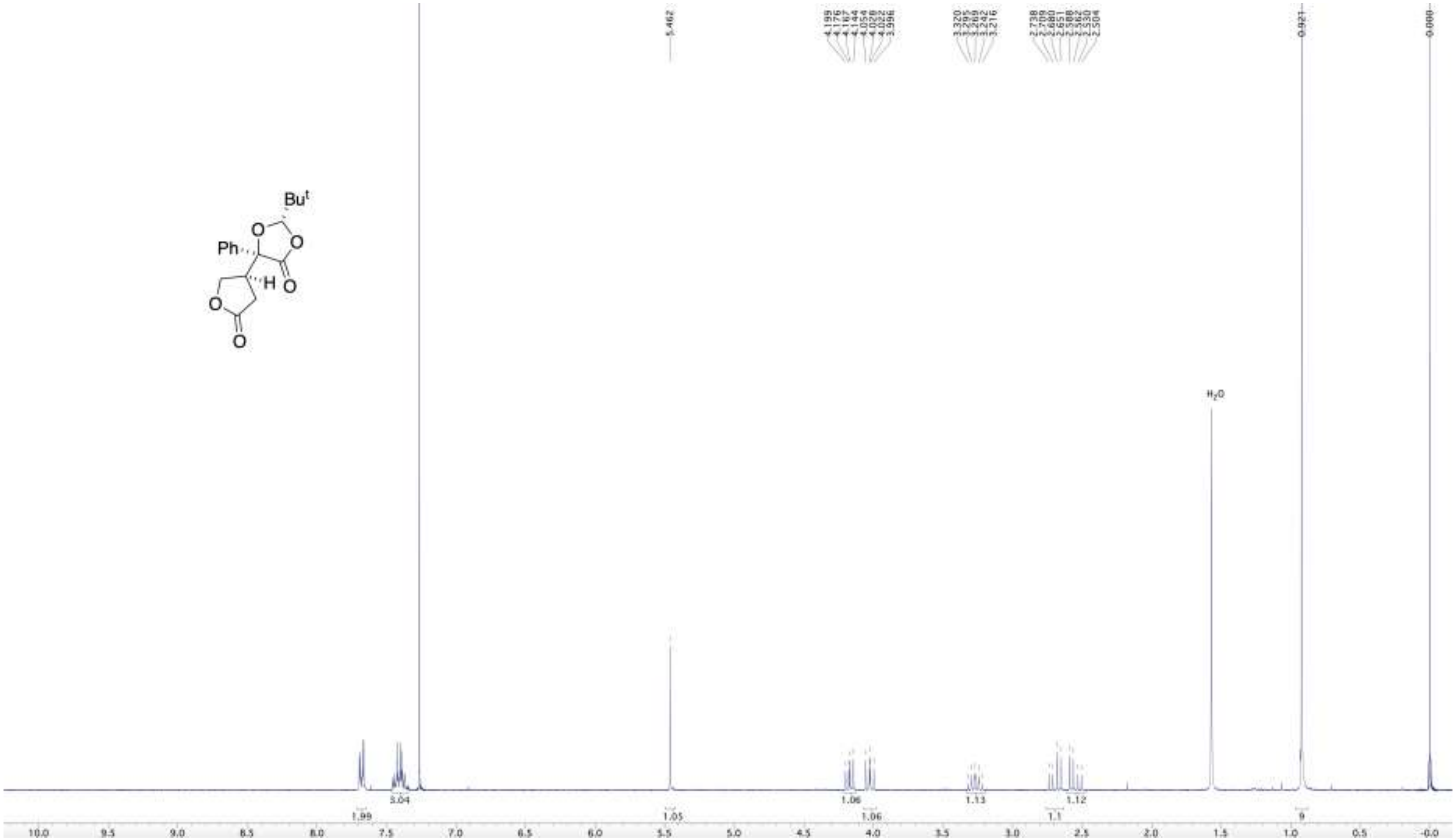

Figure S2. 75 MHz DEPTQ ^{13}C NMR spectrum of **14**

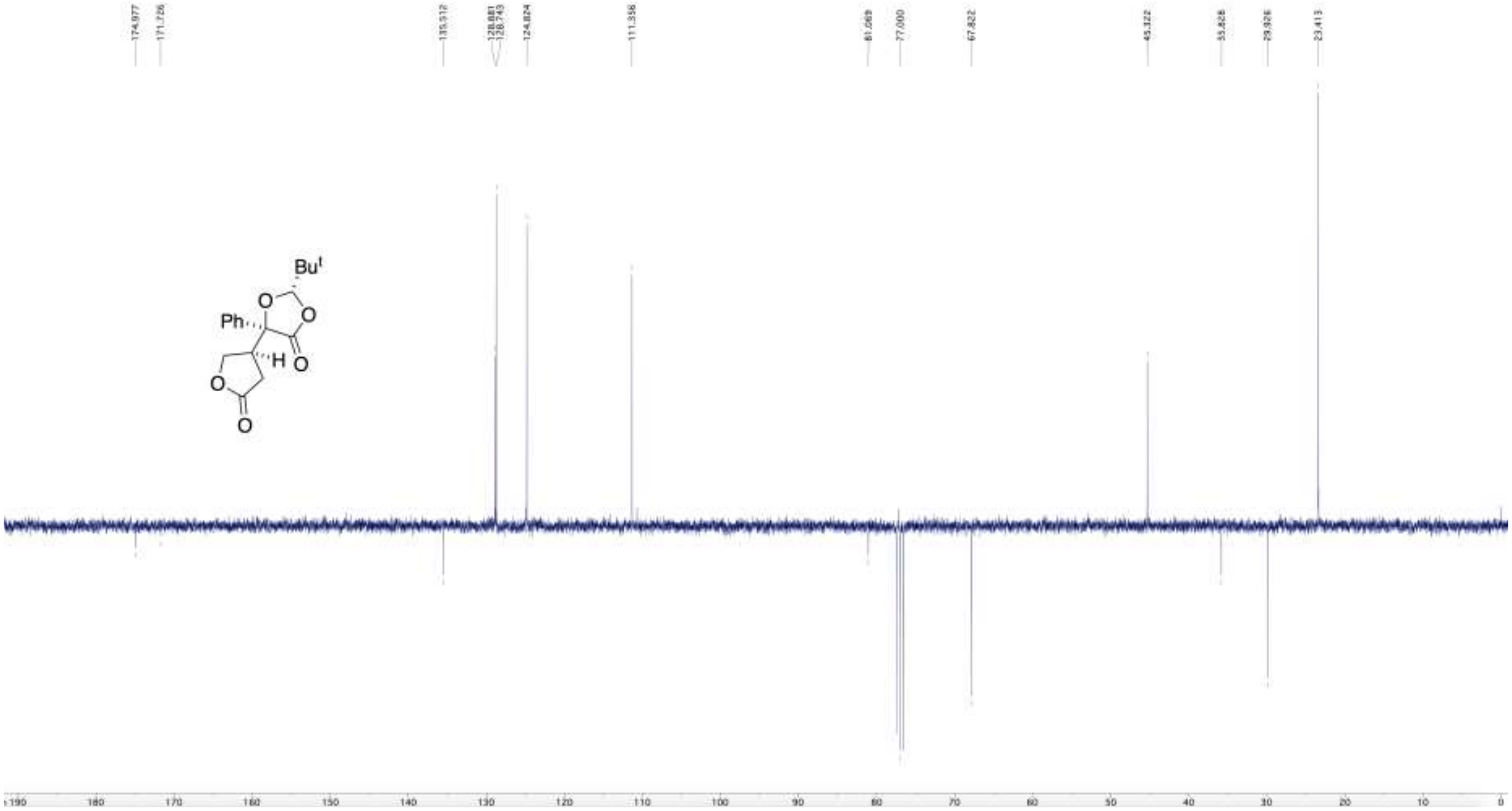

Figure S3. IR spectrum of **14**

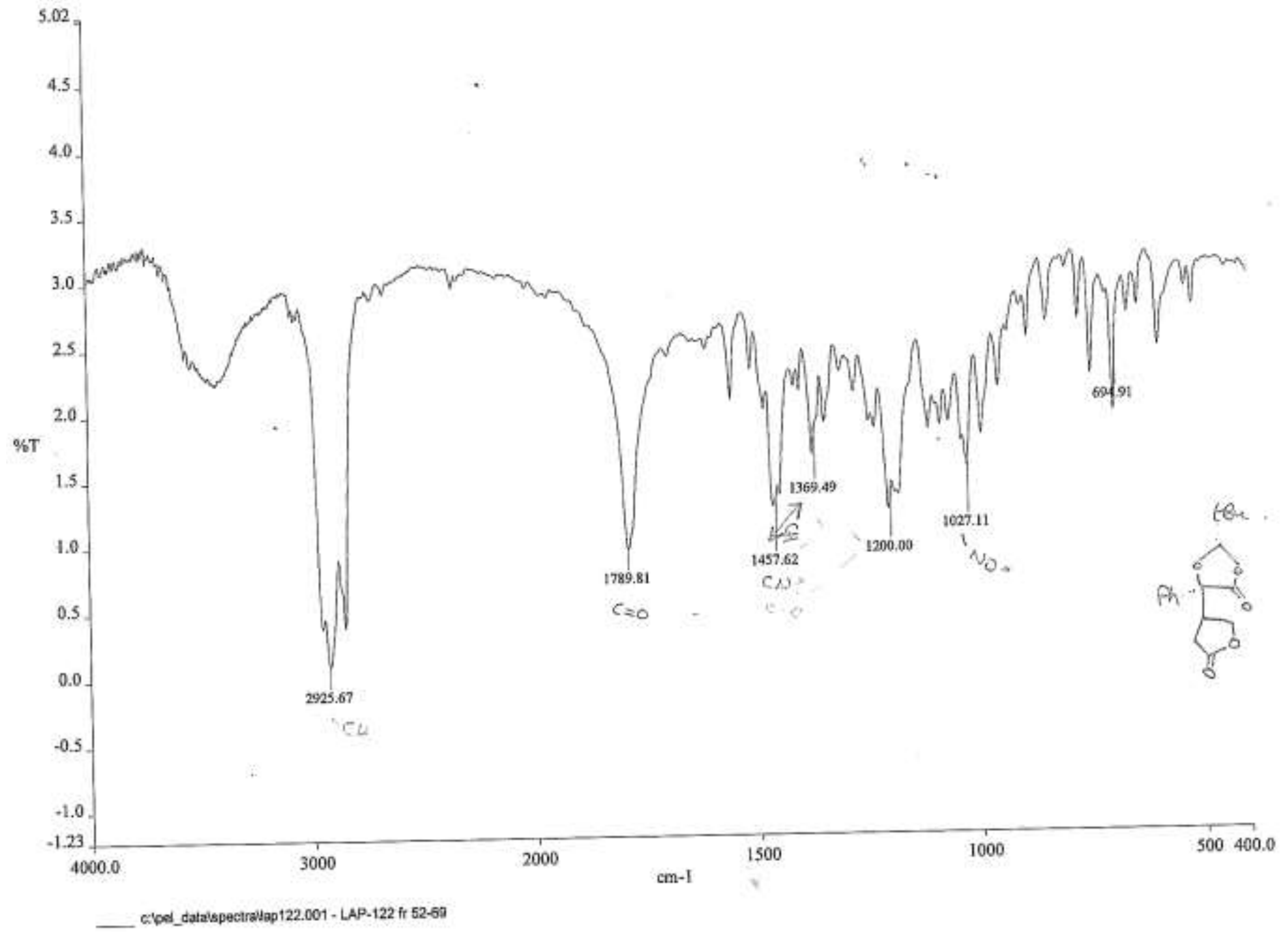

Figure S4. HRMS of **14**

Elemental Composition Report

Single Mass Analysis

Tolerance = 20.0 PPM / DBE: min = -1.5, max = 40.0
Isotope cluster parameters: Separation = 1.0 Abundance = 1.0%

Monoisotopic Mass, Odd and Even Electron Ions
26 formula(e) evaluated with 2 results within limits (up to 8 closest results for each mass)

Lynn Power University of St-Andrews School of Chemistry LCTOF 12-Sep-2007 15:03:51

LAP_122am2 25 (0.486) AM (Cen,2, 80.00, Ar,7000.0,325.19,0.76,LS 5); Cm (25:31) 1: TOF MS ES+ 2.09e4

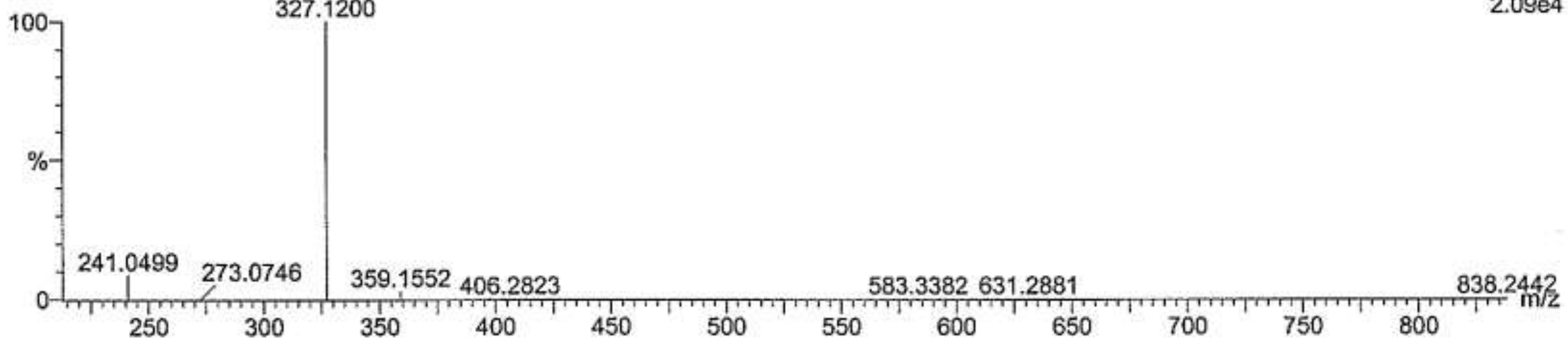

Minimum:				-1.5		
Maximum:		200.0	20.0	40.0		
Mass	Calc. Mass	mDa	PPM	DBE	Score	Formula
327.1200	327.1208	-0.8	-2.5	7.5	1	C17 H20 O5 Na
	327.1232	-3.2	-9.9	10.5	2	C19 H19 O5

Figure S5. 300 MHz ^1H NMR spectrum of **6a**

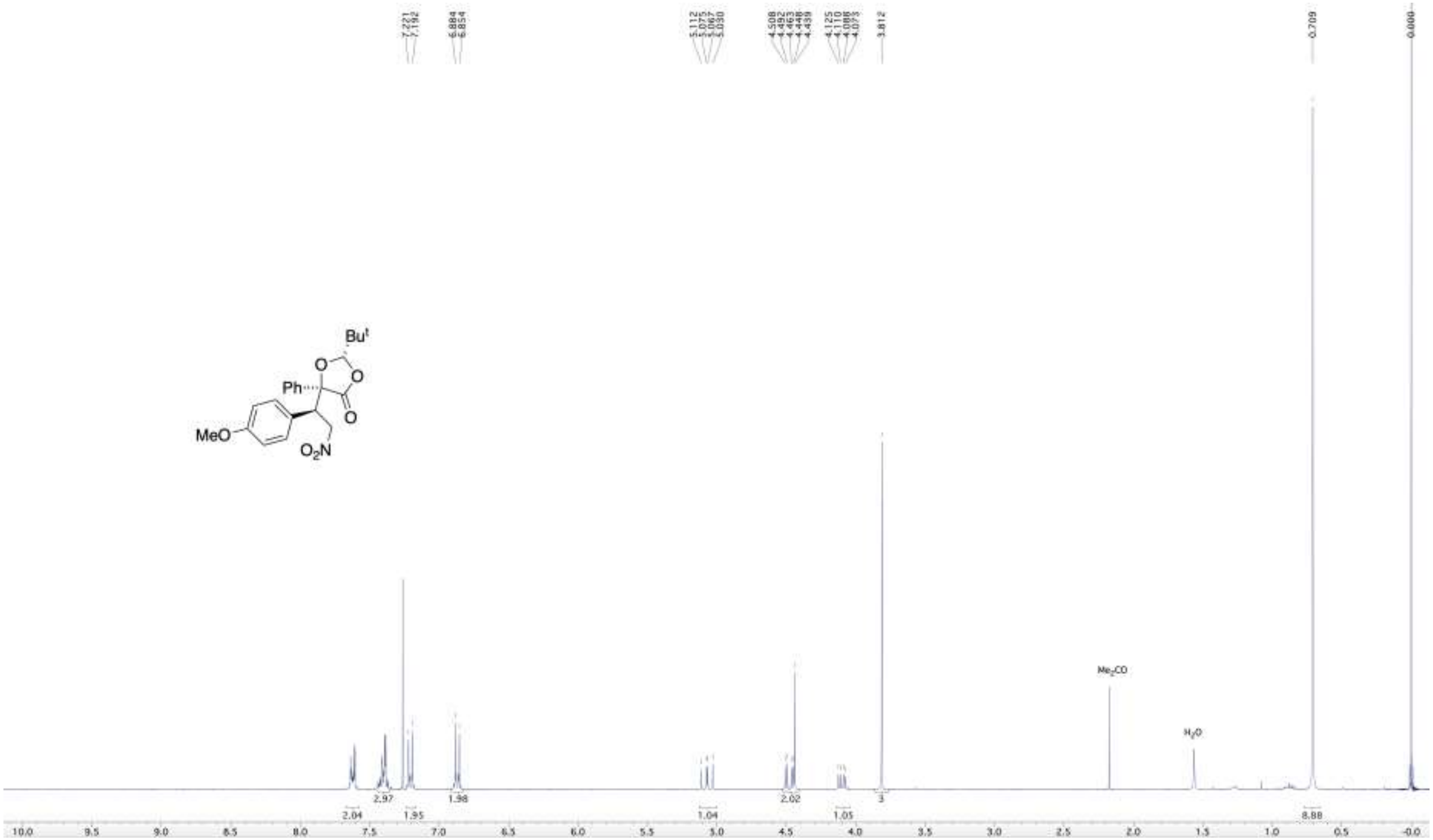

Figure S6. 75 MHz DEPTQ ^{13}C NMR spectrum of **6a**

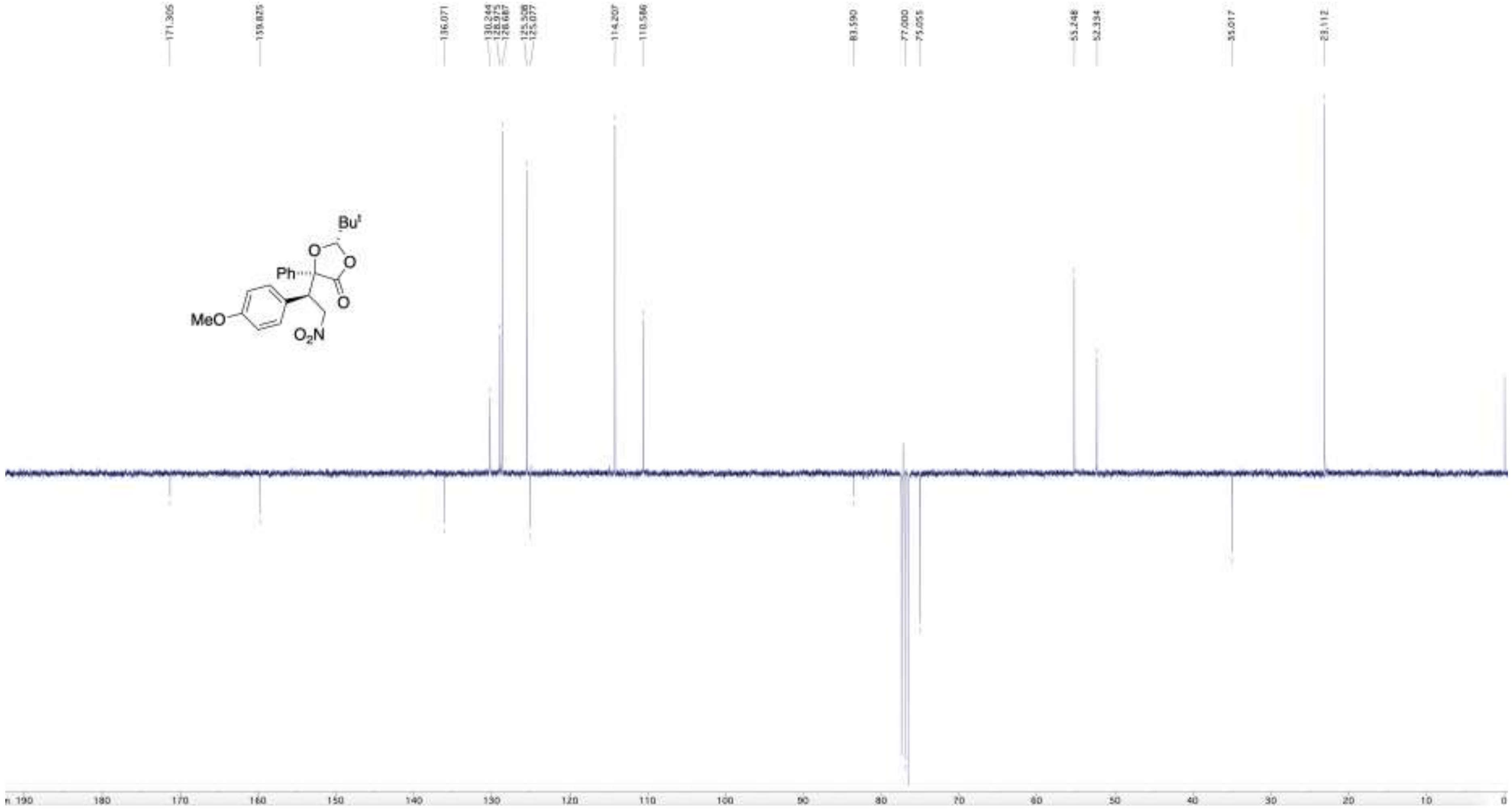

Figure S7. 300 MHz ^{1}H NMR spectrum of **6b**

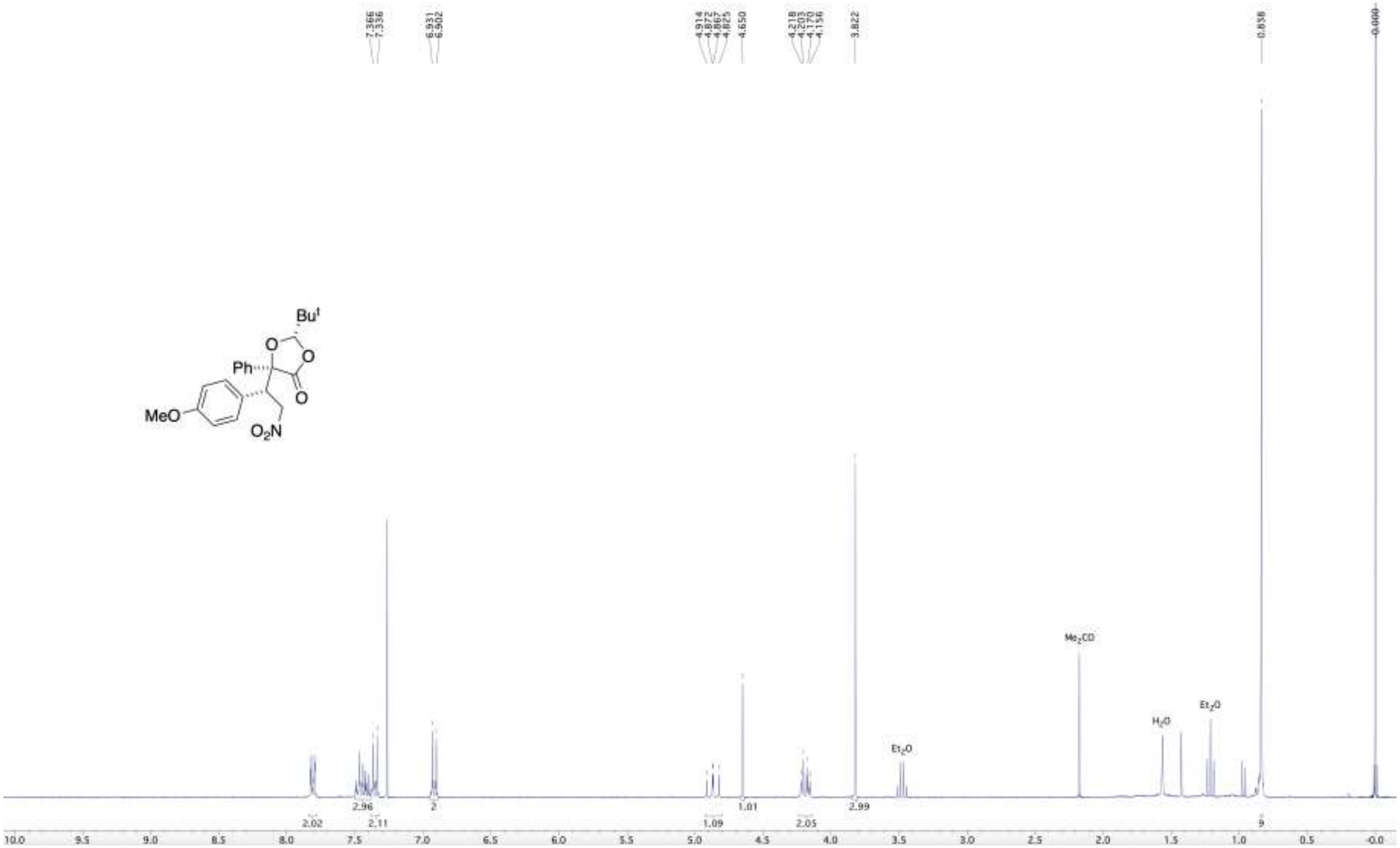

Figure S8. 75 MHz DEPTQ ^{13}C NMR spectrum of **6b**

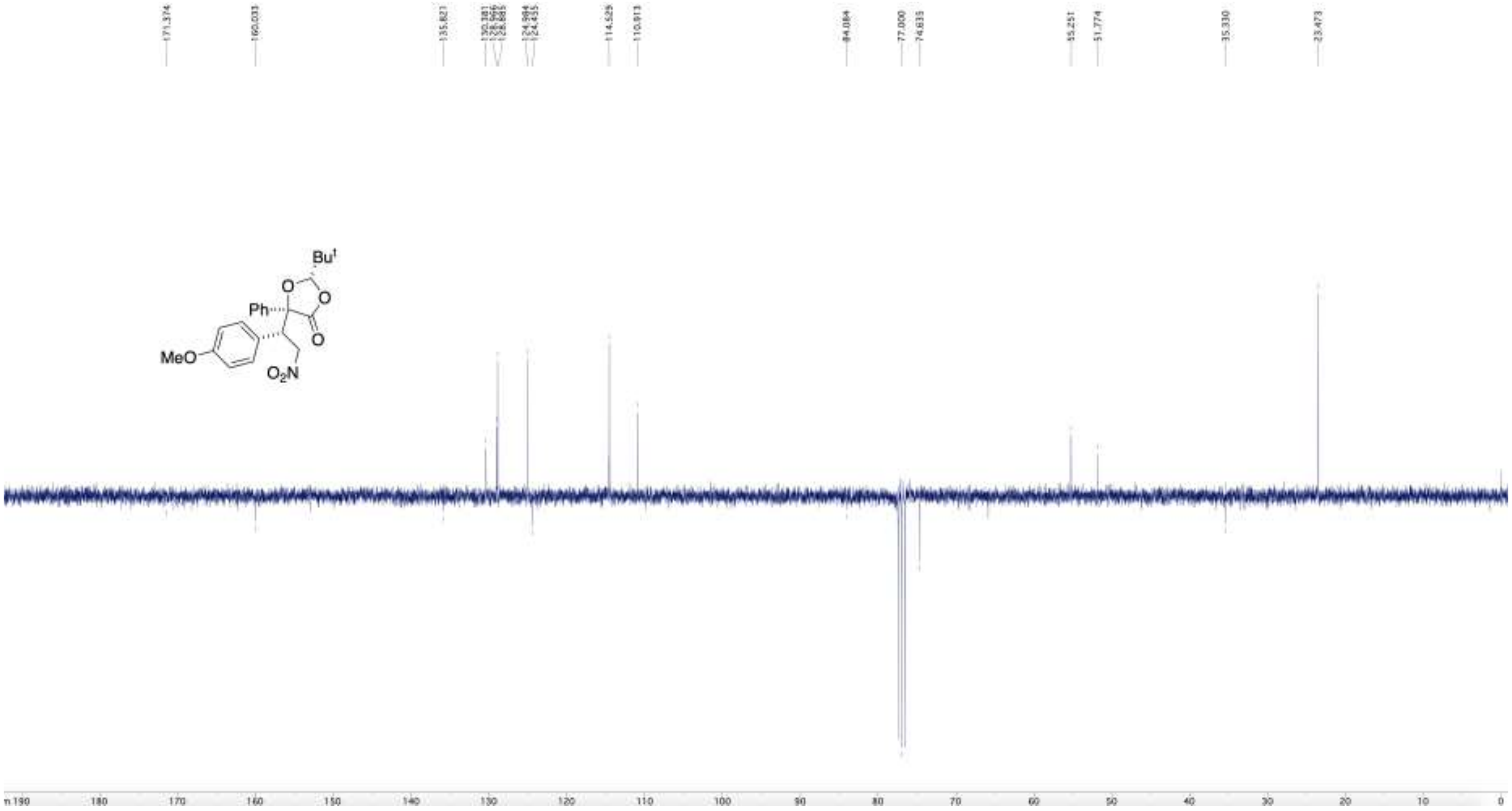

Figure S9. IR spectrum of **6b**

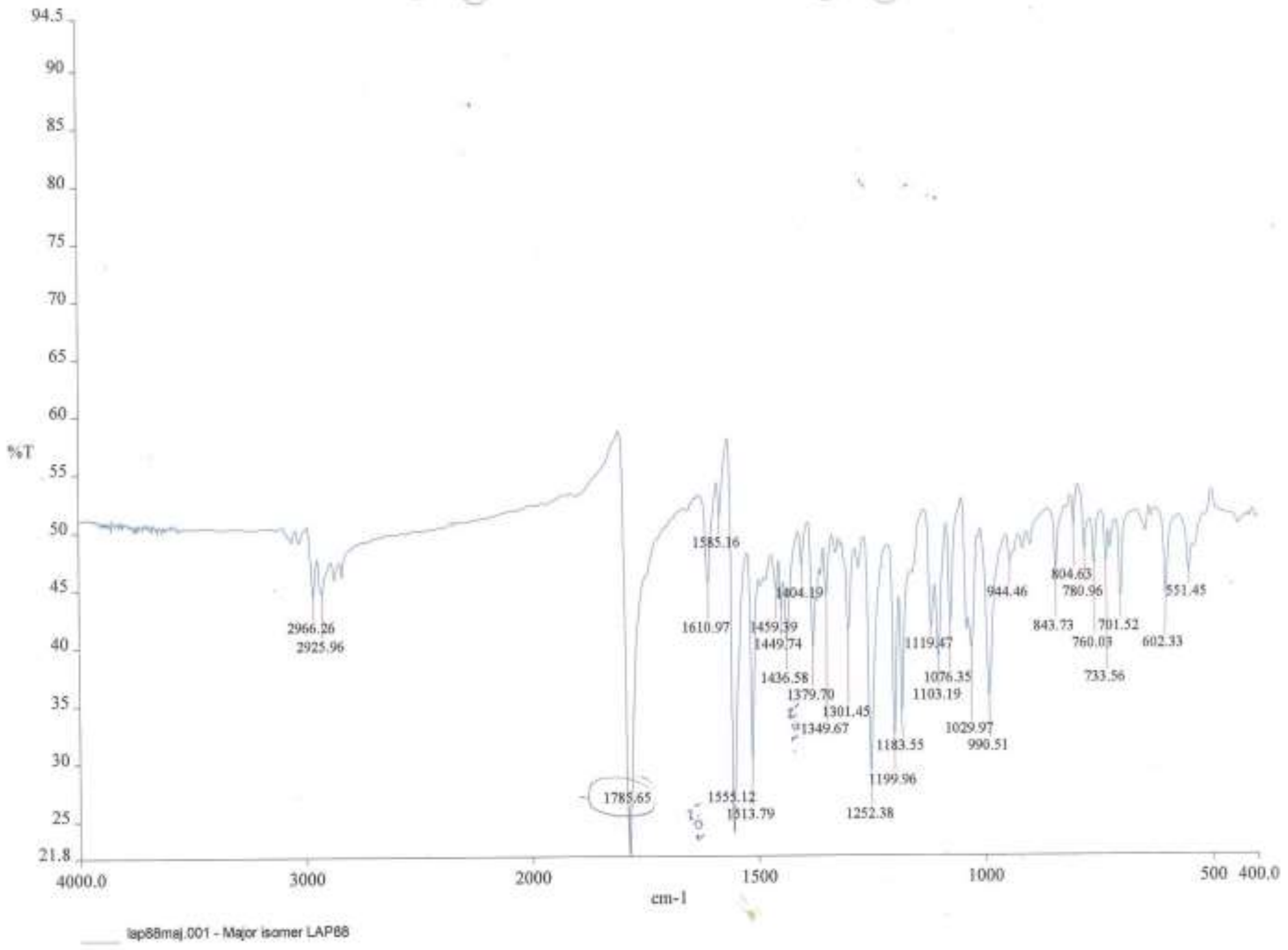

Figure S10. HRMS of **6b**

Elemental Composition Report

Single Mass Analysis

Tolerance = 30.0 PPM / DBE: min = -1.5, max = 40.0
Isotope cluster parameters: Separation = 1.0 Abundance = 1.0%

Monoisotopic Mass, Odd and Even Electron Ions
166 formula(e) evaluated with 19 results within limits (up to 8 closest results for each mass)

Lynn Power
University of St-Andrews
School of Chemistry LCTOF
10-Nov-2005 11:33:16

LAP25fr19_21am 18 (0.352) AM (Cen,2, 80.00, Ar,8000.0,325.19,0.76,LS 5); Cm (15:21-2:7)
1: TOF MS ES+
1.64e4

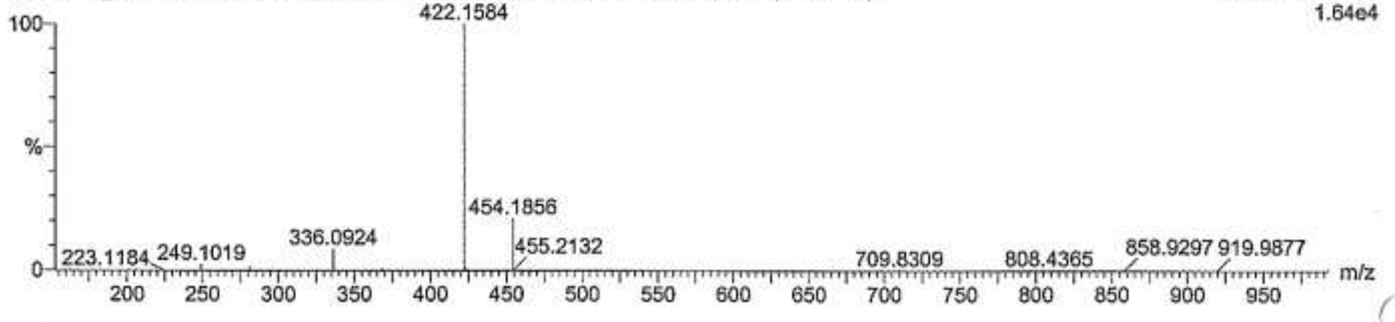

Mass	Calc. Mass	mDa	PPM	DBE	Score	Formula
Minimum:				-1.5		
Maximum:		200.0	30.0	40.0		
422.1584	422.1580	0.5	1.2	10.5	3	C22 H25 N O6 Na
	422.1604	-1.9	-4.5	13.5	4	C24 H24 N O6
	422.1563	2.1	5.0	9.5	2	C19 H24 N3 O8
	422.1606	-2.2	-5.2	15.0	5	C25 H23 N2 O3 Na
	422.1539	4.5	10.7	6.5	1	C17 H25 N3 O8 Na
	422.1630	-4.6	-10.9	18.0	6	C27 H22 N2 O3
	422.1633	-4.9	-11.5	19.5	7	C28 H21 N3 Na
	422.1647	-6.2	-14.7	19.0	8	C30 H23 O Na

Figure S11. 300 MHz ^{1}H NMR spectrum of **7a**

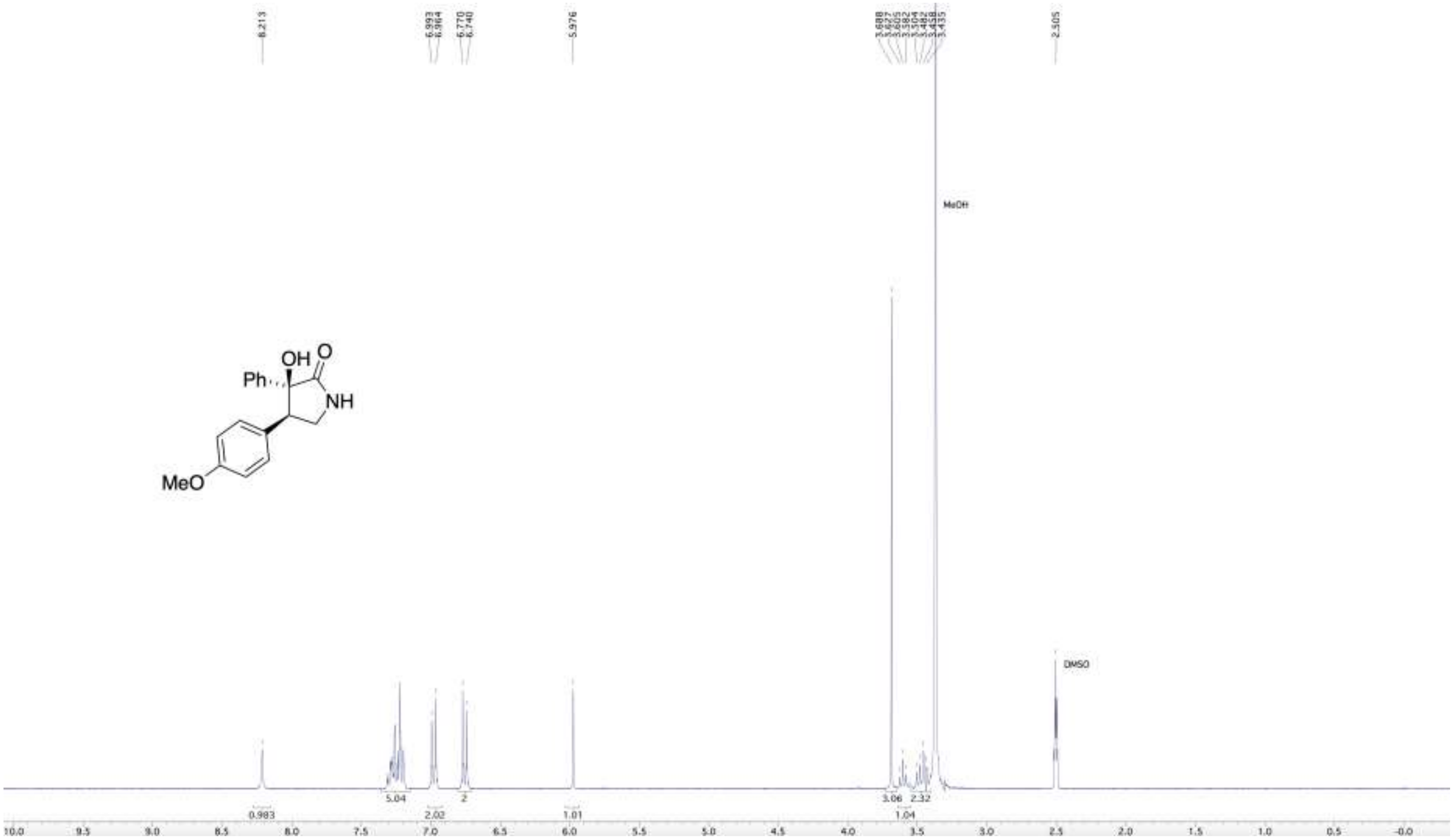

Figure S12. 75 MHz DEPTQ ^{13}C NMR spectrum of **7a**

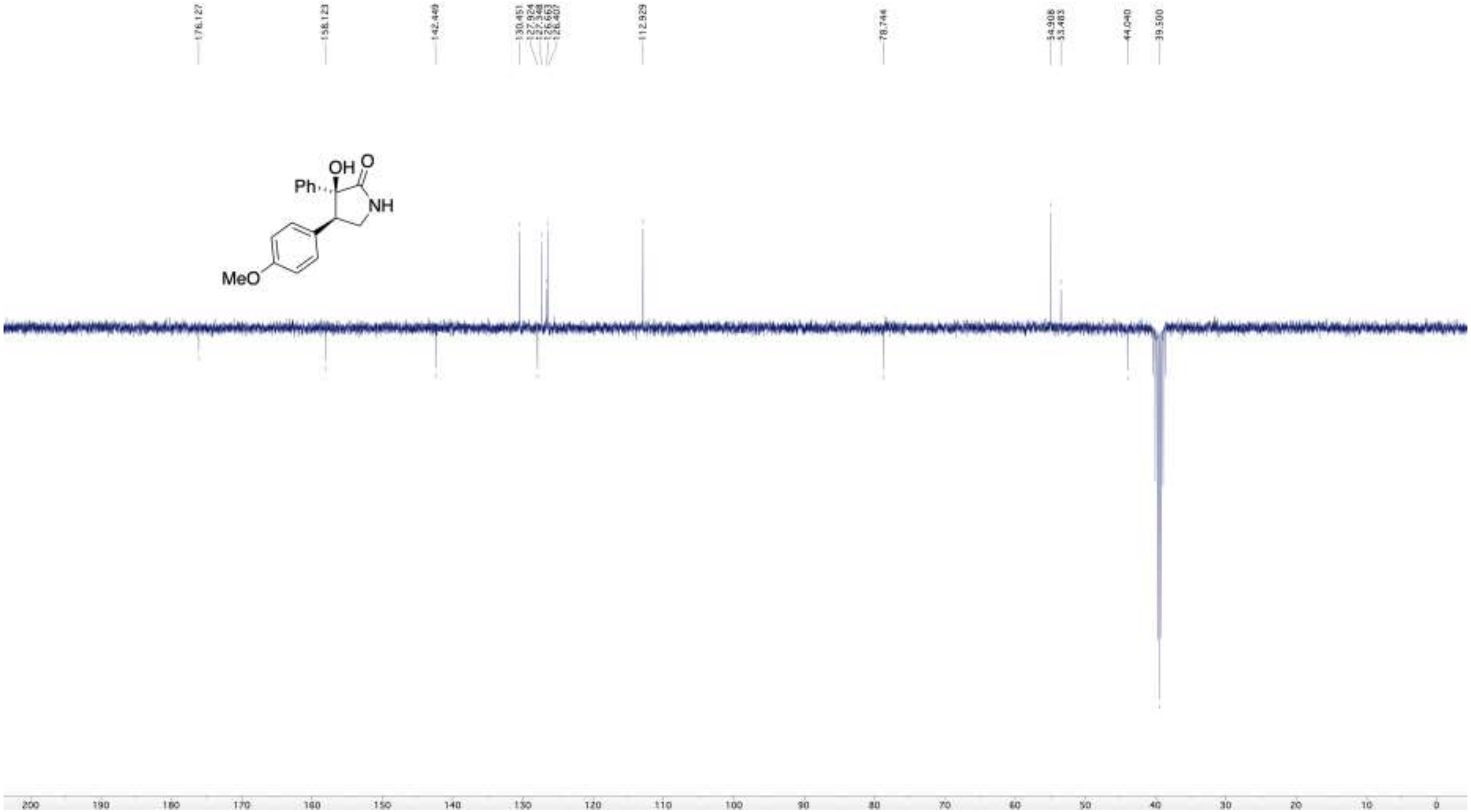

Figure S13. IR spectrum of **7a**

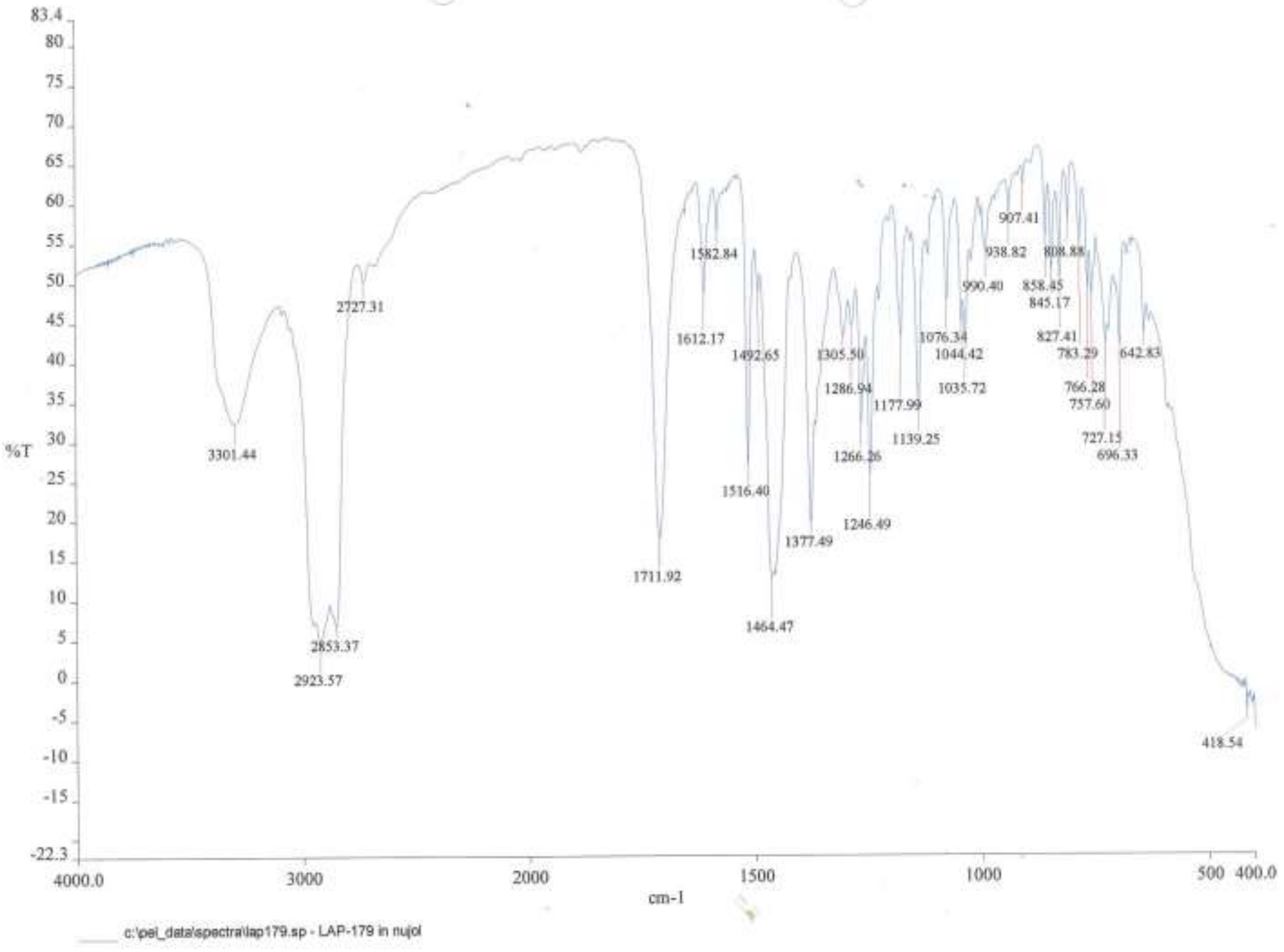

Figure S14. 300 MHz ^{1}H NMR spectrum of **7b**

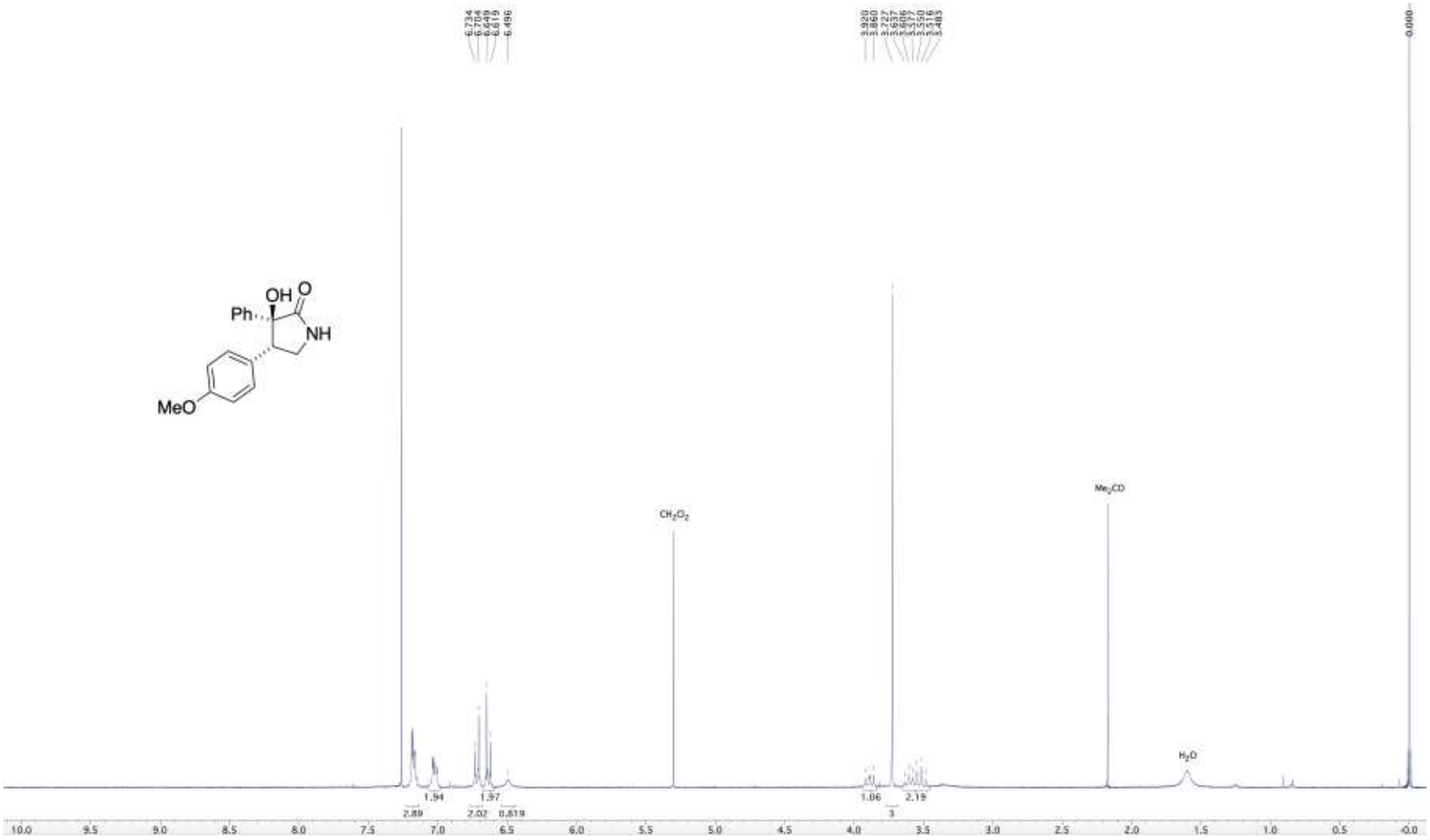

Figure S15. 75 MHz DEPTQ ^{13}C NMR spectrum of **7b**

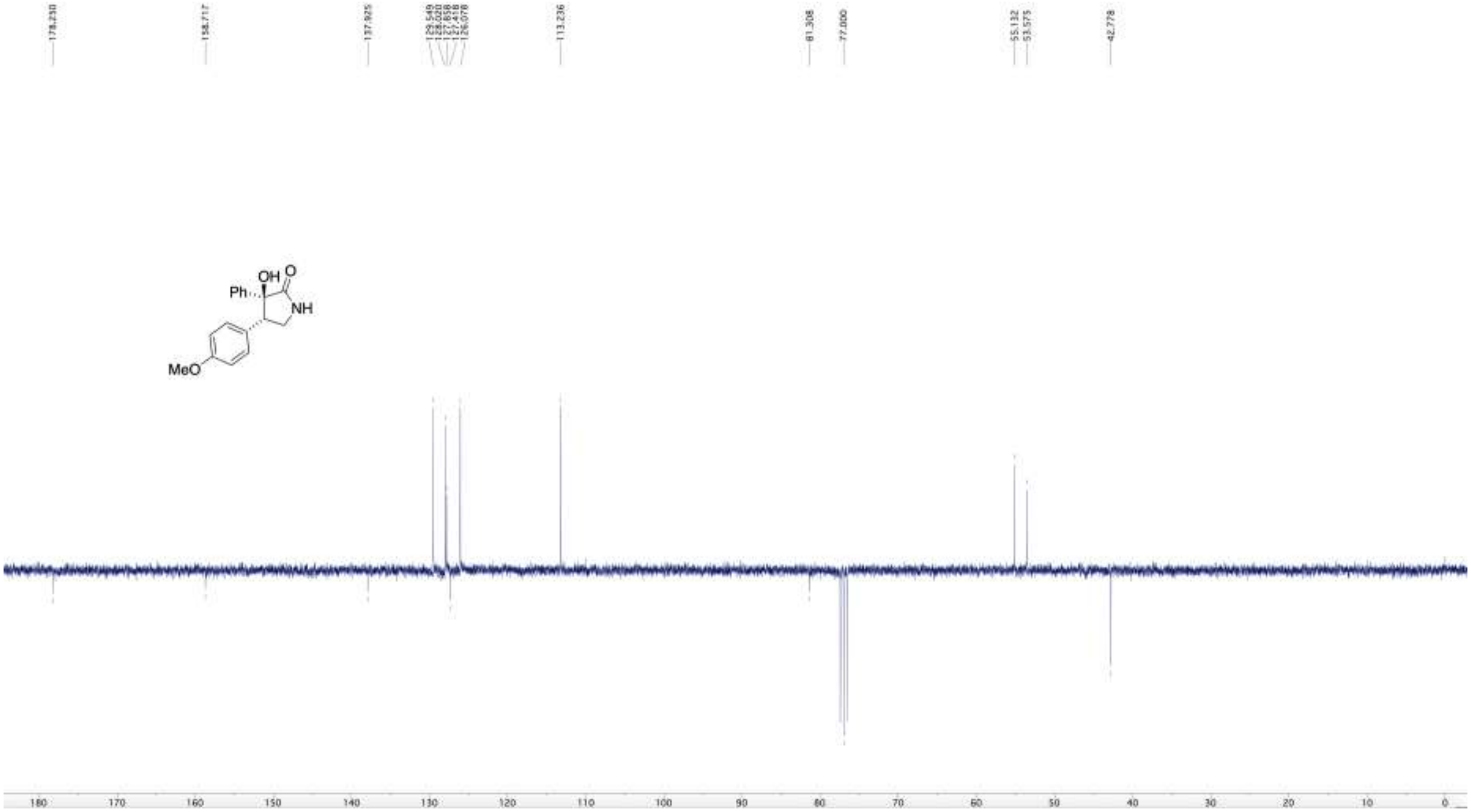

Figure S16. IR spectrum of **7b**

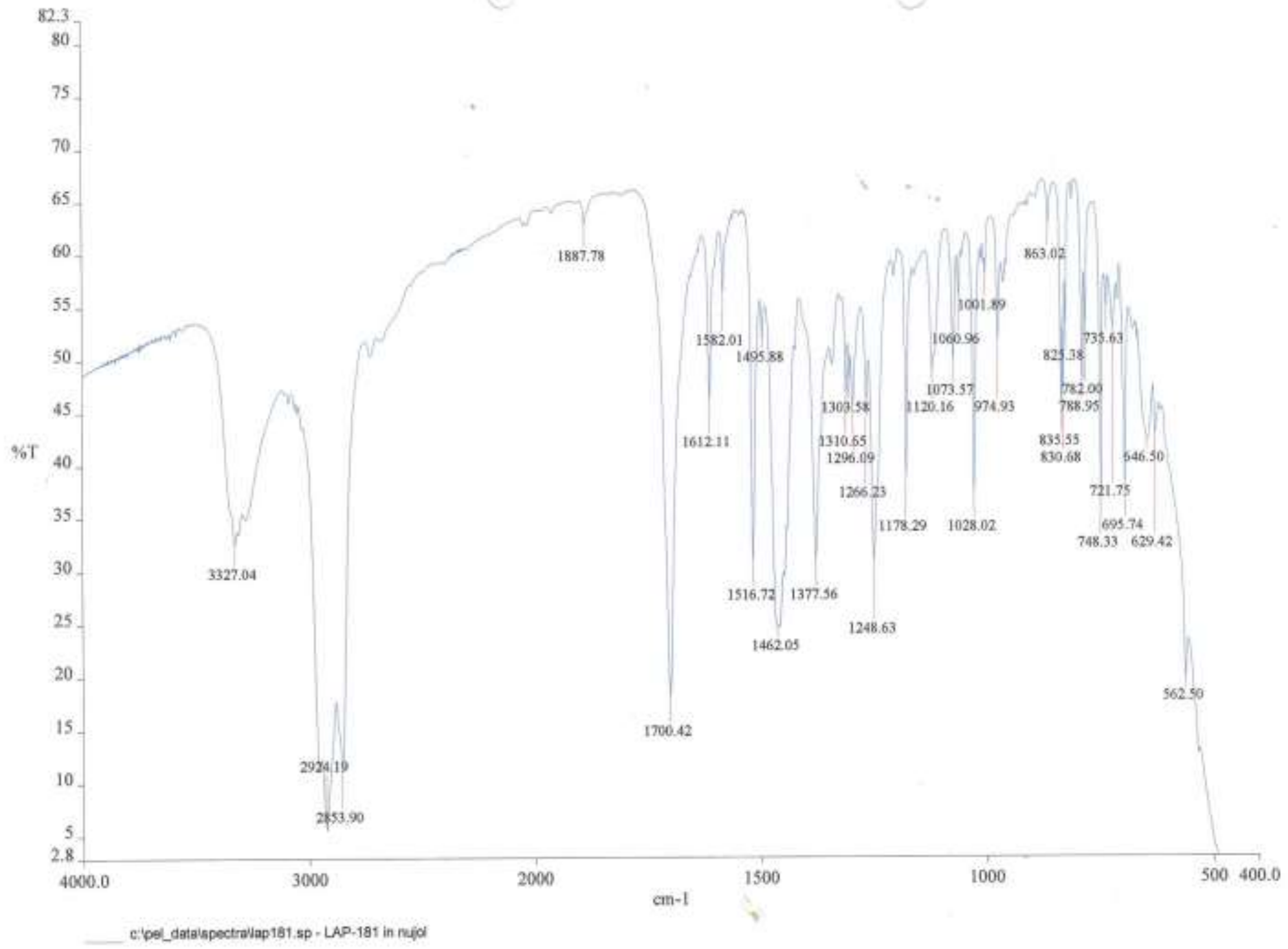

Figure S17. 300 MHz ^{1}H NMR spectrum of **16**

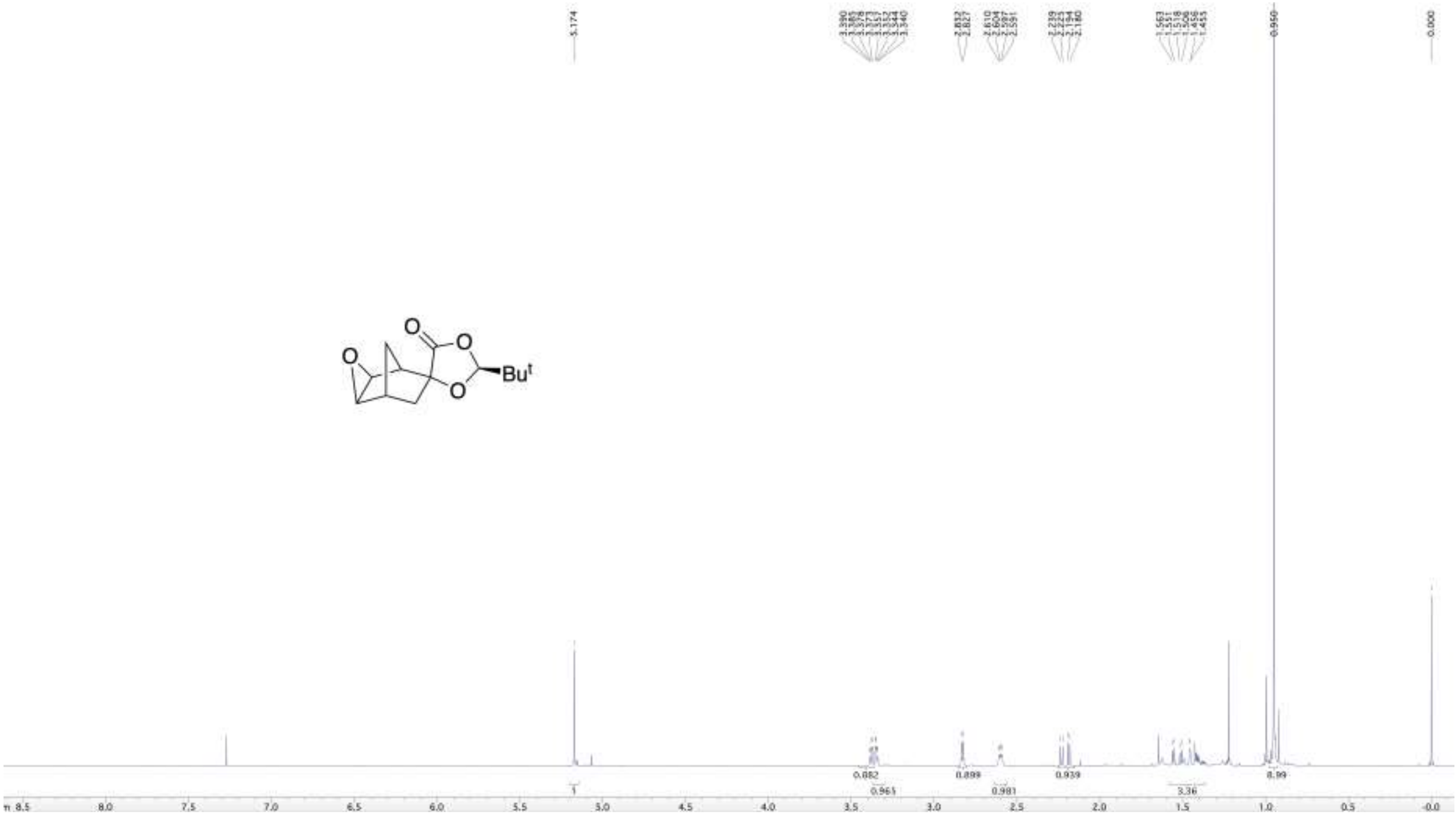

Figure S18. 75 MHz DEPTQ ^{13}C NMR spectrum of **16**

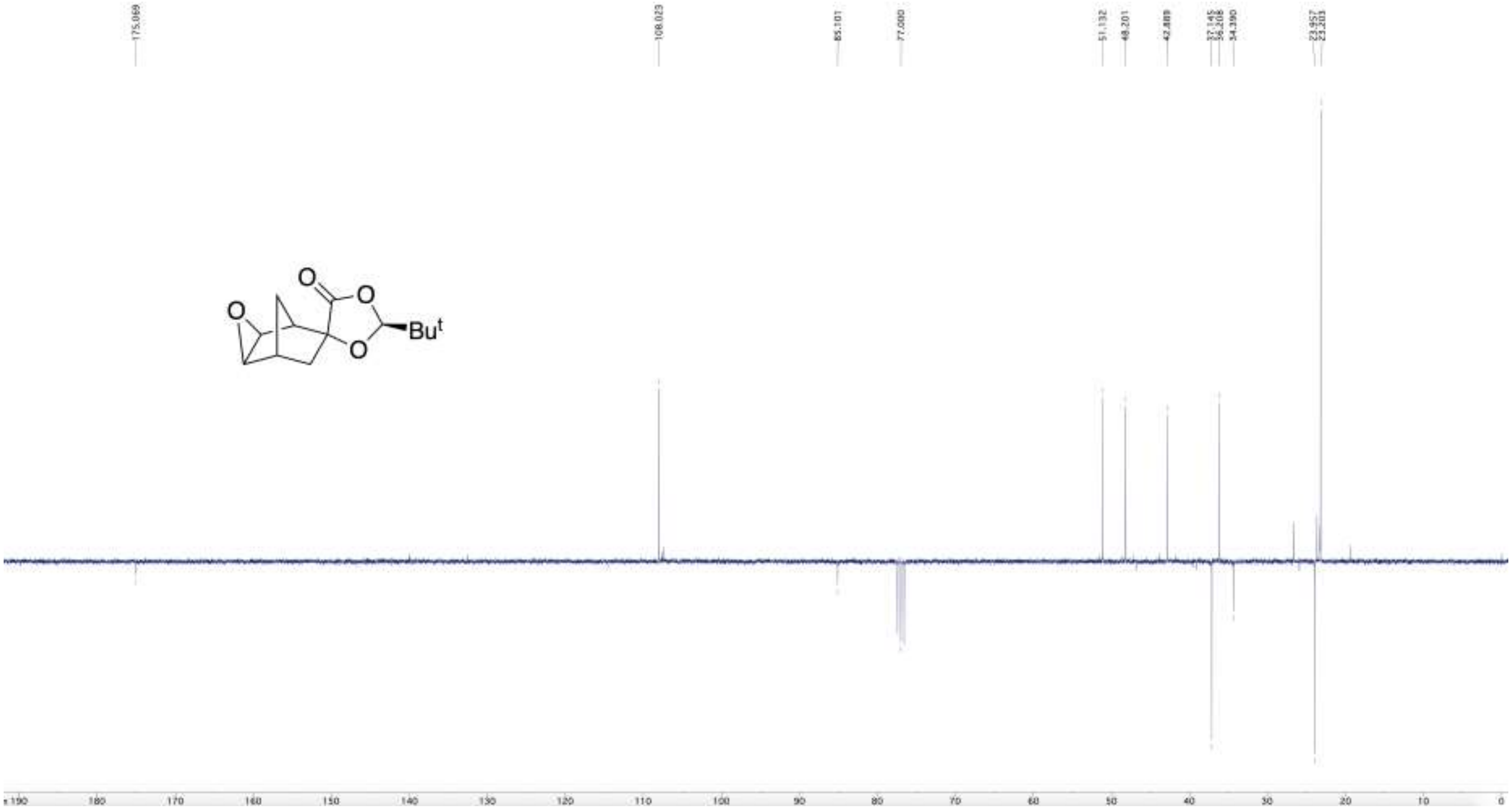

Figure S19. 300 MHz ^{1}H NMR spectrum of **17**

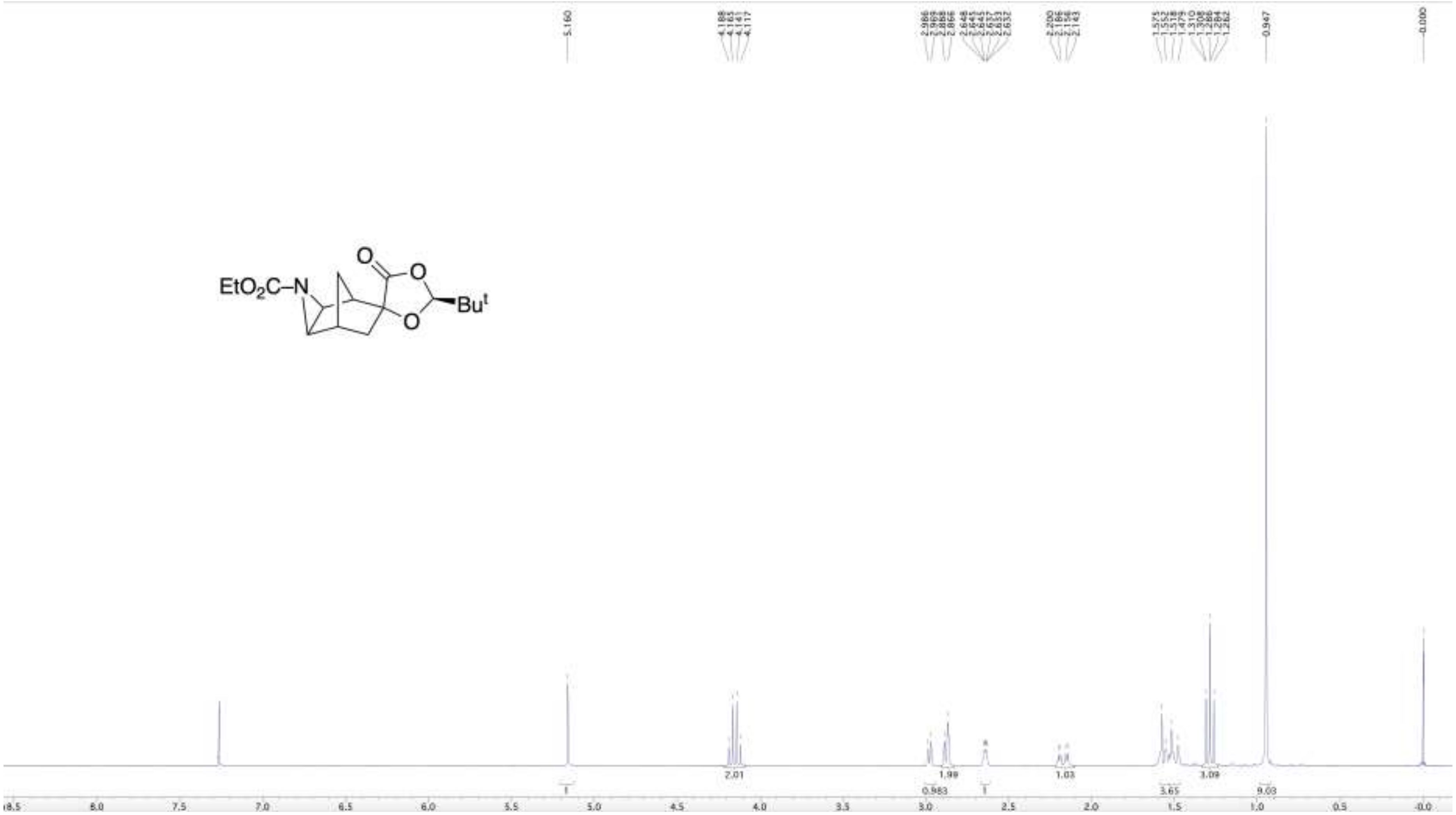

Figure S20. 75 MHz DEPTQ ^{13}C NMR spectrum of **17**

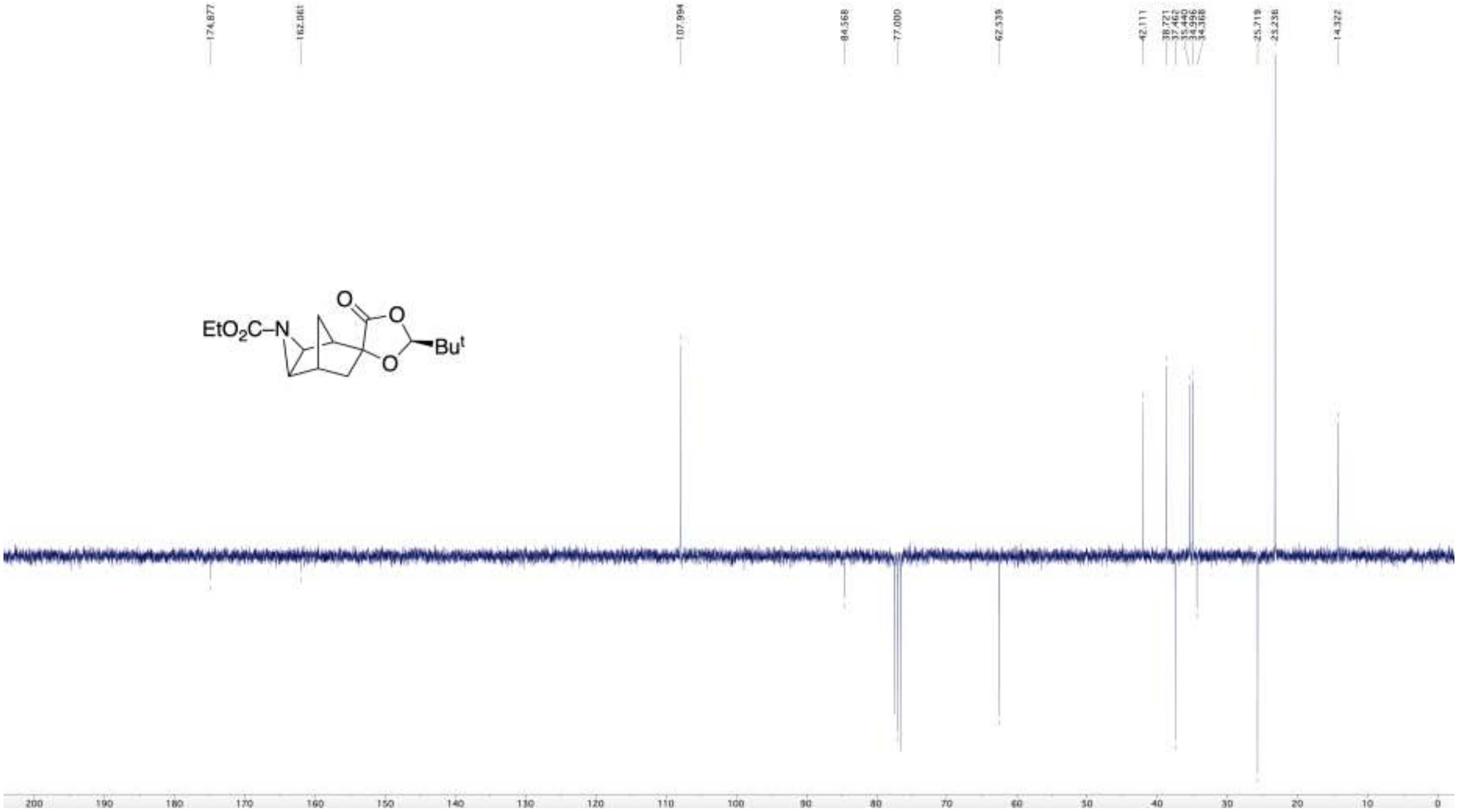

Figure S21. IR spectrum of **17**

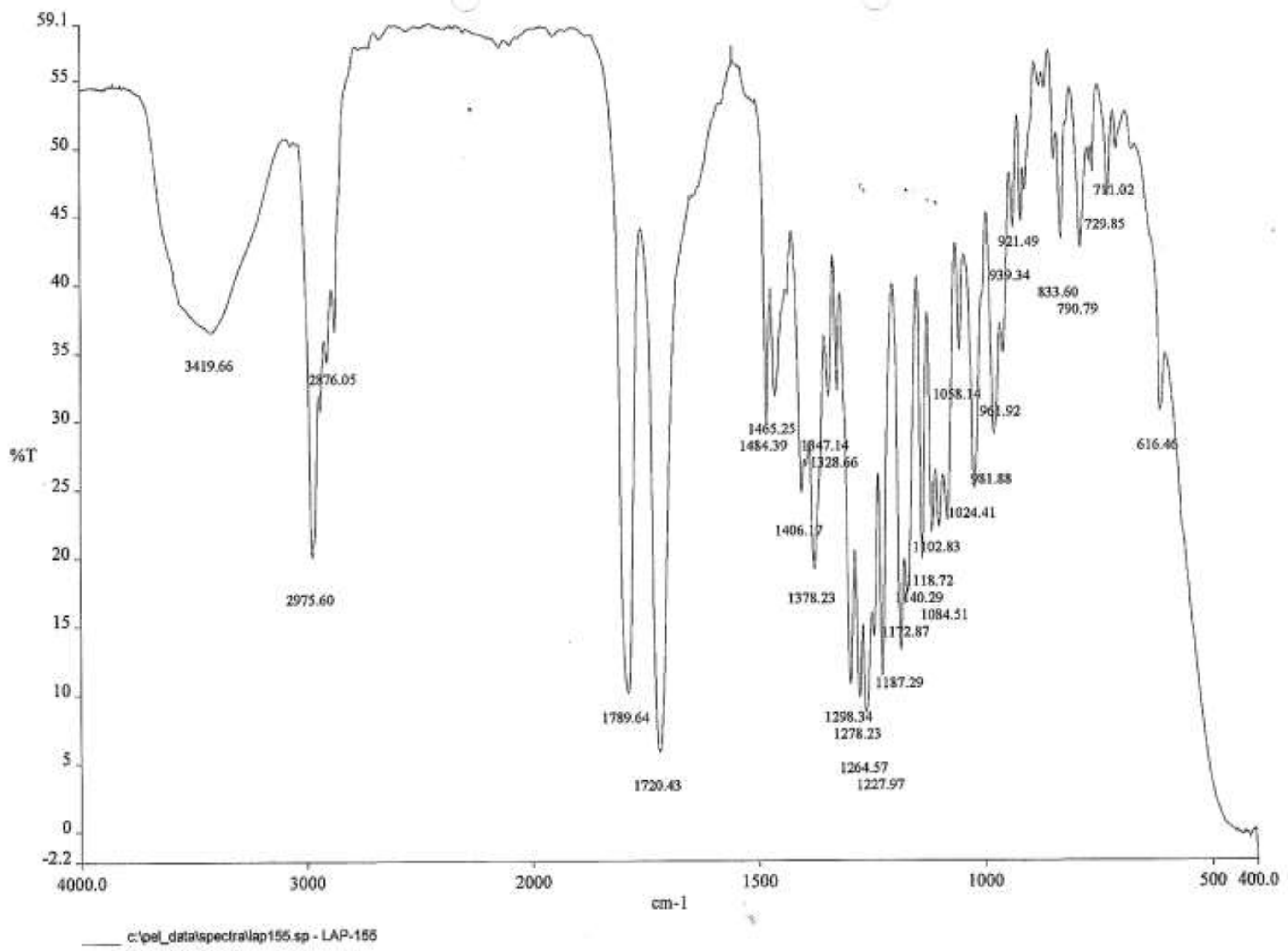

Figure S22. IR spectrum of **18**

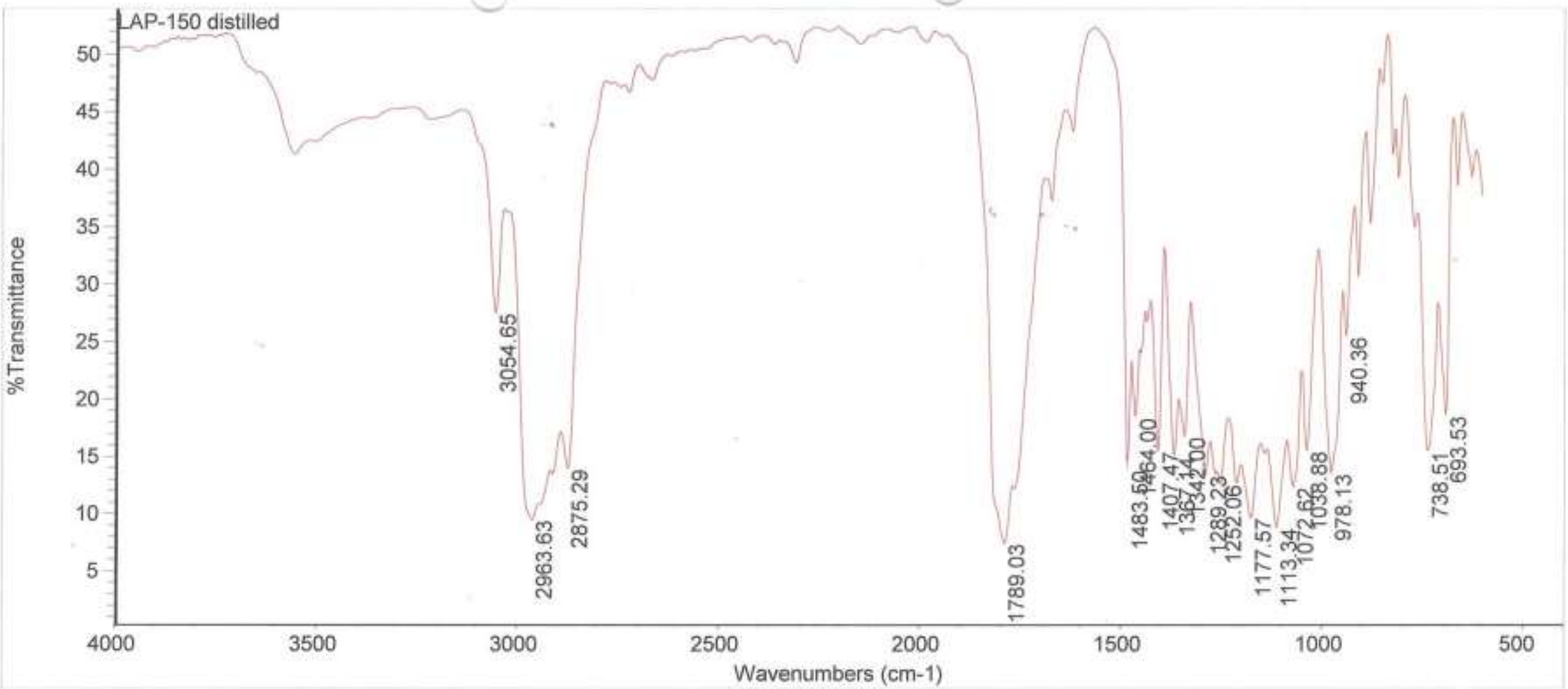

Fri Jul 06 13:59:47 2007 (GMT+01:00)
FIND PEAKS:
Spectrum: LAP-150 distilled
Region: 4000.00 400.00
Absolute threshold: 29.882

Figure S23. HRMS of **18**

Elemental Composition Report

Single Mass Analysis

Tolerance = 50.0 PPM / DBE: min = -1.5, max = 40.0

Isotope cluster parameters: Separation = 1.0 Abundance = 1.0%

Monoisotopic Mass, Odd and Even Electron Ions

18 formula(e) evaluated with 1 results within limits (up to 8 closest results for each mass)

Lynn Power

University of St-Andrews

School of Chemistry GCTOF

09-Jul-2007 11:44:41

LAP_150__ci 379 (6.317) Cm (368:382-1:32)

TOF MS CI+

5.20e5

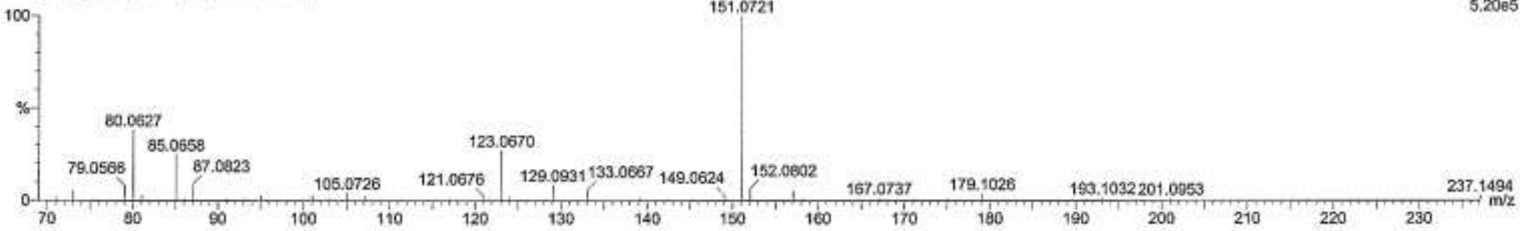

Minimum:				-1.5		
Maximum:		200.0	50.0	40.0		
Mass	Calc. Mass	mDa	PPM	DBE	Score	Formula
237.1494	237.1491	0.3	1.4	4.5	1	C14 H21 O3 + H

Figure S24. 300 MHz ^{1}H NMR spectrum of **19**

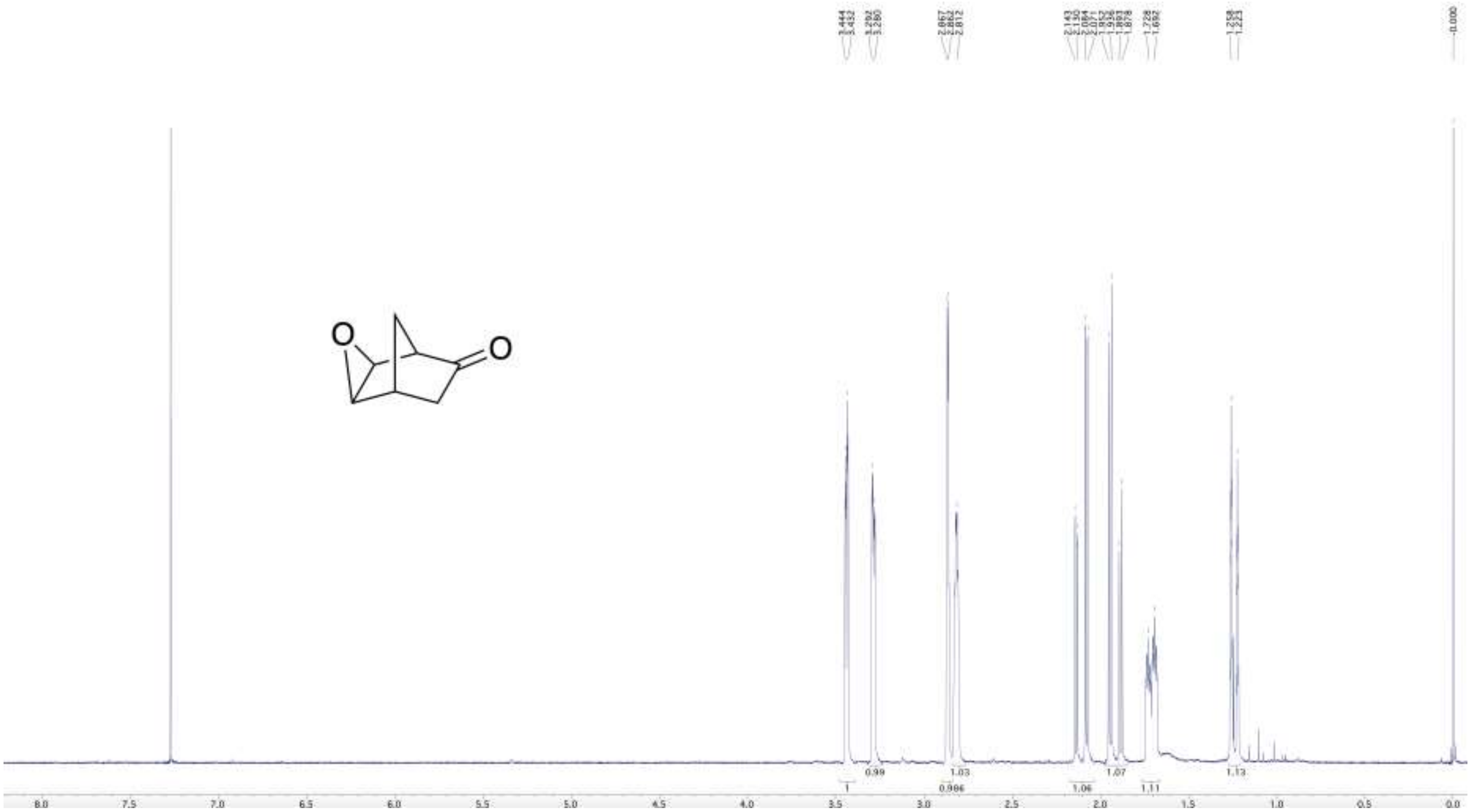

Figure S25. 300 MHz ^{1}H NMR spectrum of **20**

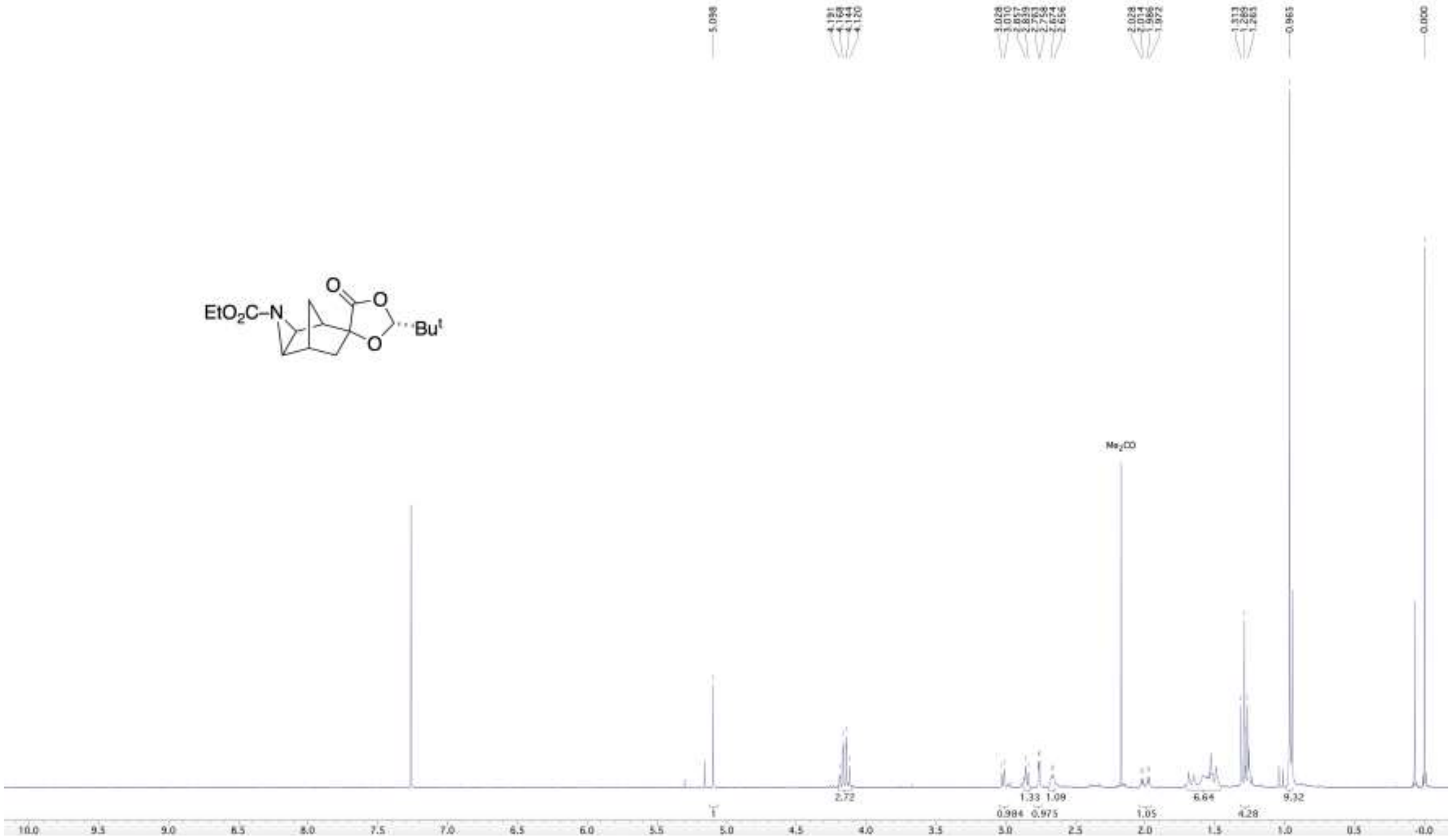

Figure S26. 75 MHz DEPTQ ^{13}C NMR spectrum of **20**

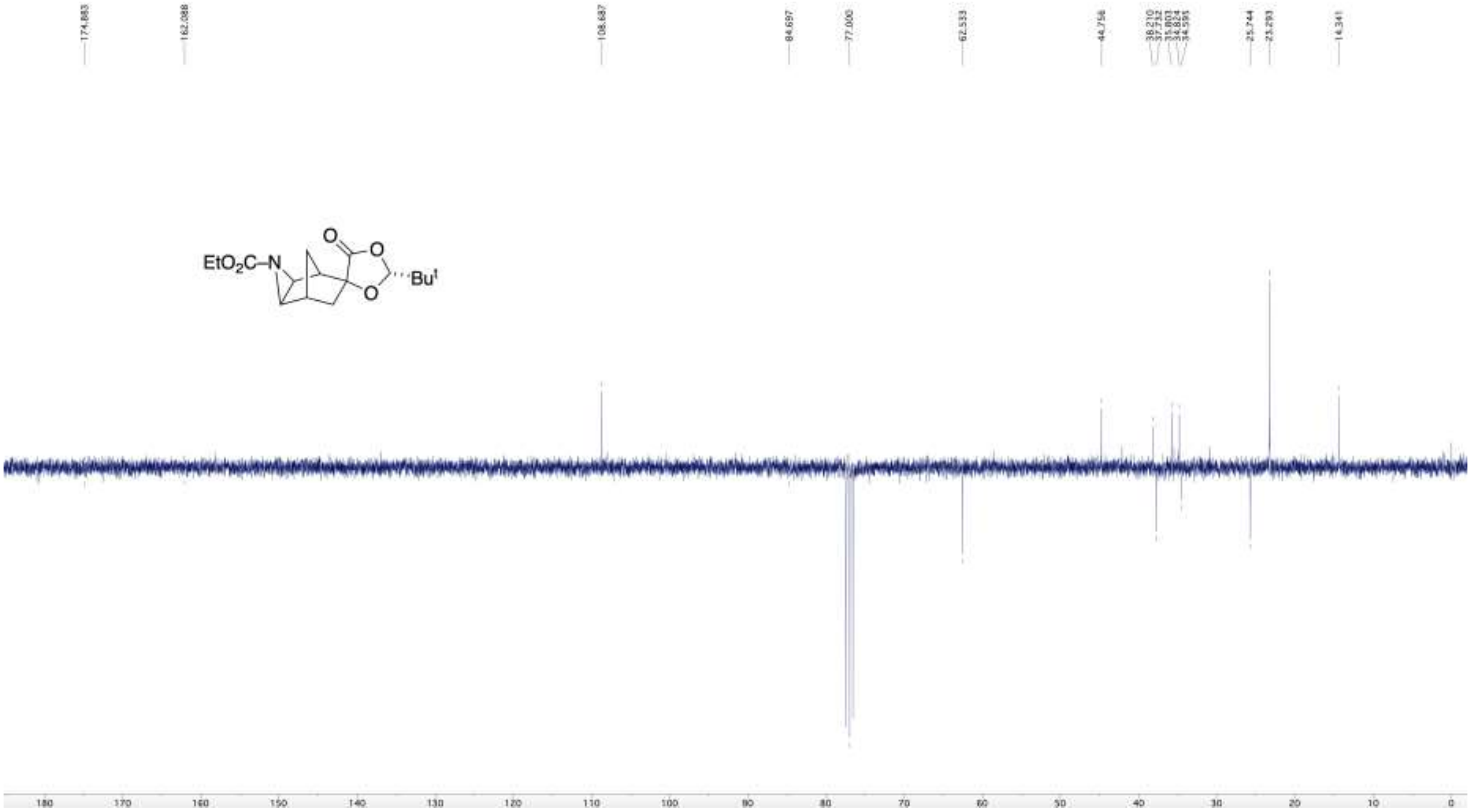

Figure S27. HRMS of **20**

Elemental Composition Report

Single Mass Analysis

Tolerance = 50.0 PPM / DBE: min = -1.5, max = 40.0

Isotope cluster parameters: Separation = 1.0 Abundance = 1.0%

Monoisotopic Mass, Odd and Even Electron Ions

65 formula(e) evaluated with 10 results within limits (up to 8 closest results for each mass)

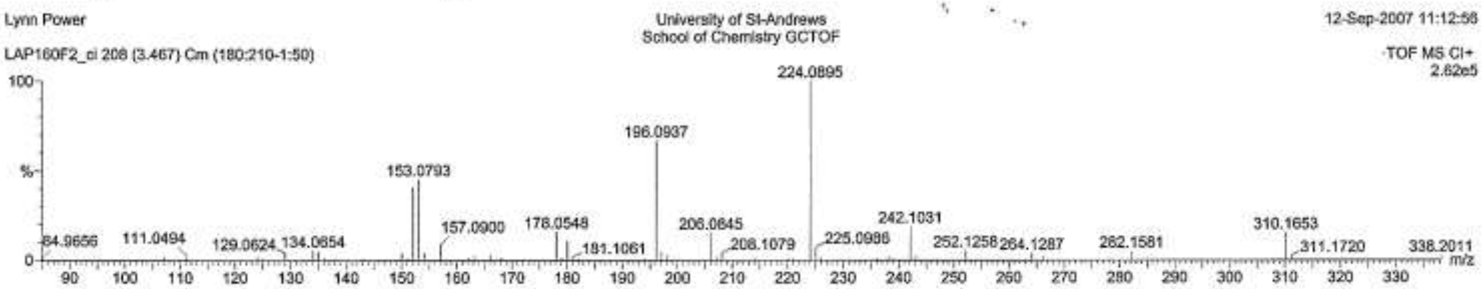

Minimum:				-1.5		
Maximum:		200.0	50.0	40.0		
Mass	Calc. Mass	mDa	PPM	DBE	Score	Formula
310.1653	310.1654	-0.1	-0.5	5.5	5	C16 H24 N O5
	310.1681	-2.8	-9.1	10.0	7	C19 H22 N2 O2
	310.1614	3.9	12.5	1.5	1	C11 H24 N3 O7
	310.1569	8.4	27.1	10.0	8	C20 H22 O3
	310.1740	-8.7	-28.1	1.0	2	C12 H26 N2 O7
	310.1556	9.7	31.4	10.5	6	C18 H20 N3 O2
	310.1767	-11.4	-36.7	5.5	4	C15 H24 N3 O4
	310.1529	12.4	40.1	6.0	3	C15 H22 N2 O5

Figure S28. 300 MHz ^{1}H NMR spectrum of **21**

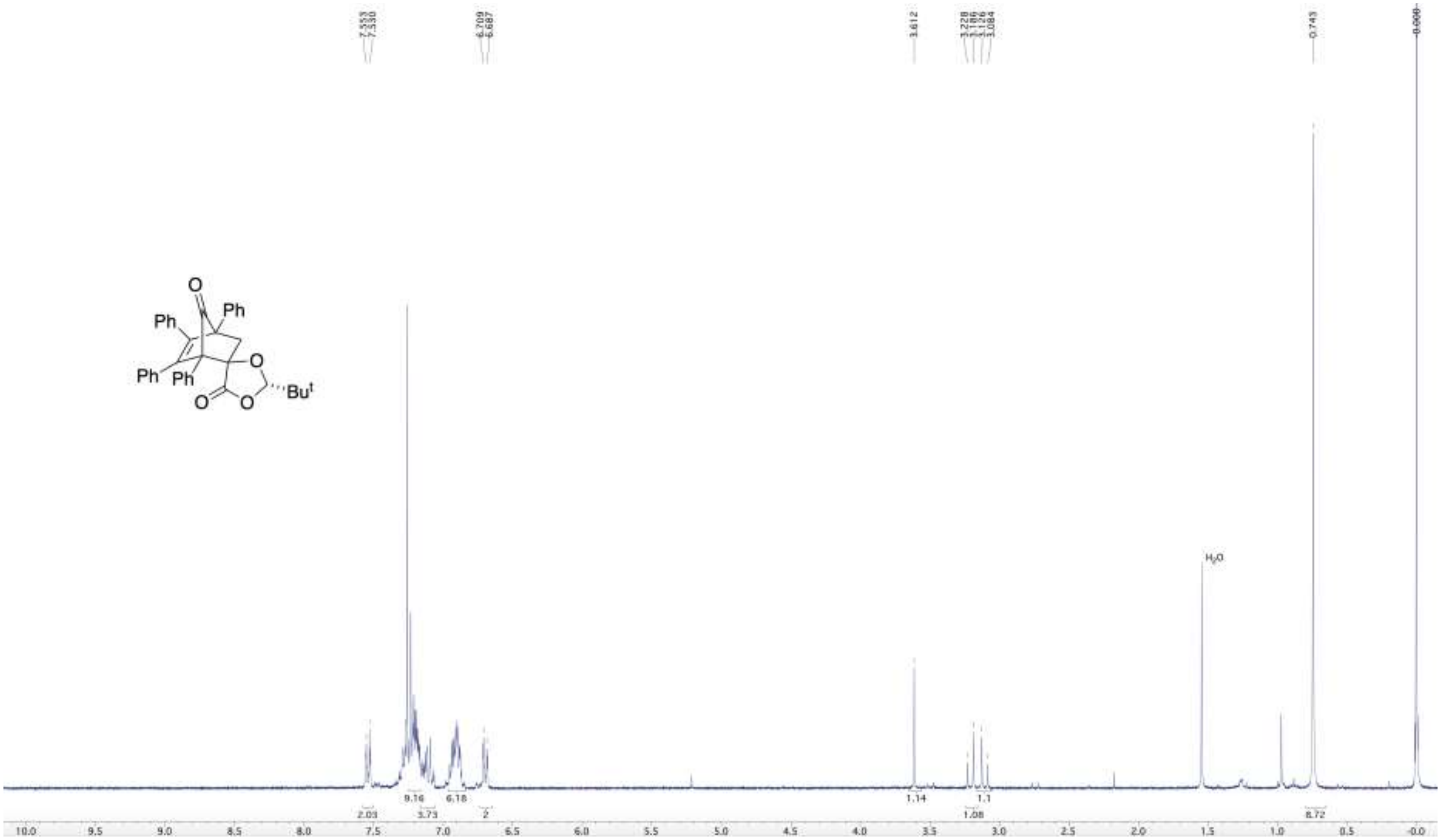

Figure S29. 75 MHz DEPTQ ^{13}C NMR spectrum of **21**

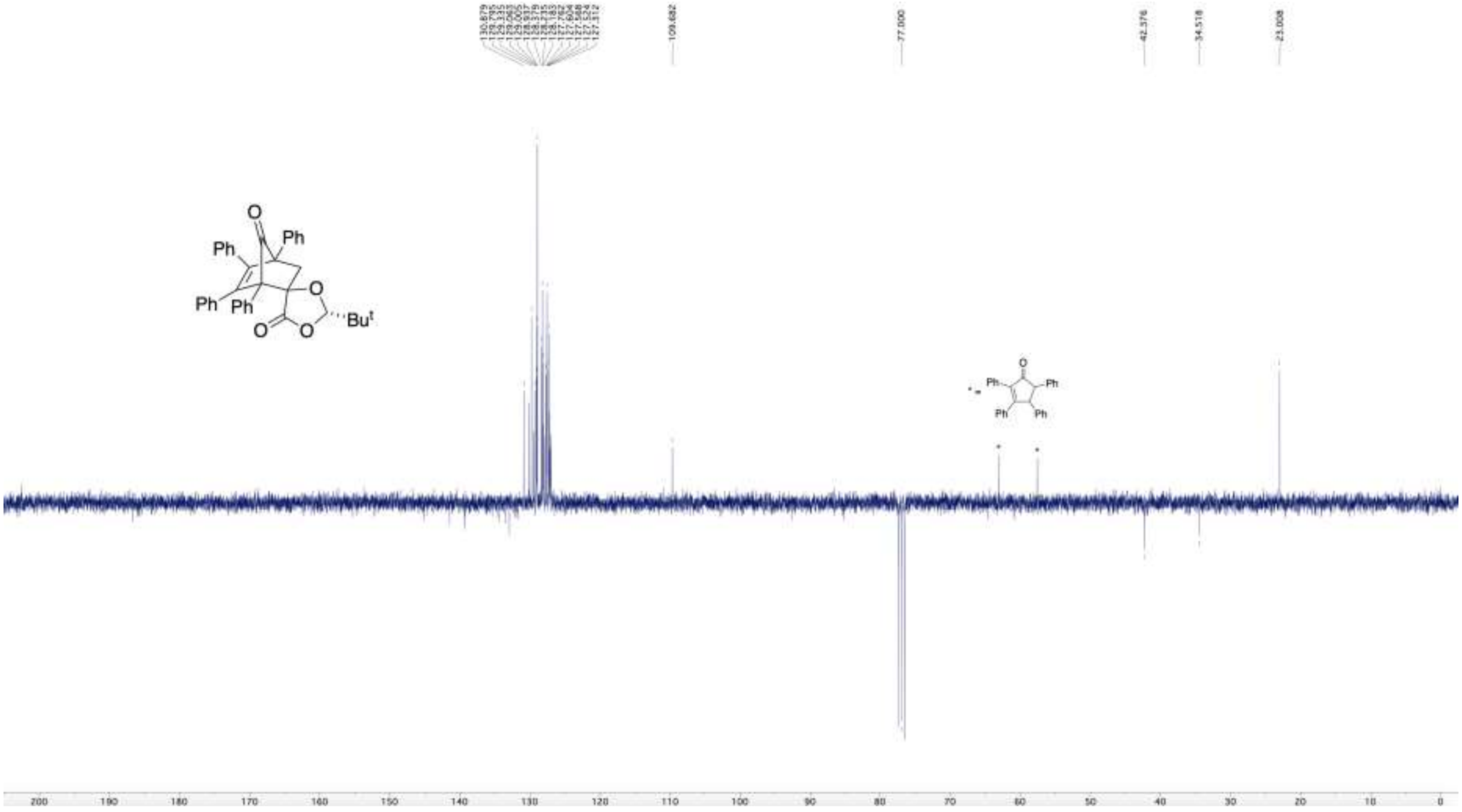

Figure S30. IR spectrum of **21**

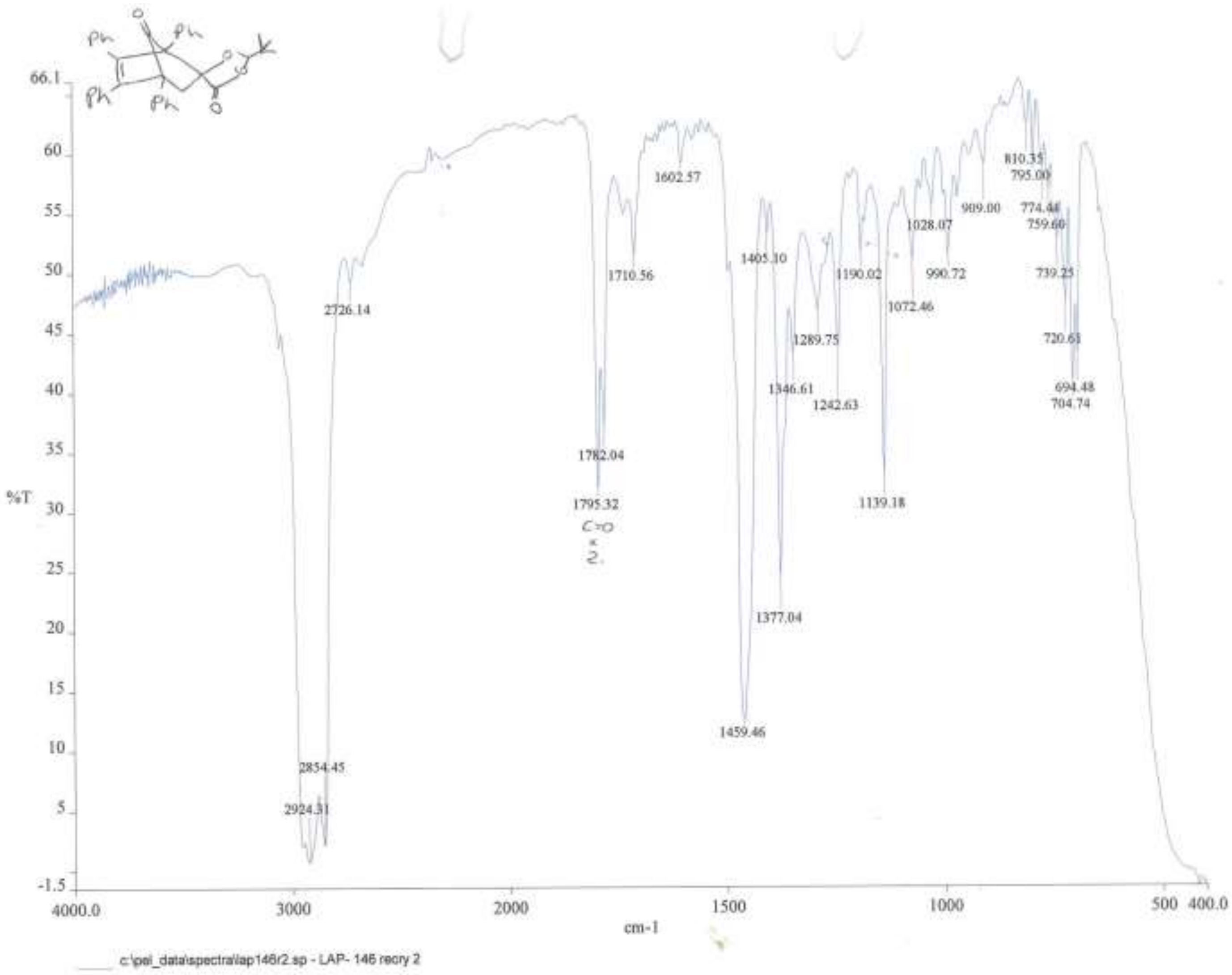

Figure S31. HRMS of **21**

Elemental Composition Report **Page 1**

Single Mass Analysis

Tolerance = 50.0 PPM / DBE: min = -1.5, max = 40.0

Isotope cluster parameters: Separation = 1.0 Abundance = 1.0%

Monoisotopic Mass, Odd and Even Electron Ions

27 formula(e) evaluated with 5 results within limits (up to 8 closest results for each mass)

Lynn Power University of St-Andrews School of Chemistry LCTOF 10-Mar-2006 10:24:05

LAP146fr6_14am3 21 (0.402) AM (Cen,2, 80.00, Ar,7000.0,556.28,0.76,LS 5); Cm (21:30) 1: TOF MS ES+ 1.20e3

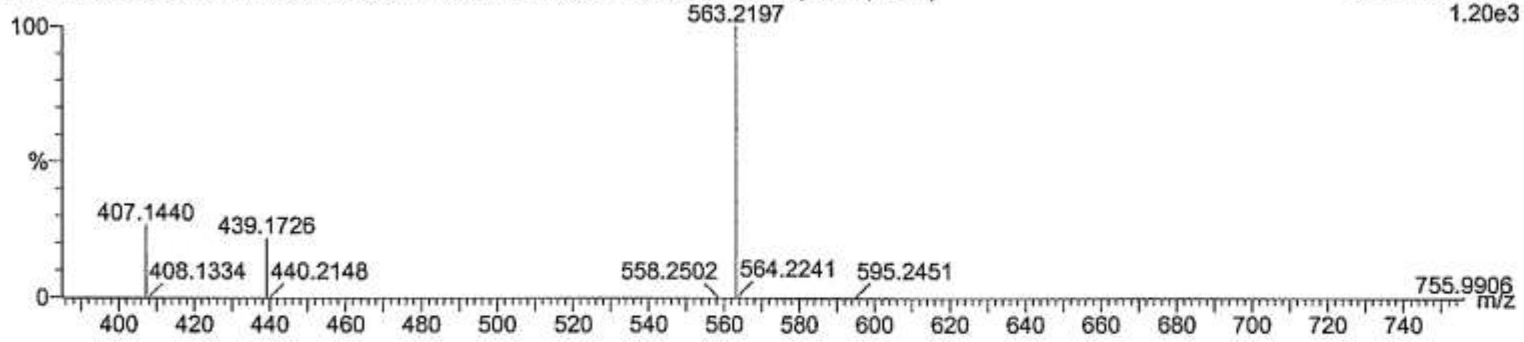

Minimum:				-1.5		
Maximum:		200.0	50.0	40.0		
Mass	Calc. Mass	mDa	PPM	DBE	Score	Formula
563.2197	563.2198	-0.2	-0.3	21.5	3	C37 H32 O4 Na
	563.2222	-2.6	-4.6	24.5	4	C39 H31 O4
	563.1987	21.0	37.2	26.5	5	C40 H28 O2 Na
	563.2410	-21.3	-37.8	16.5	1	C34 H36 O6 Na
	563.2434	-23.7	-42.1	19.5	2	C36 H35 O6

Figure S32. 300 MHz ^{1}H NMR spectrum of **22a**

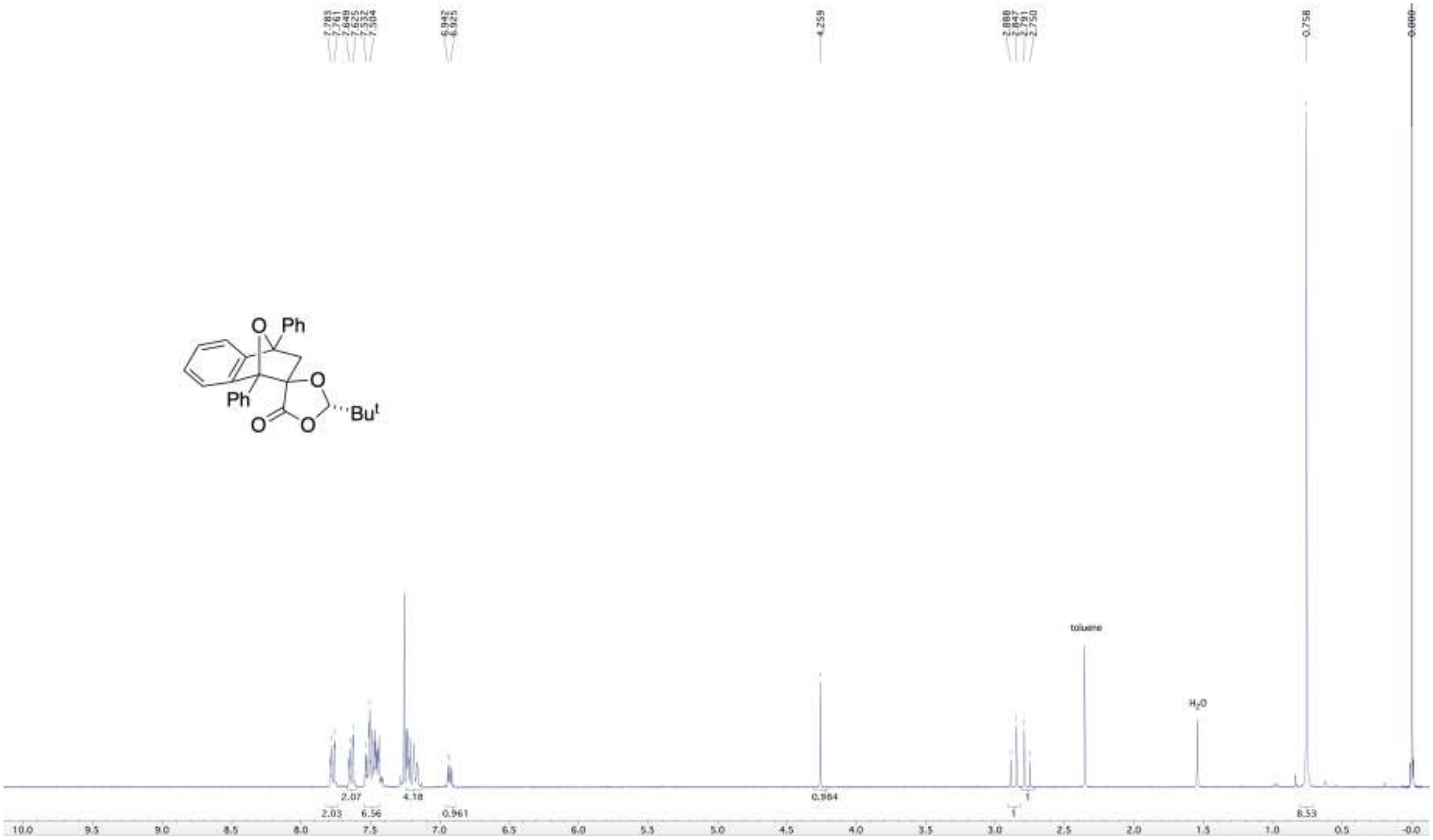

Figure S33. 75 MHz DEPTQ ^{13}C NMR spectrum of **22a**

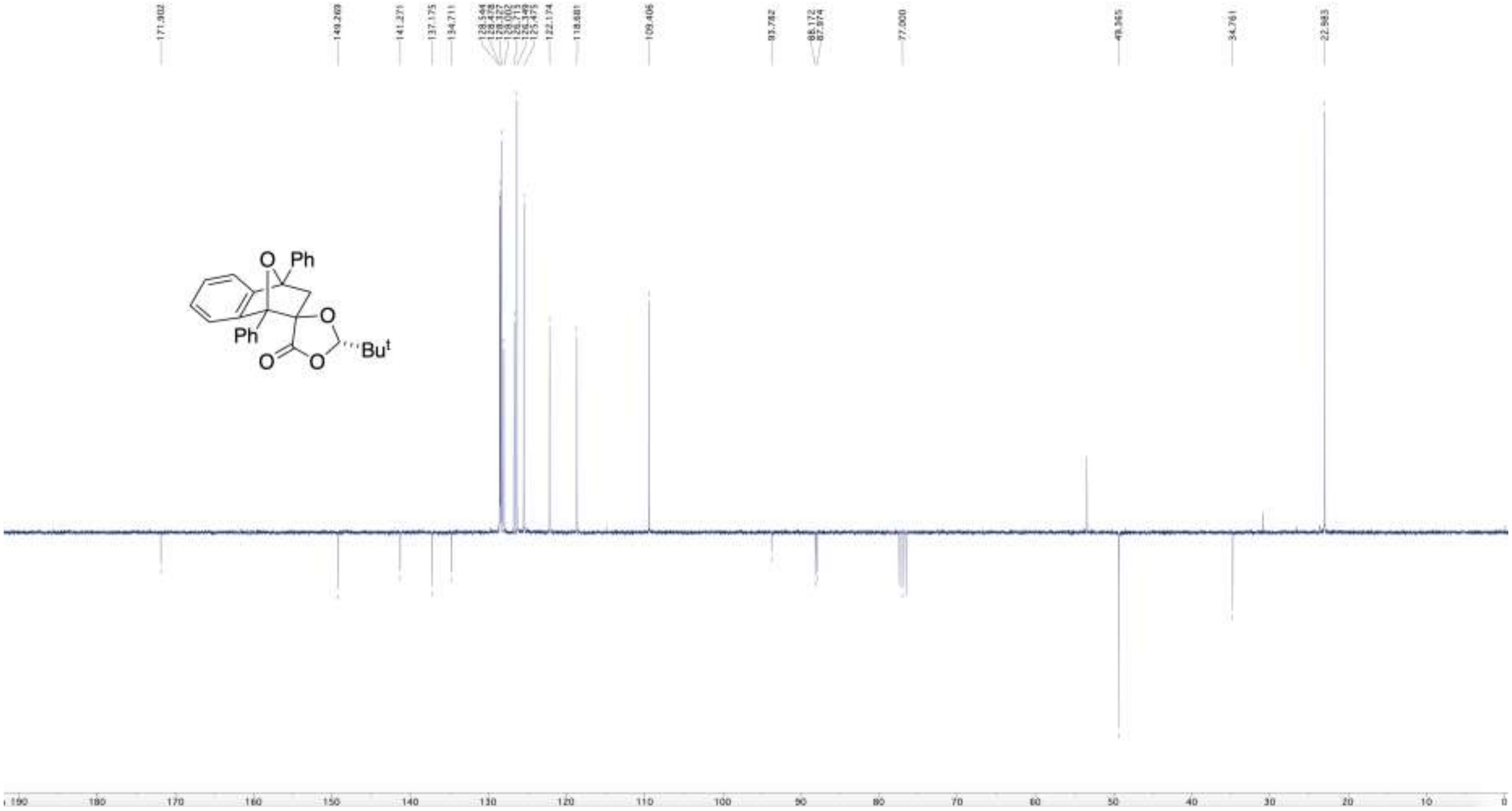

Figure S34. IR spectrum of **22a**

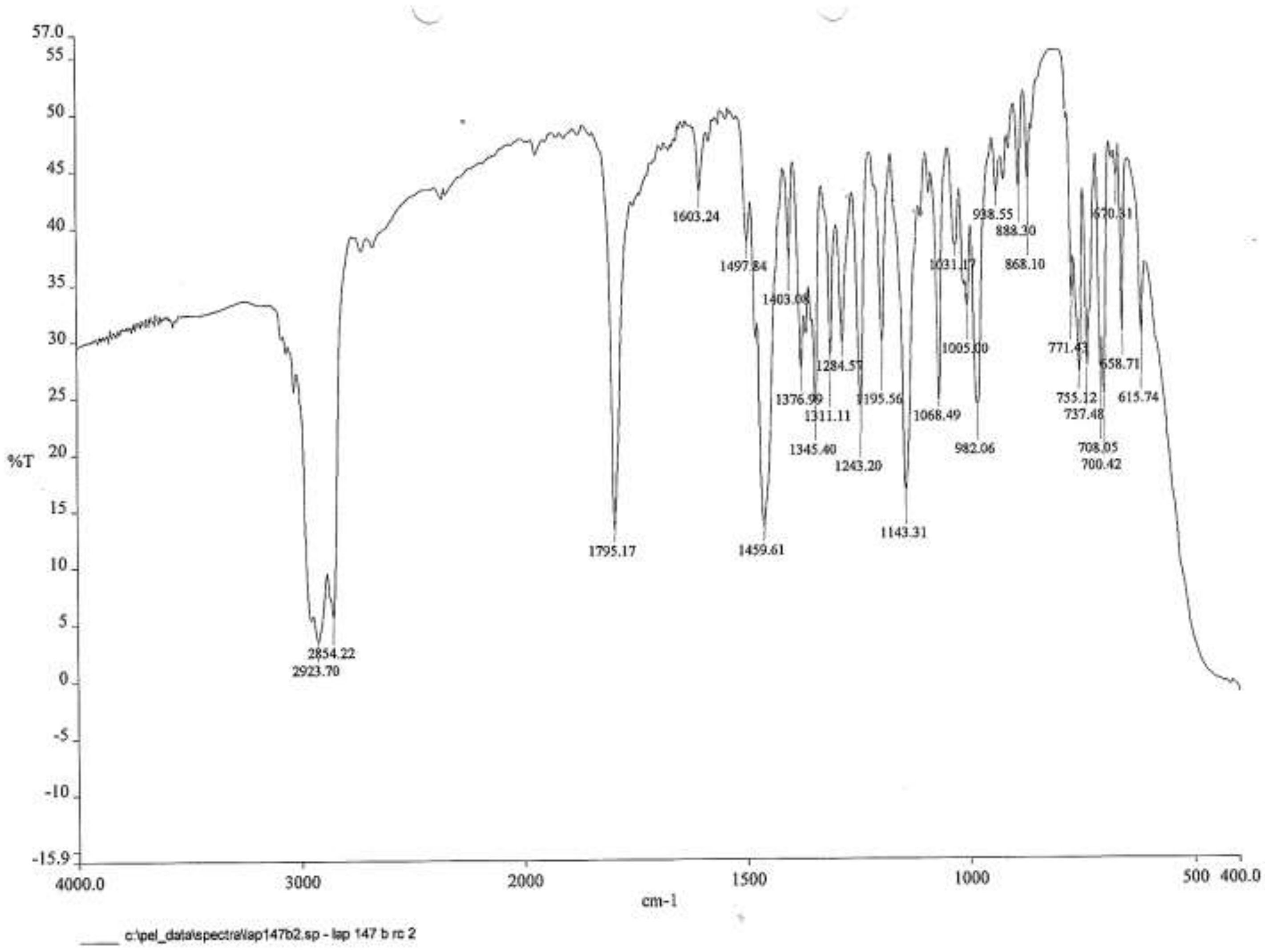

Figure S35. 300 MHz ^{1}H NMR spectrum of **24**

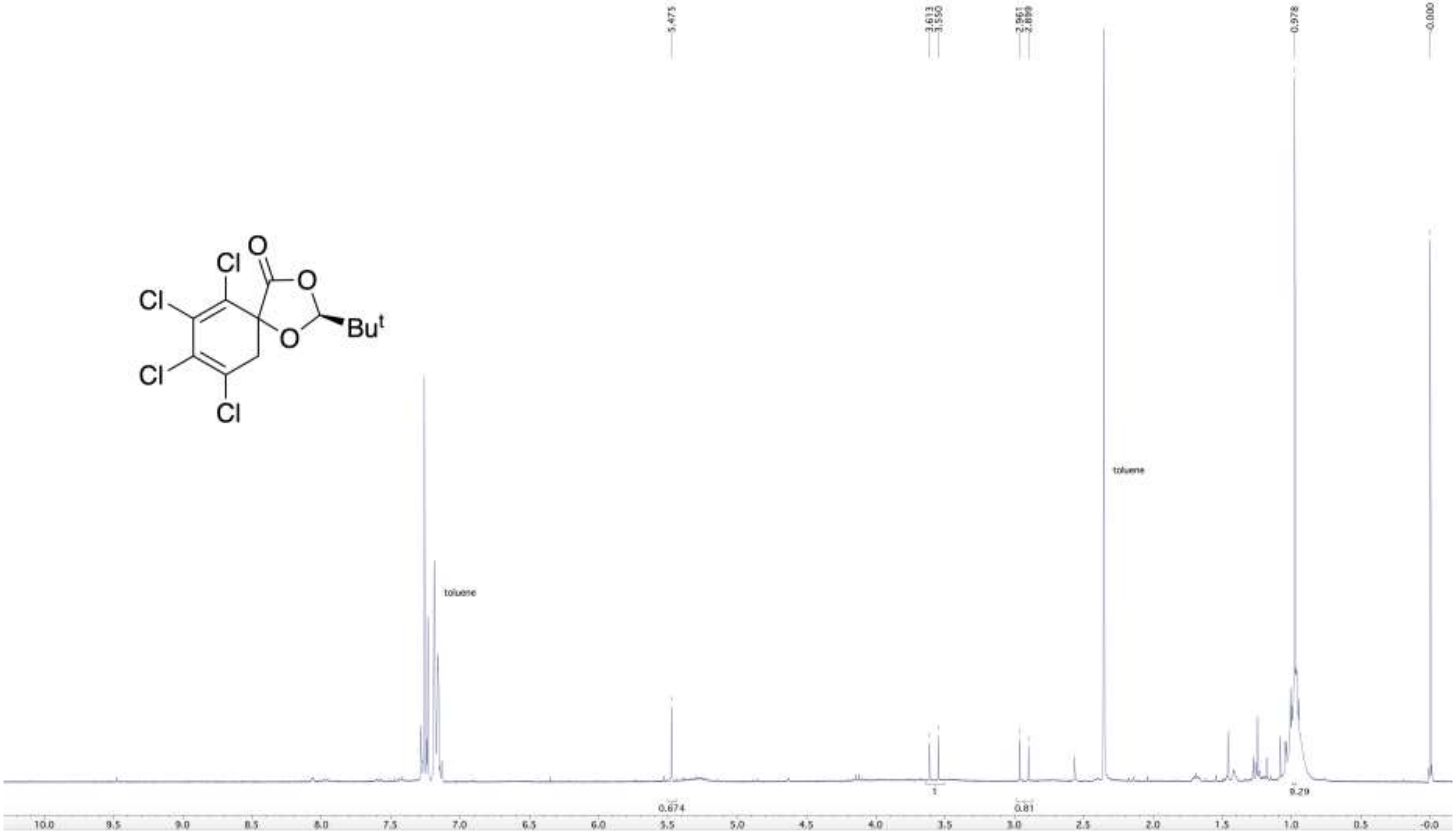

Figure S36. 75 MHz DEPTQ ^{13}C NMR spectrum of **24**

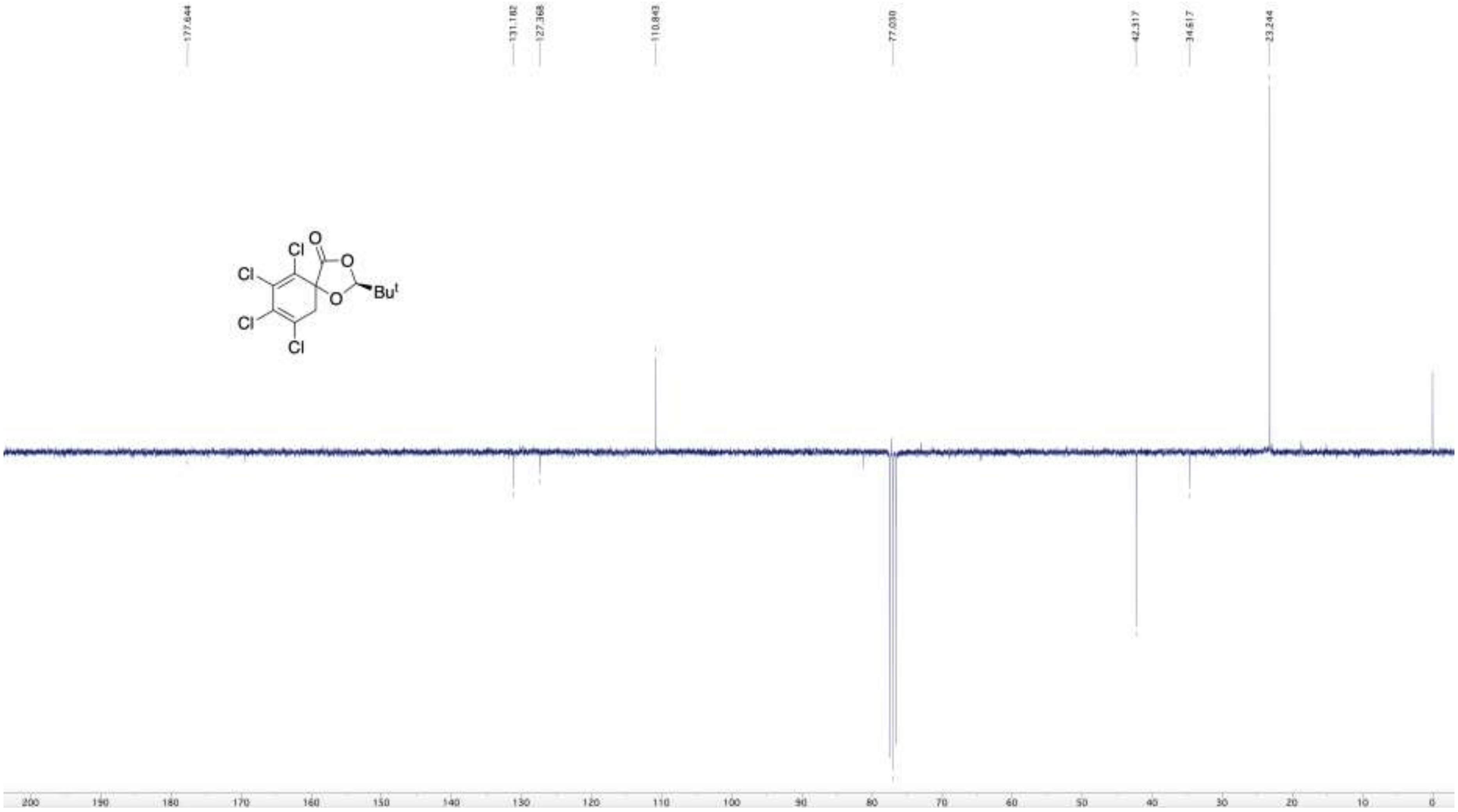

Figure 37. IR spectrum of **24**

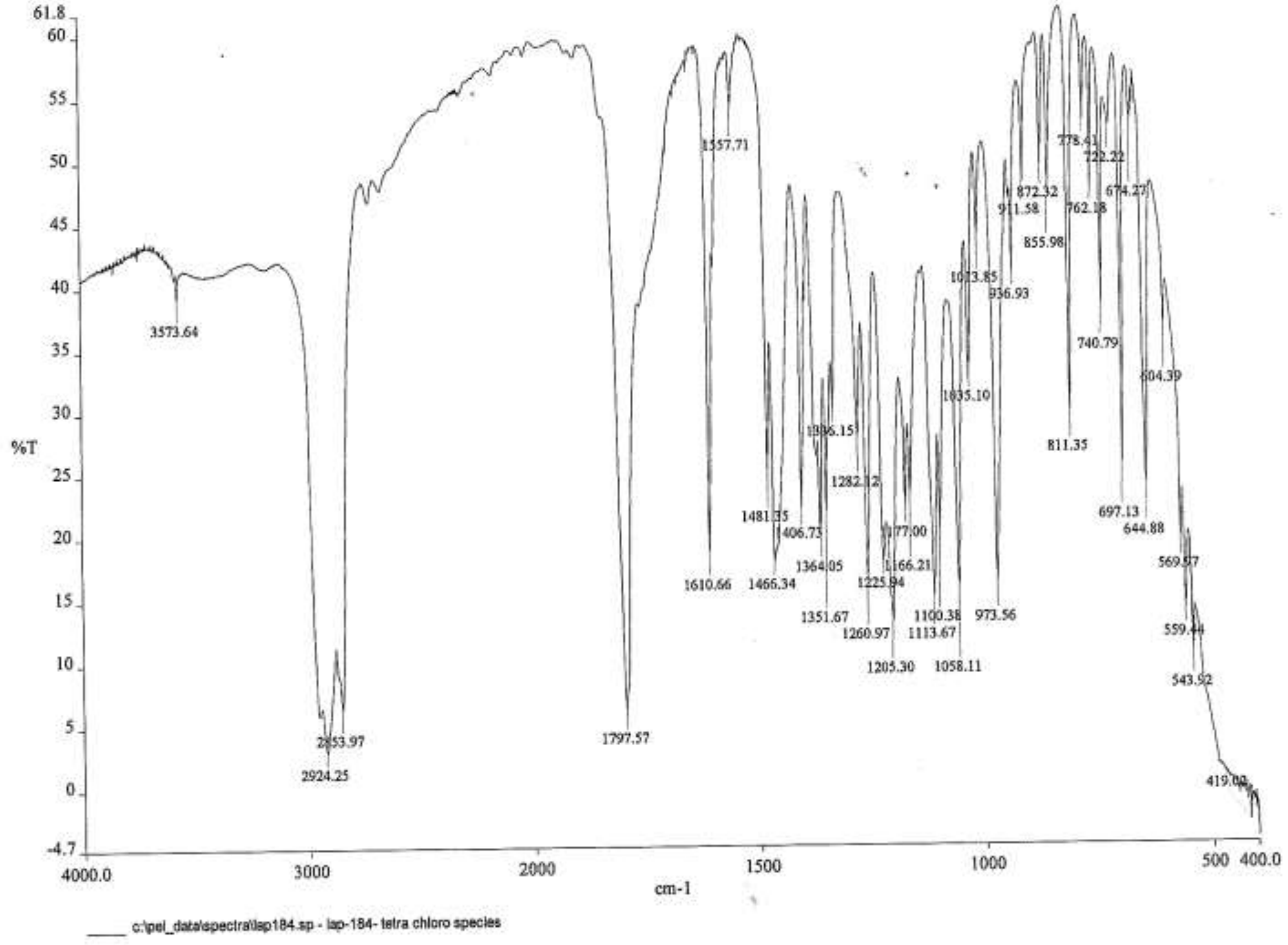

Figure S38. 300 MHz ^{1}H NMR spectrum of **25**

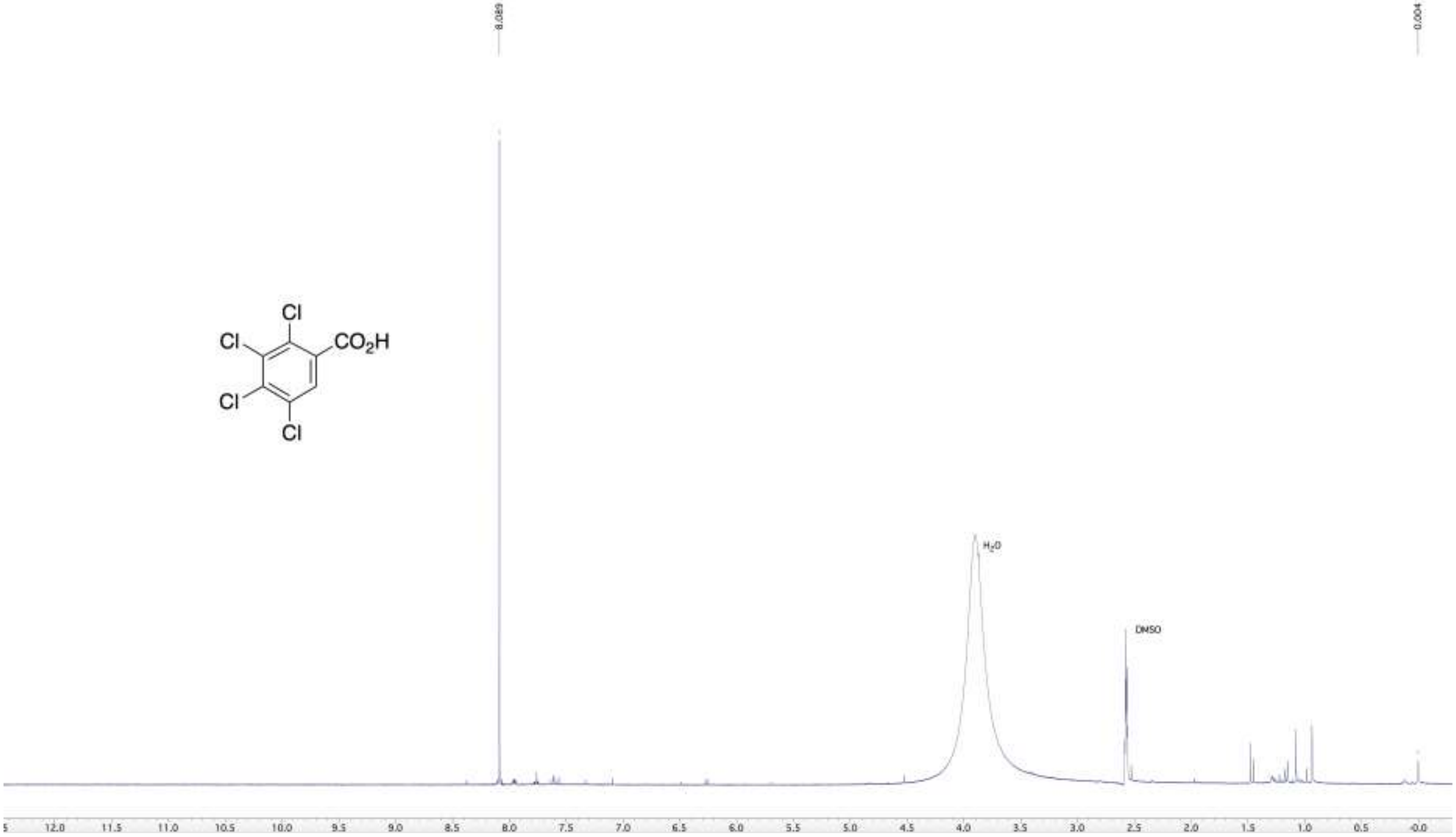

Figure S39. 75 MHz DEPTQ ^{13}C NMR spectrum of **25**

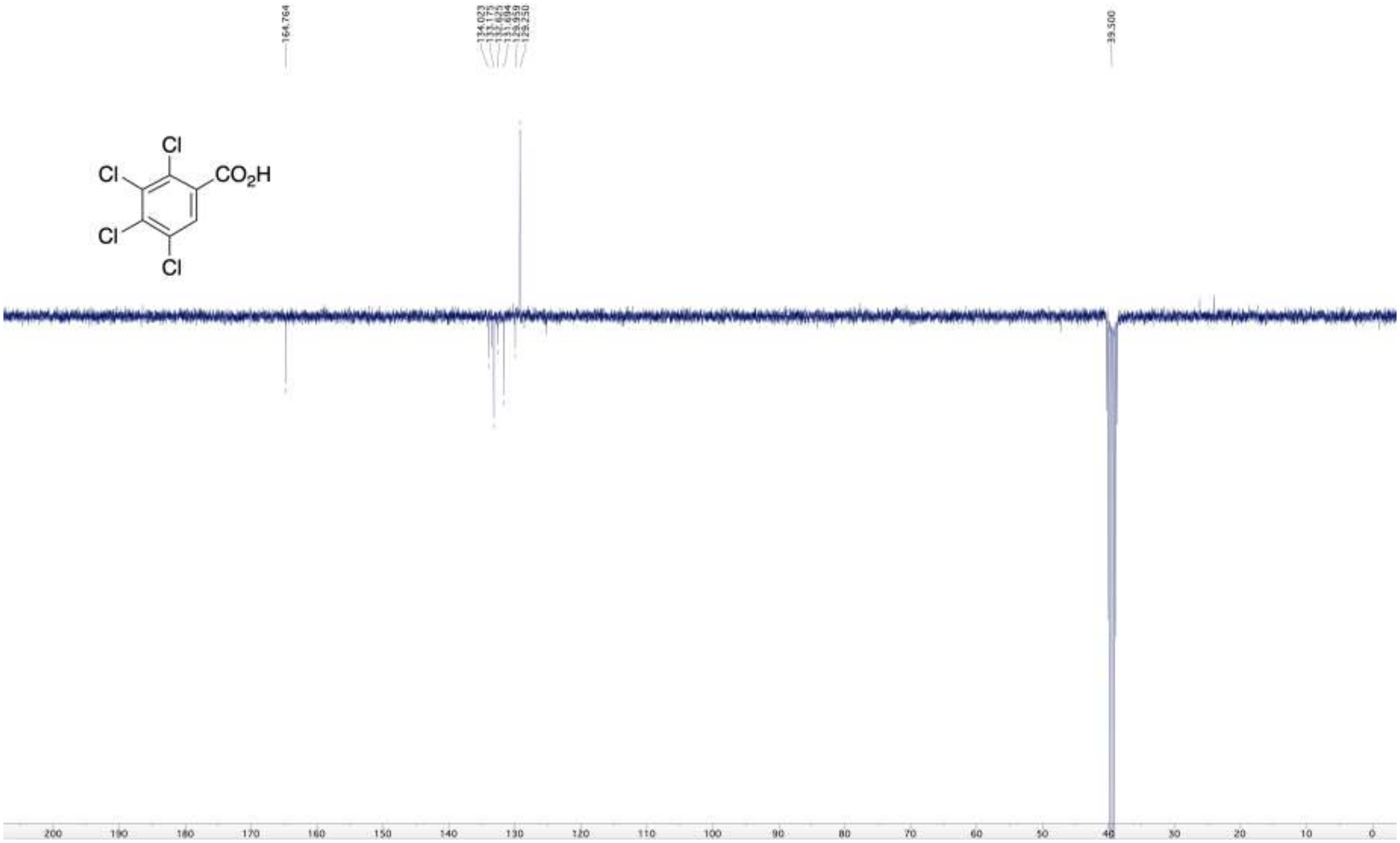

Figure S40. 300 MHz ^{1}H NMR spectrum of **27**

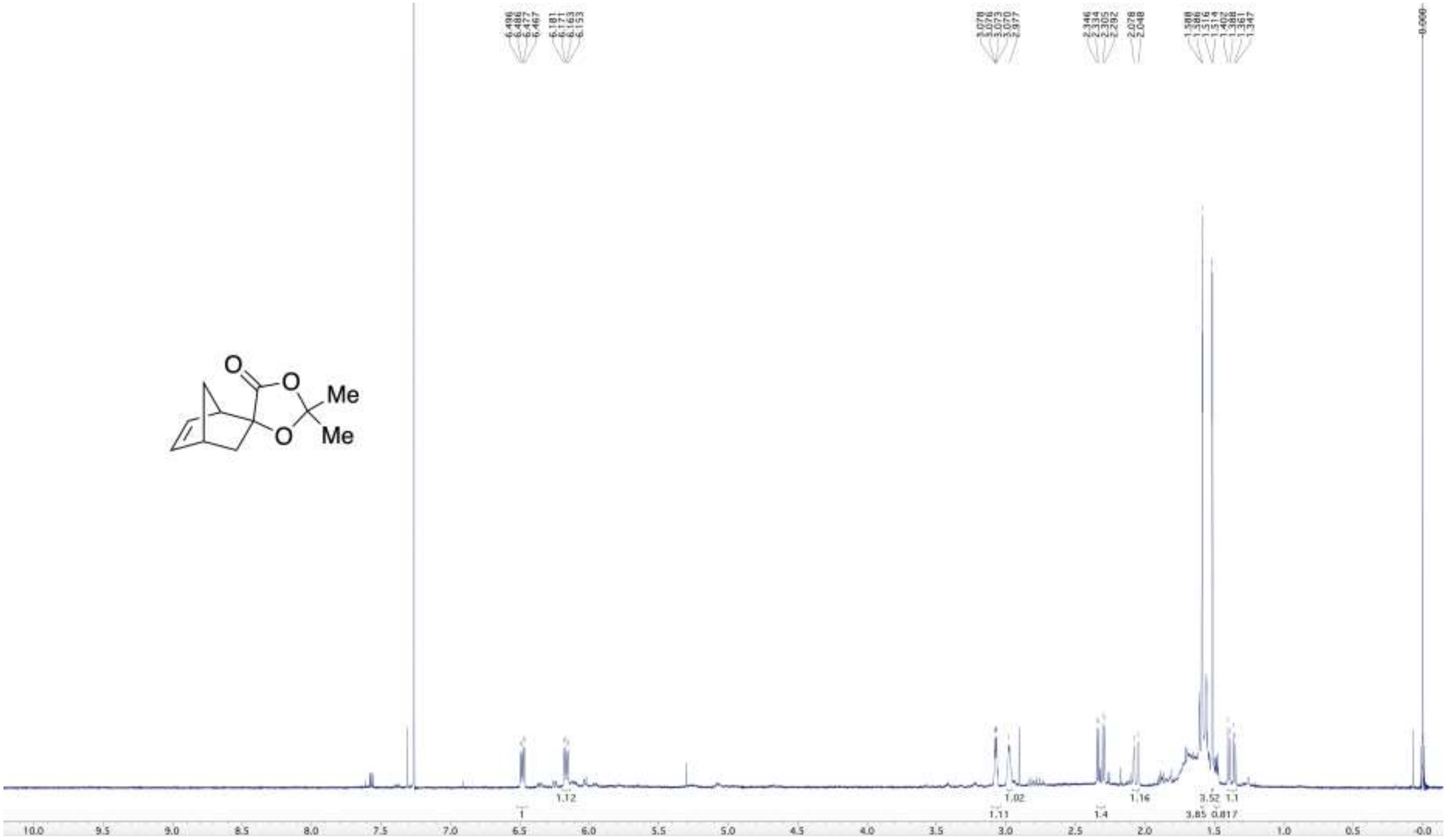

Figure S41. 75 MHz DEPTQ ^{13}C NMR spectrum of **27**

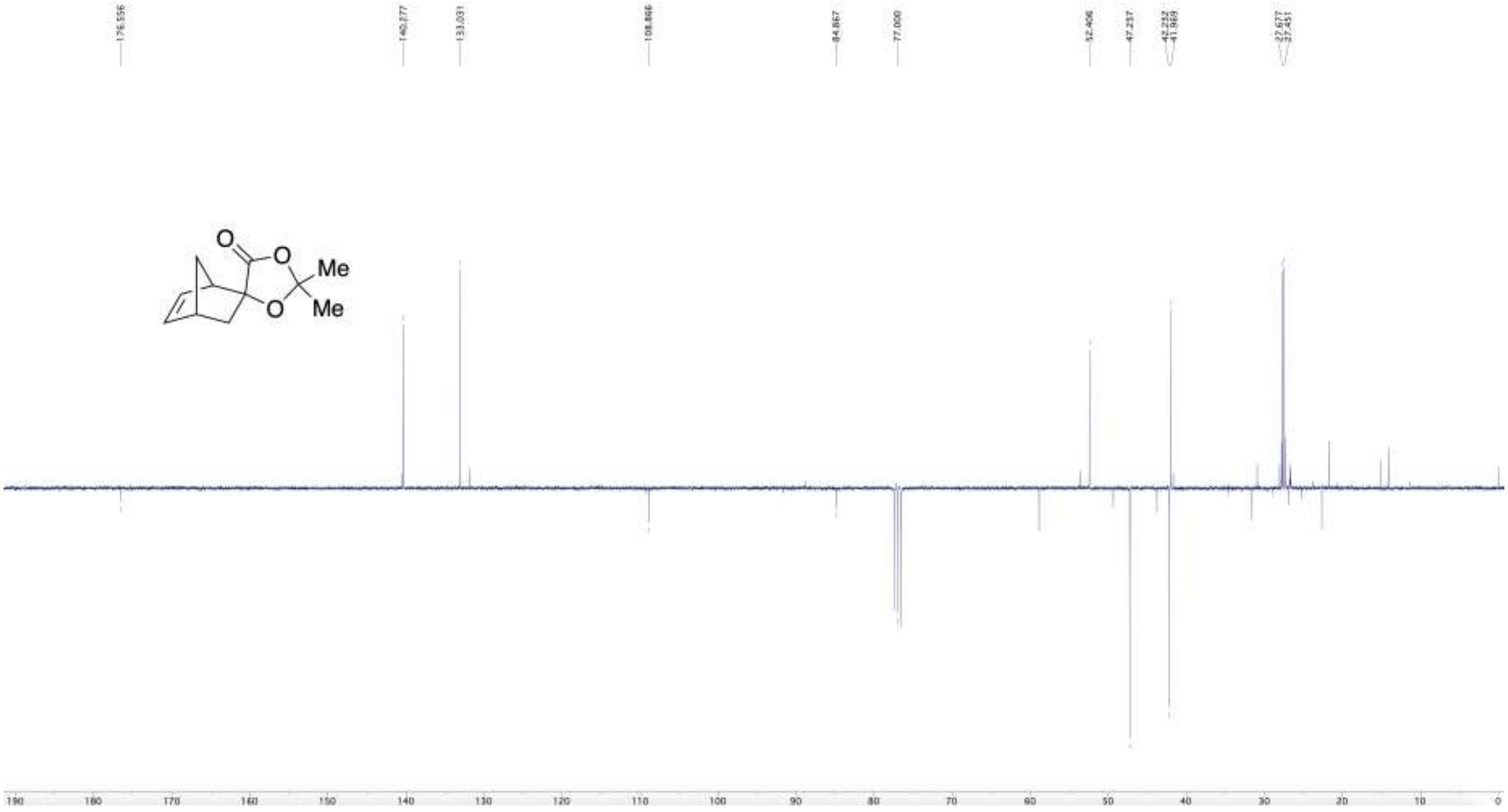

Figure S42. 300 MHz ^{1}H NMR spectrum of **28**

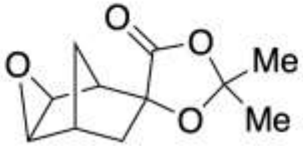

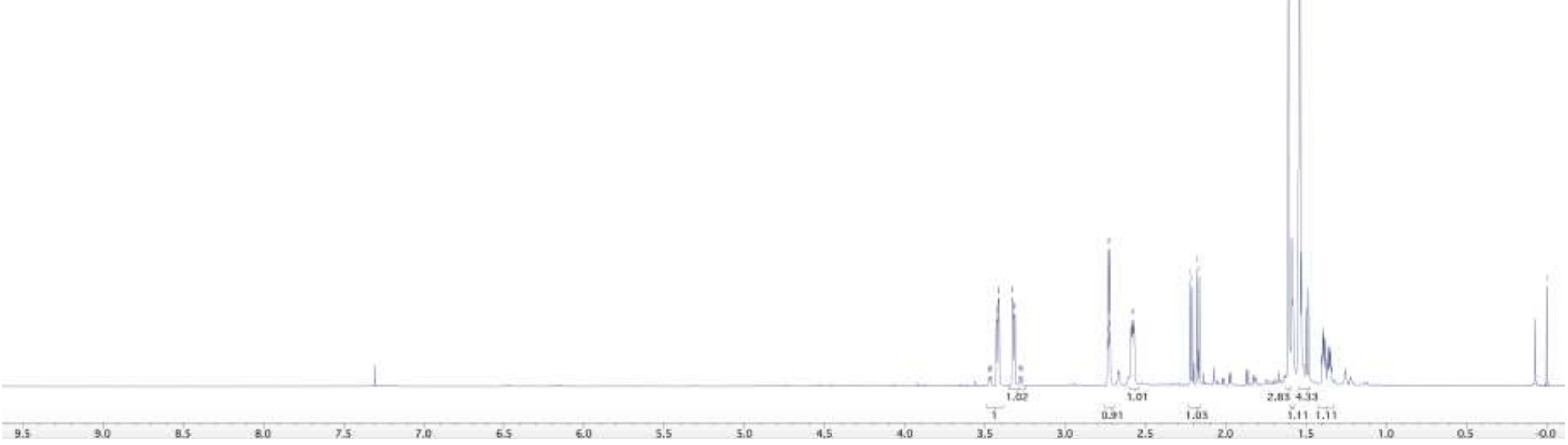

Figure S43. 75 MHz DEPTQ ^{13}C NMR spectrum of **28**

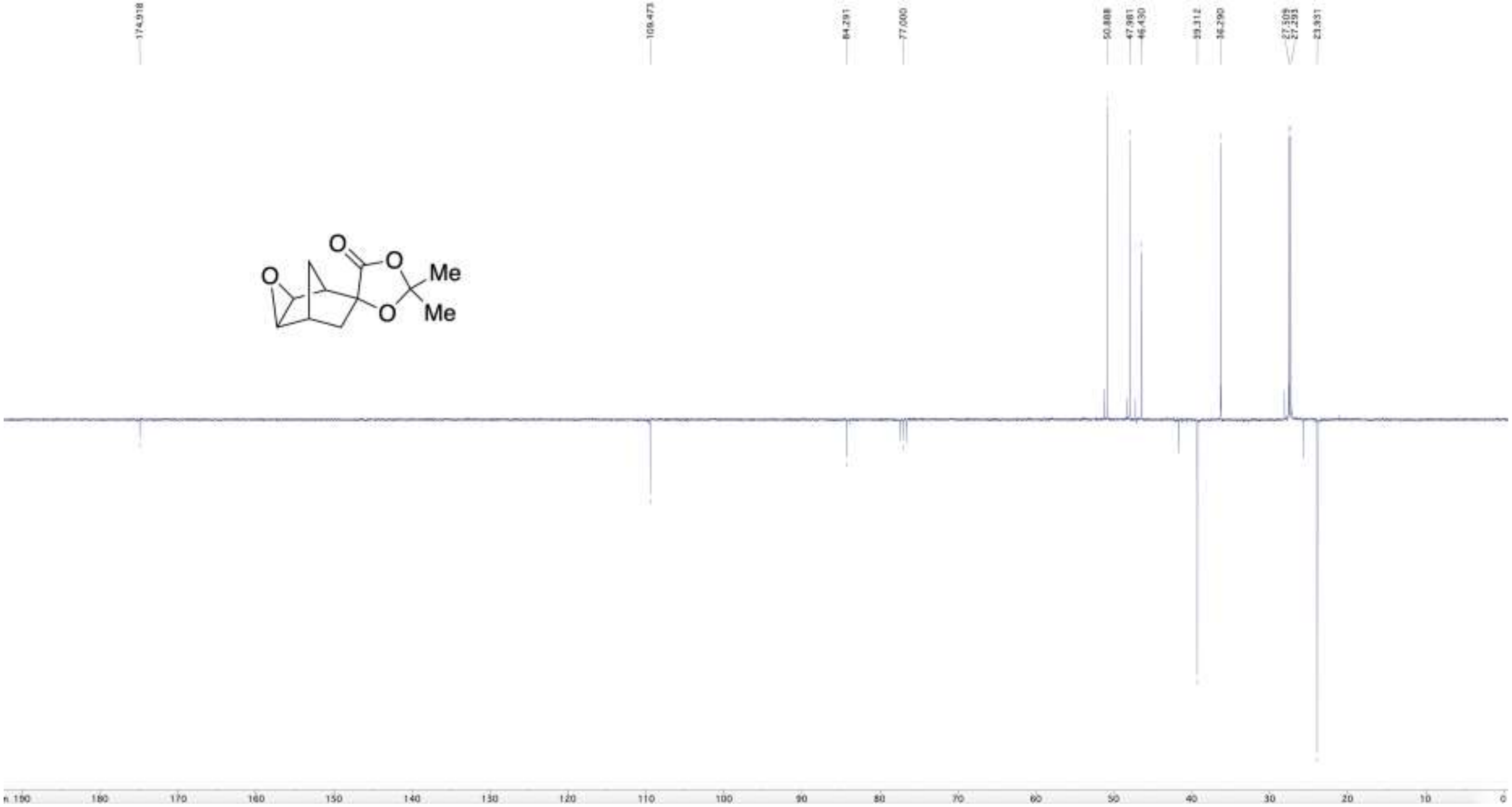

Figure S44. HRMS of **28**

Elemental Composition Report

Single Mass Analysis

Tolerance = 20.0 PPM / DBE: min = -1.5, max = 40.0
Isotope cluster parameters: Separation = 1.0 Abundance = 1.0%

Monoisotopic Mass, Odd and Even Electron Ions
18 formula(e) evaluated with 1 results within limits (up to 8 closest results for each mass)

Lynn Power

University of St-Andrews
School of Chemistry GCTOF

LAP172_ci 177 (2.934) Cm (159:180-1:40)

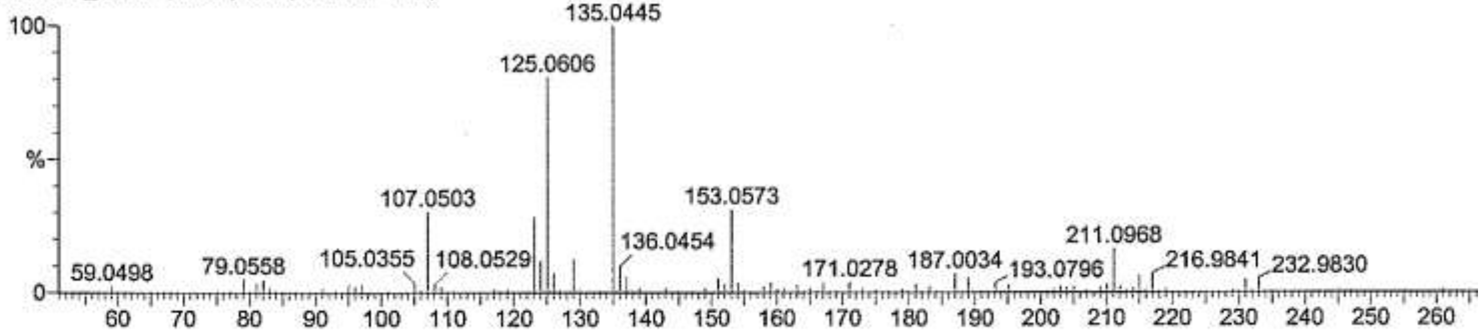

Minimum:				-1.5		
Maximum:		200.0	20.0	40.0		
Mass	Calc. Mass	mDa	PPM	DBE	Score	Formula
211.0968	211.0970	-0.2	-1.1	4.5	1	C11 H15 O4

Figure S45. 300 MHz ^{1}H NMR spectrum of **29a**

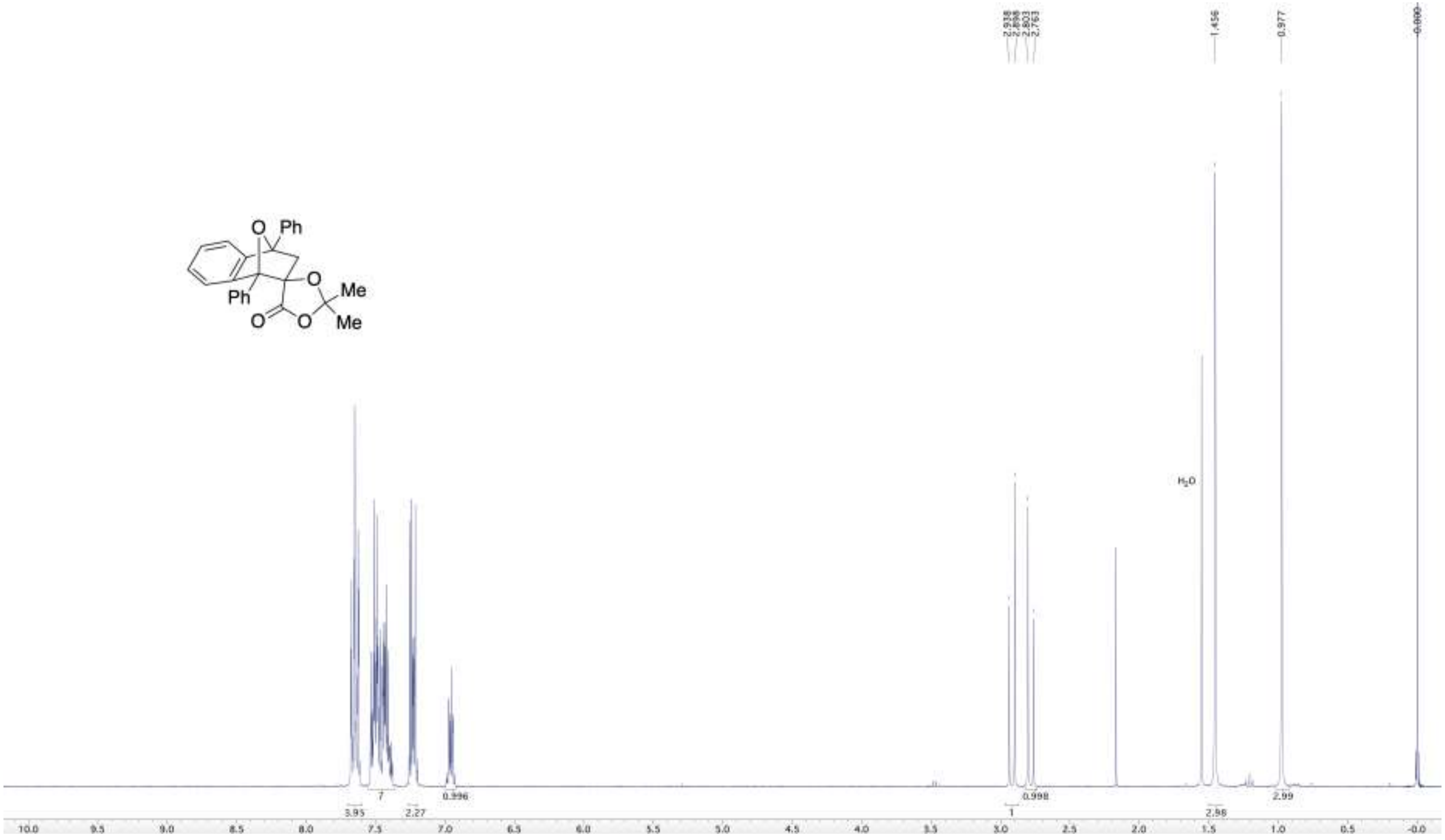

Figure S46. 75 MHz DEPTQ ^{13}C NMR spectrum of **29a**

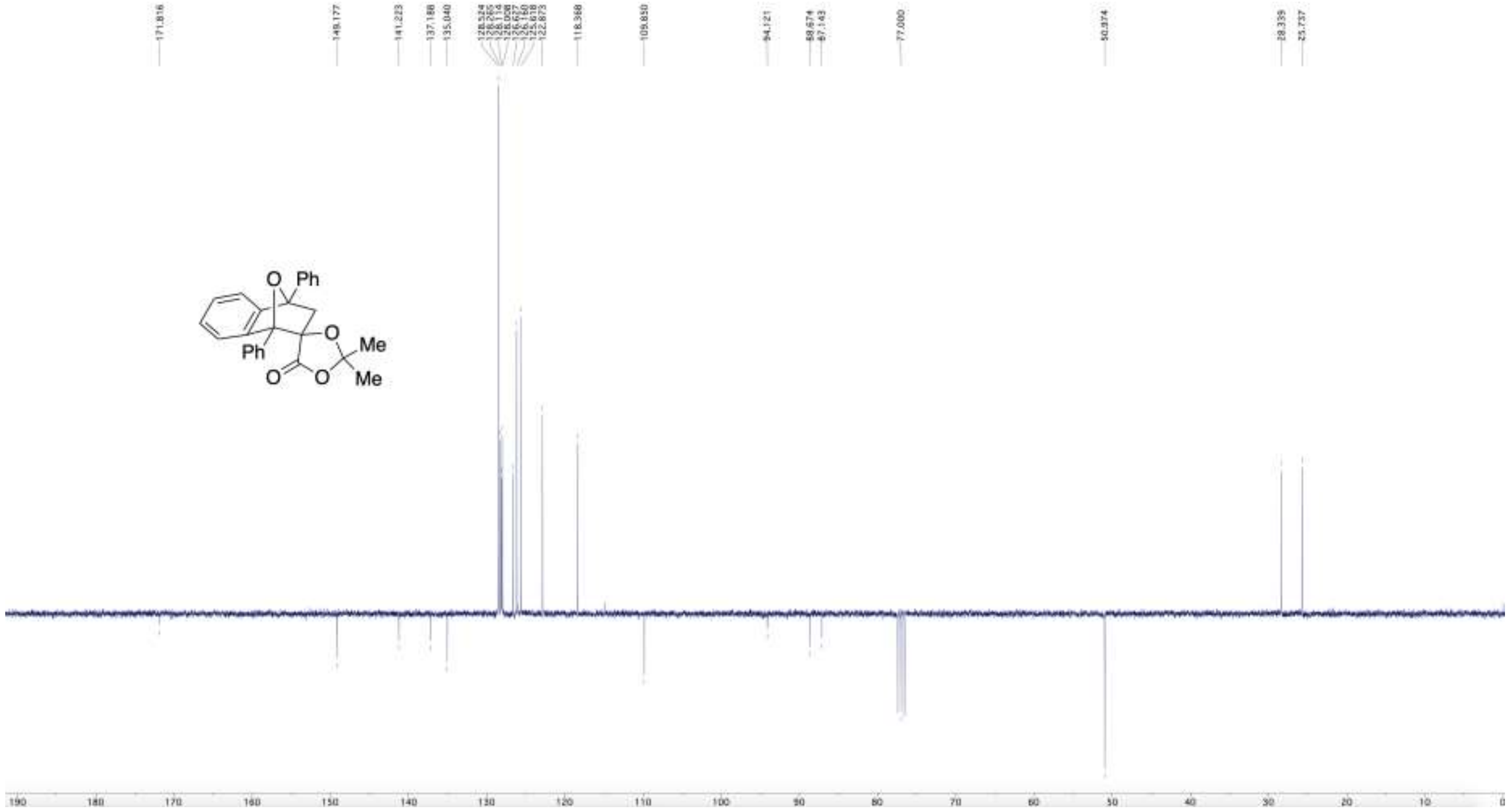

Figure S47. IR spectrum of **29a**

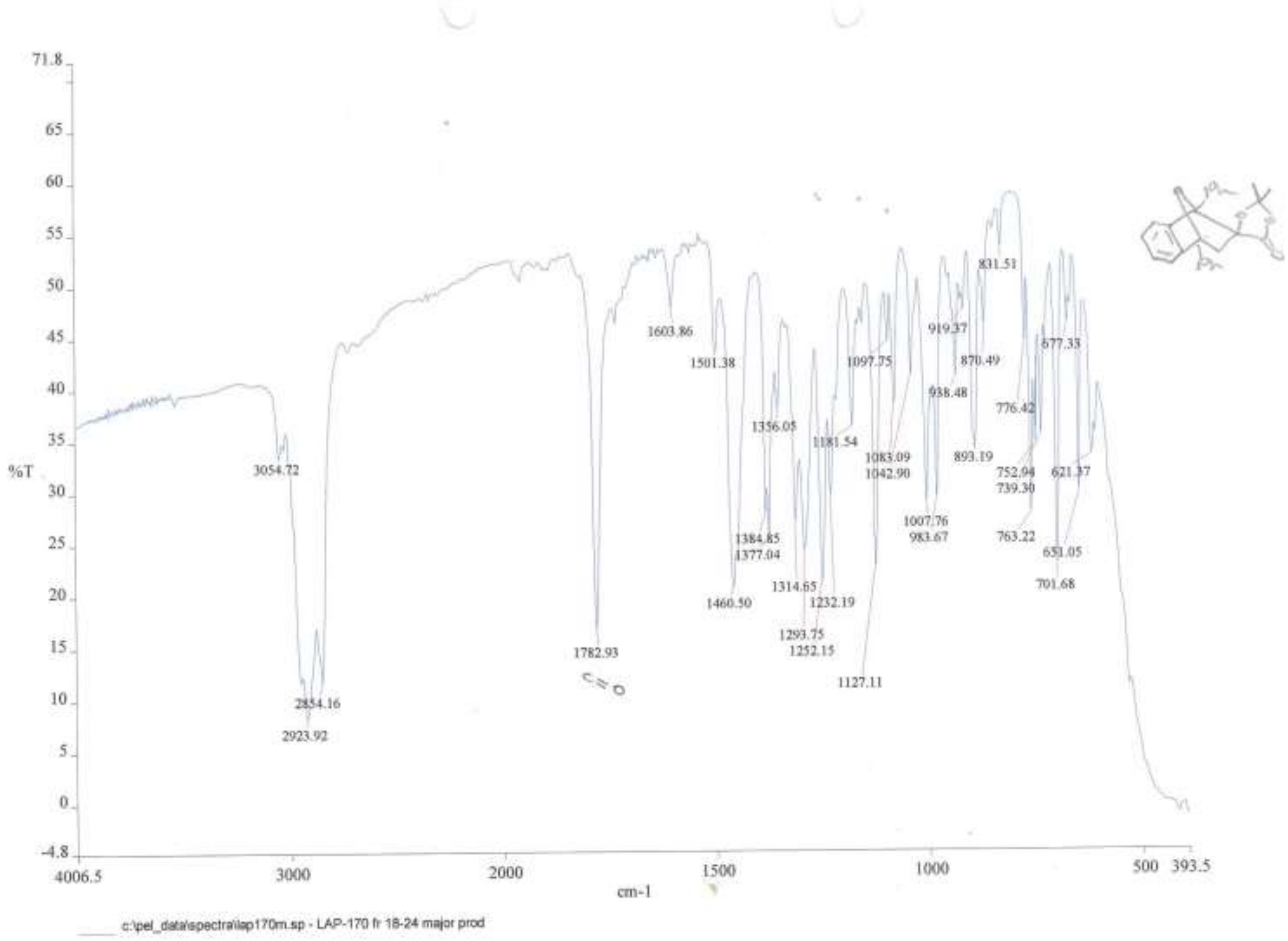

Figure S48. 300 MHz ^{1}H NMR spectrum of **29b**

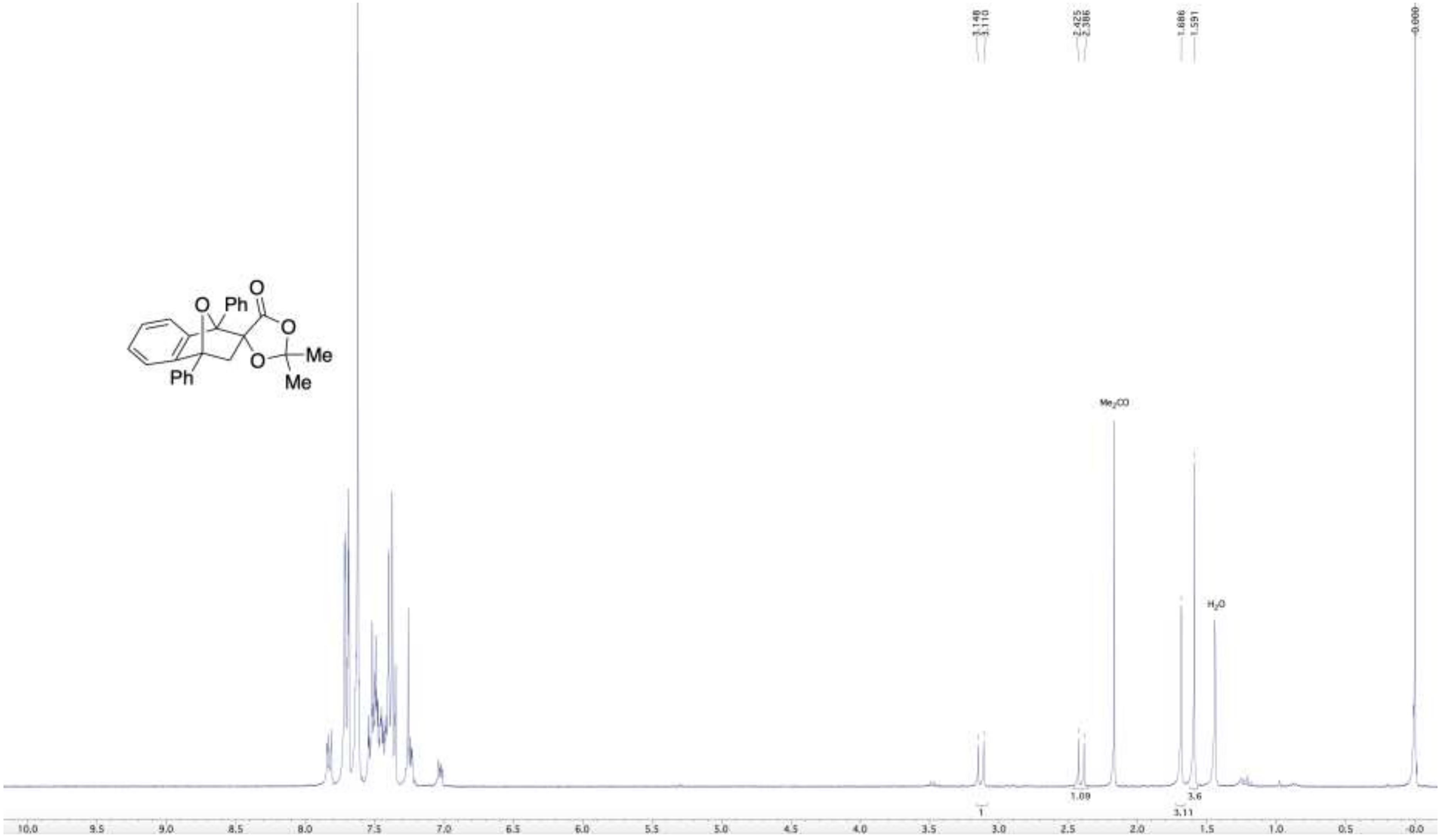

Figure S49. IR spectrum of **29b**

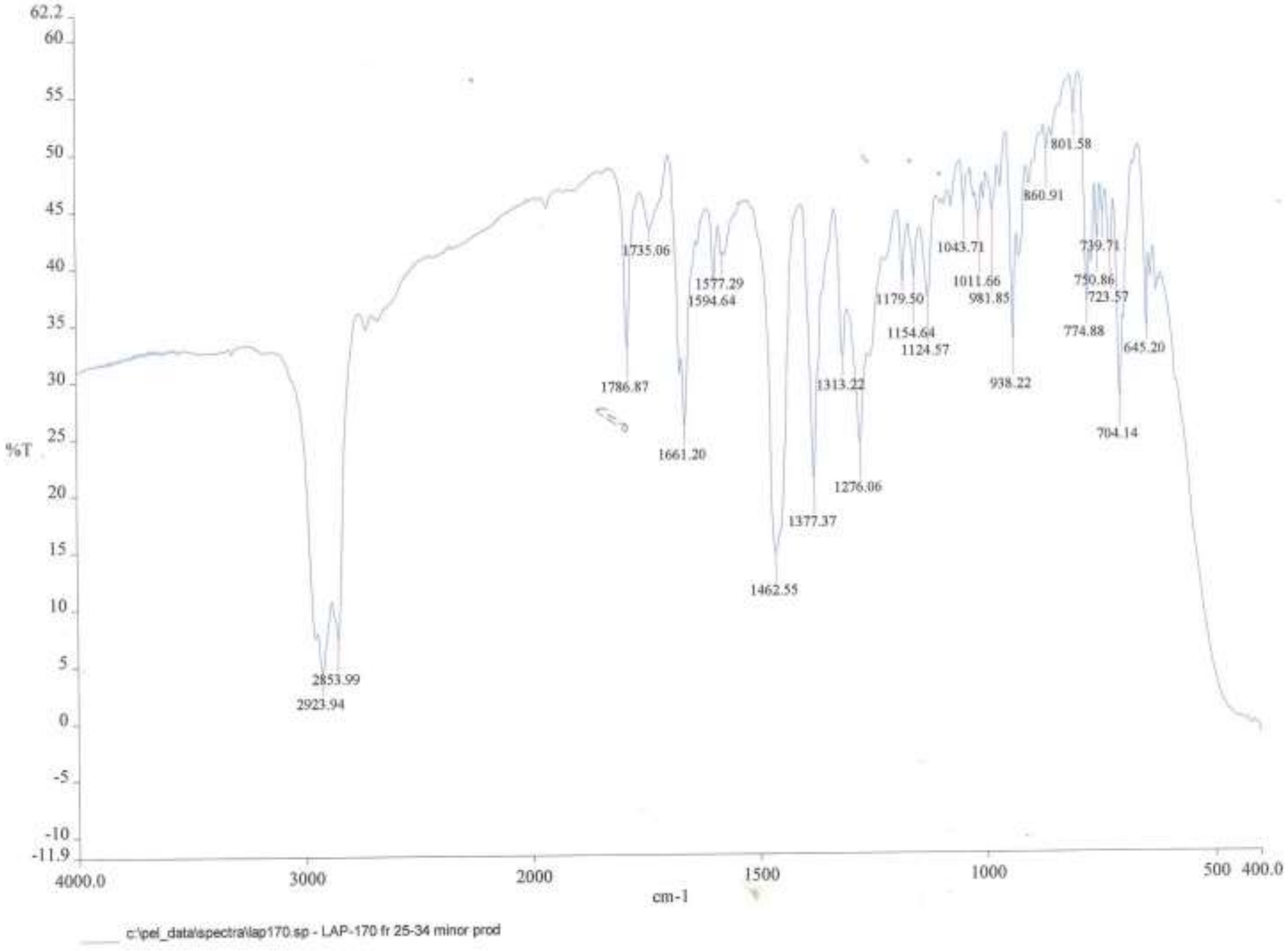

Supplement: Supplementary file 1 [file molecules-28-03845-s001.zip › molecules-2344076-supplementary.pdf]
